# Supplementary material for: Single-cell and spatial multi-omics highlight effects of anti-integrin therapy across cellular compartments in ulcerative colitis
Source: Nat Commun. 2024 Feb 19;15:1493. doi: 10.1038/s41467-024-45665-6 (PMC10876948; doi:10.1038/s41467-024-45665-6)
Supplement: Supplementary file 1 — Suppplementary Information [file 41467_2024_45665_MOESM1_ESM.pdf]

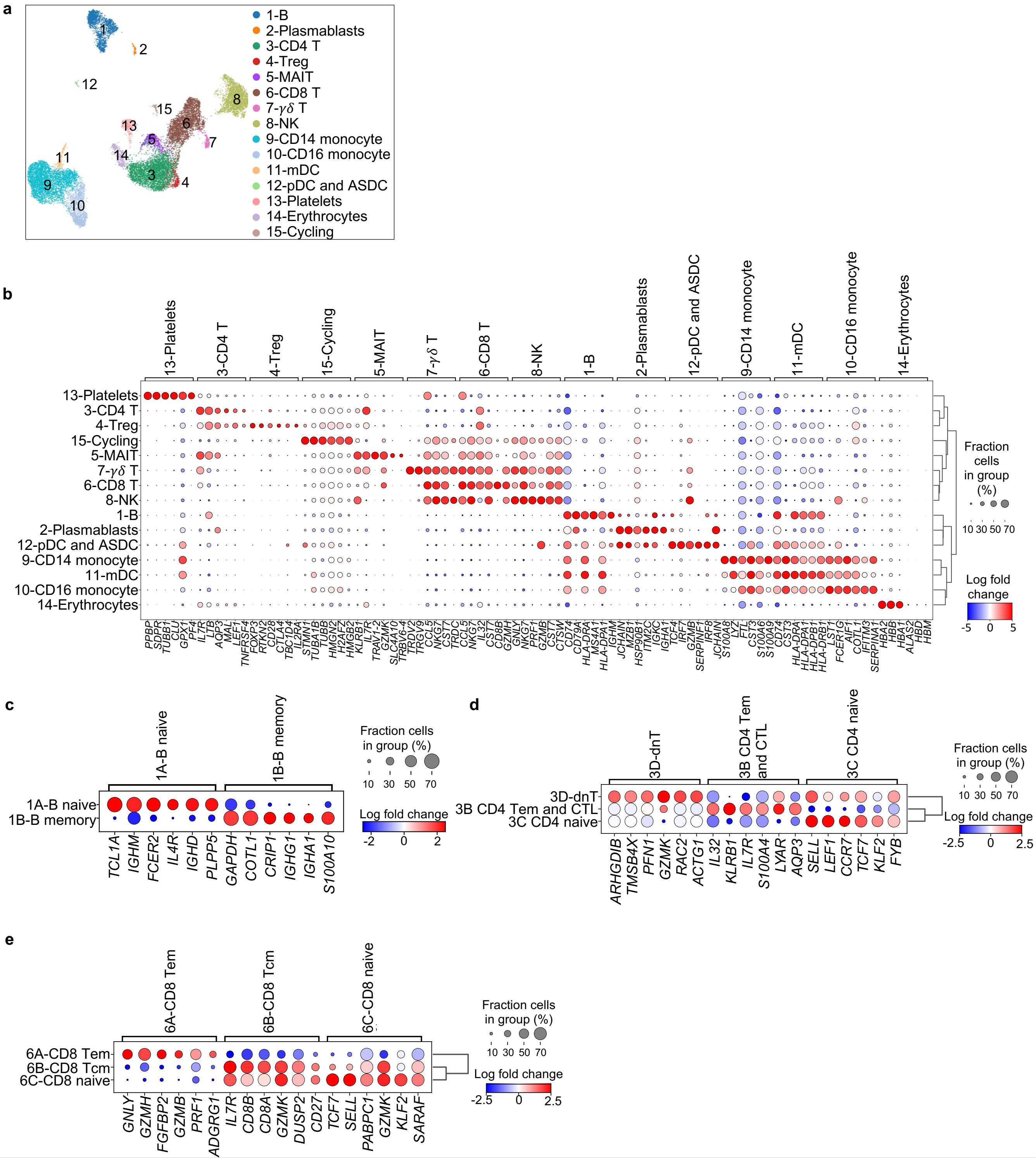

Supplementary Fig.1| Peripheral blood leukocyte (PBL) scRNA-seq coarse cell subset annotations and both coarse and fine cell subset landmark genes. a, UMAP visualization of pooled multiplex PBL scRNA-seq for 20,130 cells from HC (n=4), UC (n=3), and UC-VDZ (n=4) patients highlighting coarse cell subset annotations. **b-e,** Dot plot representation of landmark genes expressed by (**b**) coarse cell subsets and (**c-e**) fine PBL cell subsets. Genes ranked by Wilcoxon rank-sum. Cell subsets without landmark genes above selected minimum log fold change are not displayed.

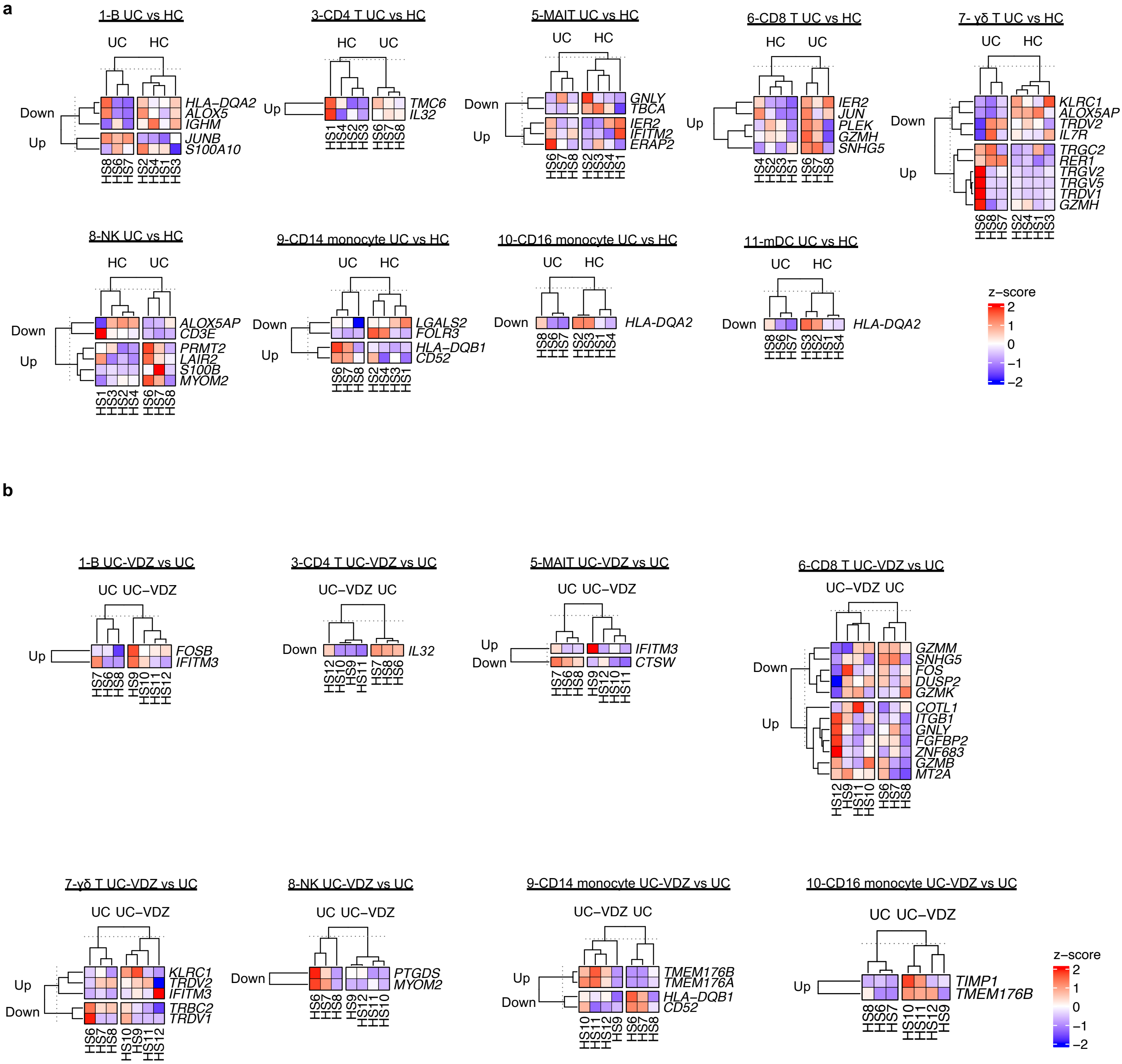

Supplementary Fig.2| PBL scRNA-seq DE gene analysis comparing UC versus HC and UC-VDZ versus UC. a,b, Heatmap of expression z-scores for scRNA-seq DE genes in the indicated PBL cell subsets with $\log_2$fc >2 or <-2 and and Bonferroni-corrected p-value<0.1 in (**a**) UC (Up/Down) relative to HC and (**b**) UC-VDZ (Up/Down) relative to UC identified by MAST analysis. Ribosomal and mitochondrial genes are not displayed; cell subsets without DE genes are not displayed.

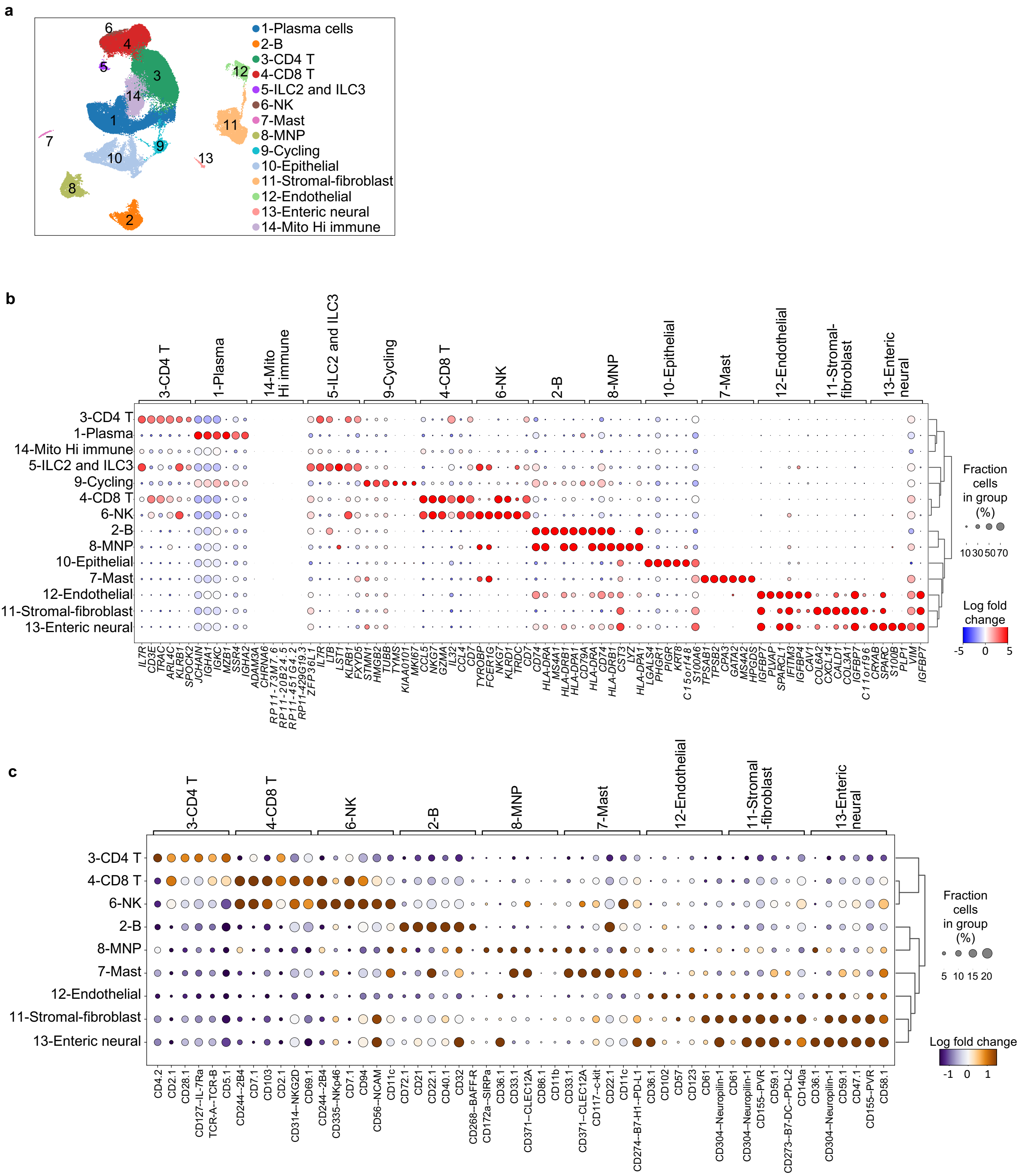

Supplementary Fig.3| Biopsy scRNA-seq and CITE-seq coarse cell subset annotations and landmark genes and proteins. a, UMAP visualization of scRNA-seq and CITE-seq for 93,900 cells from HC (n=4), UC (n=4), and UC-VDZ (n=4) patients highlighting coarse cell subset annotations. **b-c,** Dot plot representation of landmark (**b**) genes and (**c**) proteins expressed by indicated coarse cell subsets. Genes and proteins were ranked by Wilcoxon rank-sum. Cell subsets without landmark genes or proteins above selected minimum log fold change are not displayed.

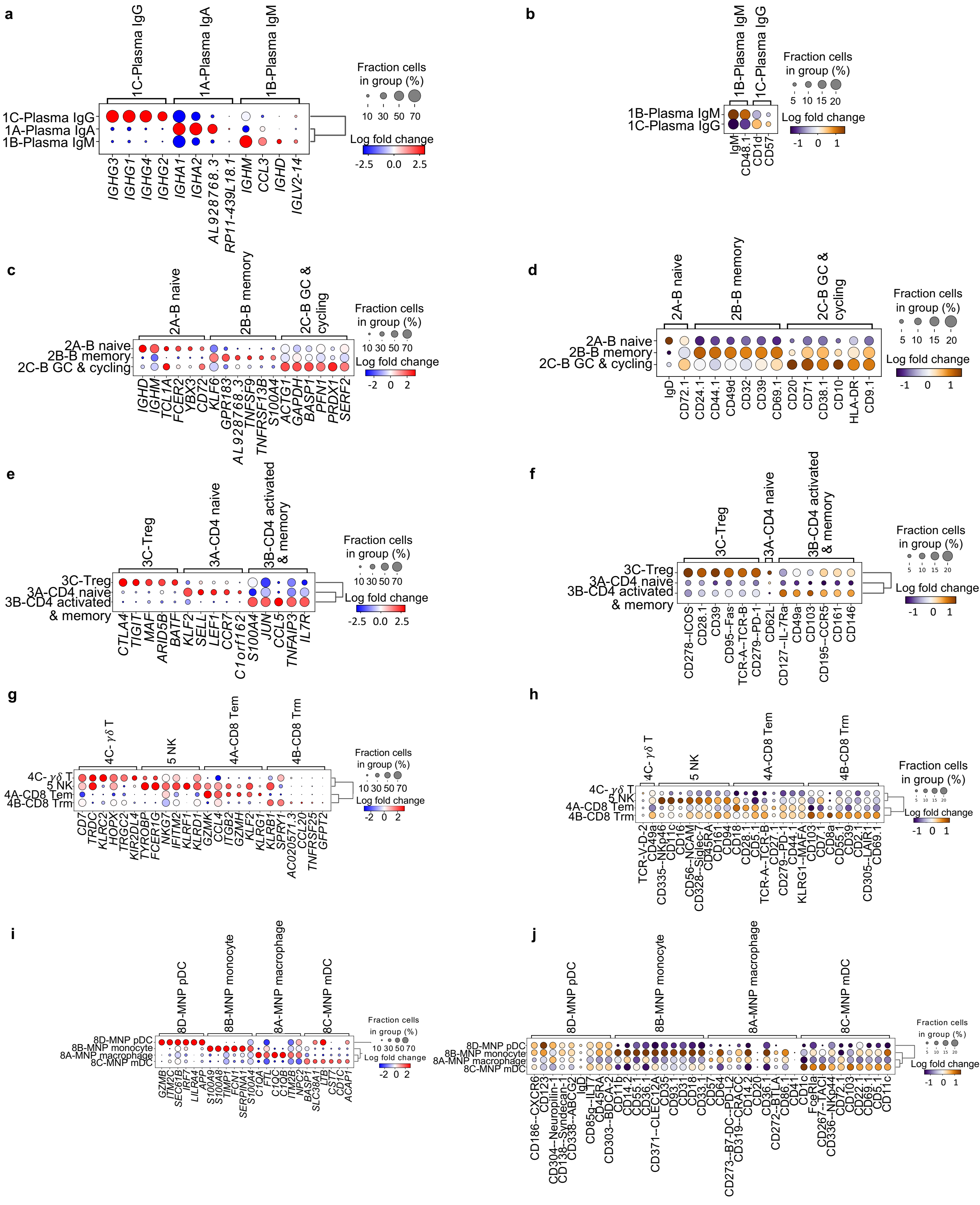

Supplementary Fig.4| Landmark genes and proteins associated with fine immune cell subsets in biopsy scRNA-seq and CITE-seq data. a-j, Dot plot representation of landmark (**a,c,e,g,i**) genes and (**b,d,f,h,j**) proteins expressed by indicated fine cell subsets. Genes and proteins were ranked by Wilcoxon rank-sum. Cell subsets without landmark genes or proteins above selected minimum log fold change are not displayed.

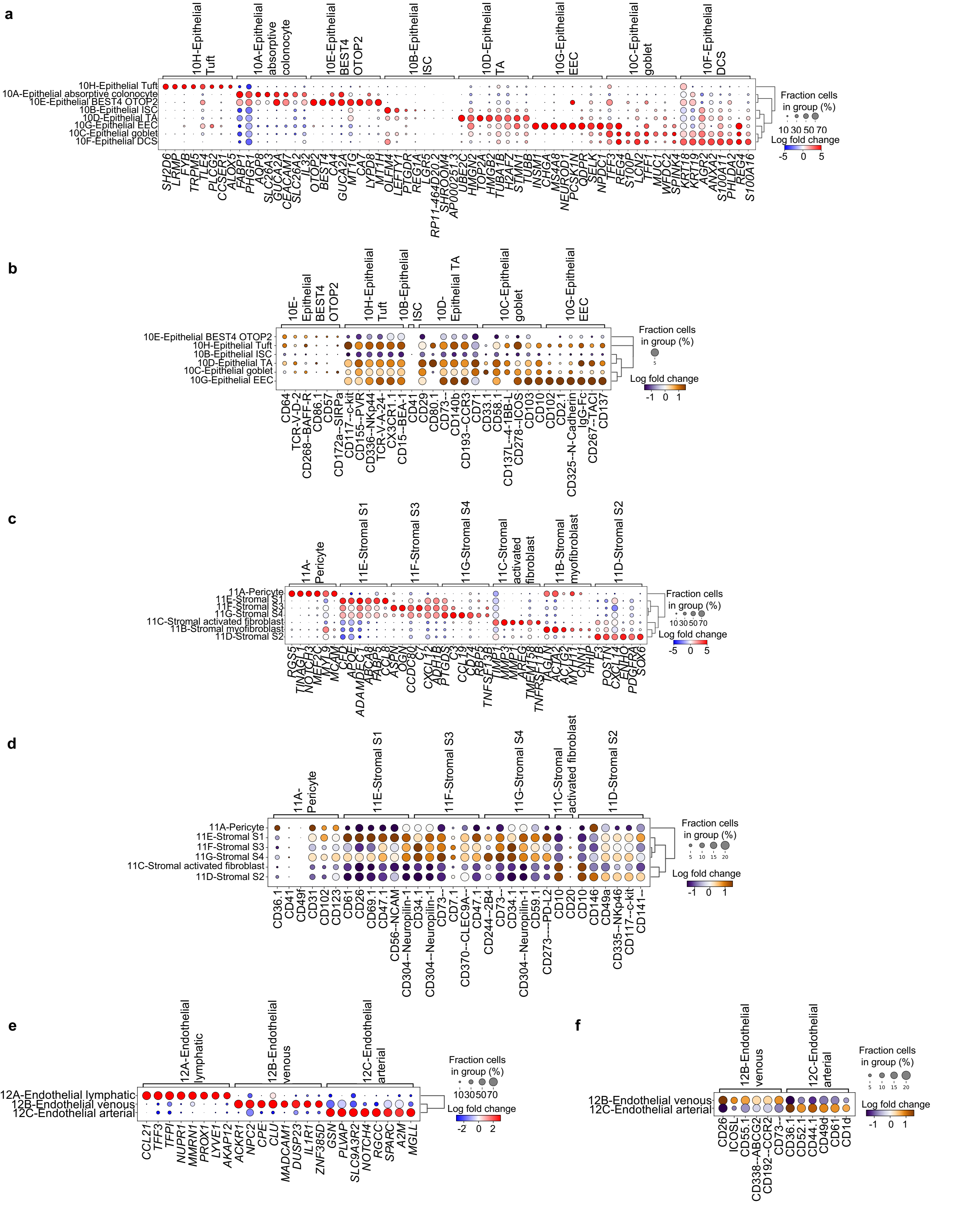

Supplementary Fig. 5| Landmark genes and proteins associated fine non-immune cell subsets in biopsy biopsy scRNA-seq and CITE-seq data. a-f, Dot plot representation of landmark (**a,c,e**) genes and (**b,d,f**) proteins expressed by indicated biopsy fine cell subsets. Genes and proteins were ranked by Wilcoxon rank-sum. Cell subsets without landmark genes or proteins above selected minimum log fold change are not displayed.

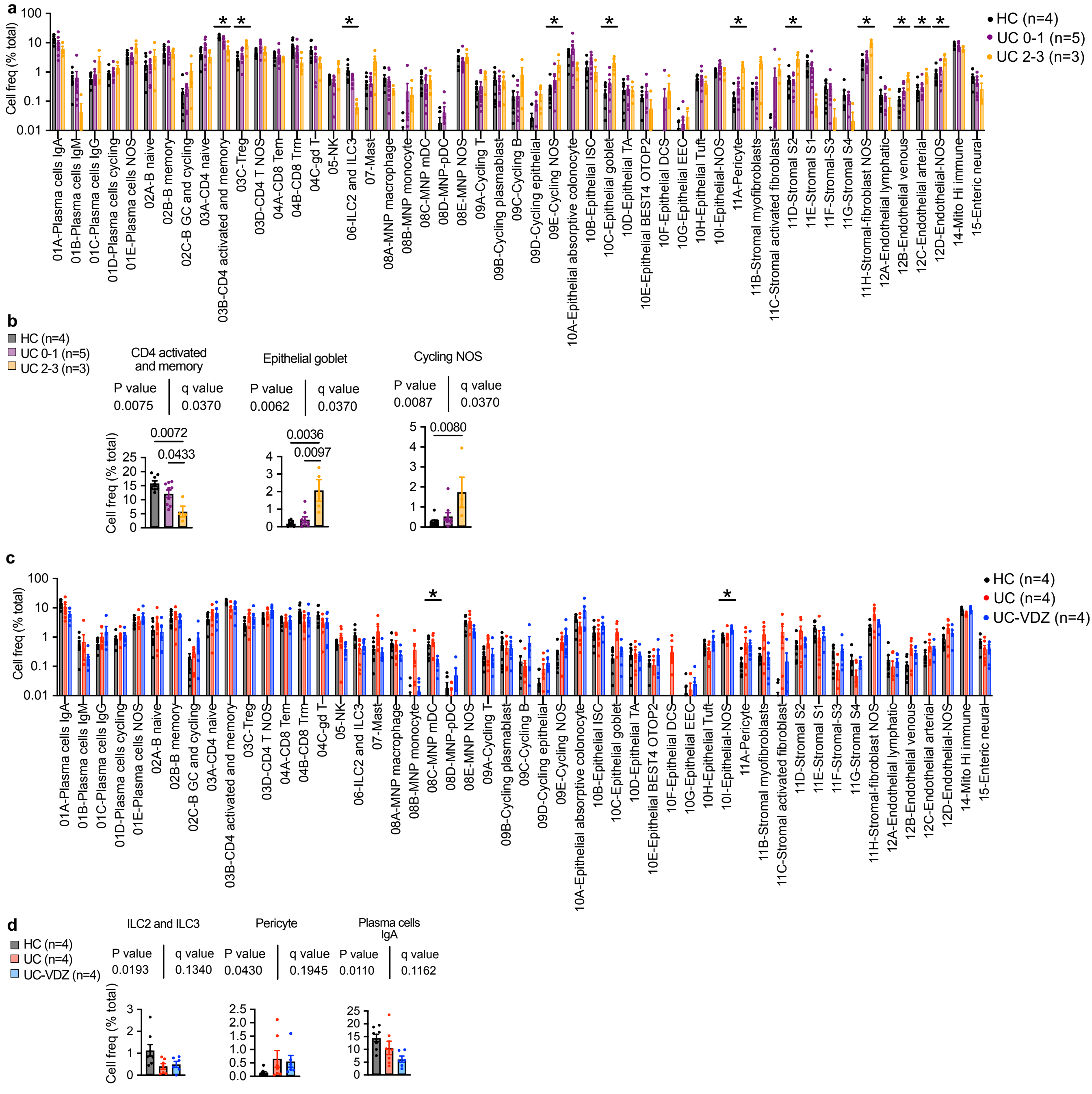

Supplementary Fig. 6| Biopsy CITE-seq cell subsets that correlated with inflammatory severity, disease status, and VDZ treatment. a-d, Cell frequency as a percent of total cells per study subject and fine cell annotation stratified by (**a,b**) endoscopic severity and (**c,d**) disease and treatment status (mean ± SEM; n=number of patients; each dot represents one patient sample). One-way ANOVA Kruskal-Wallis test was performed for each cell subset individually in (**a**, **c**), followed by global FDR correction of all p-values for all cell subsets, with q<0.1 threshold for discovery (*=q<0.1, only significant differences are indicated). A nested one-way ANOVA test was also performed on log transformed values treating biopsies as replicates with an unadjusted p<0.05 as an additional threshold for discovery (*). Select subsets are shown in (**b, d**) with exact p-value and q-value; NOS-"not otherwise specified".

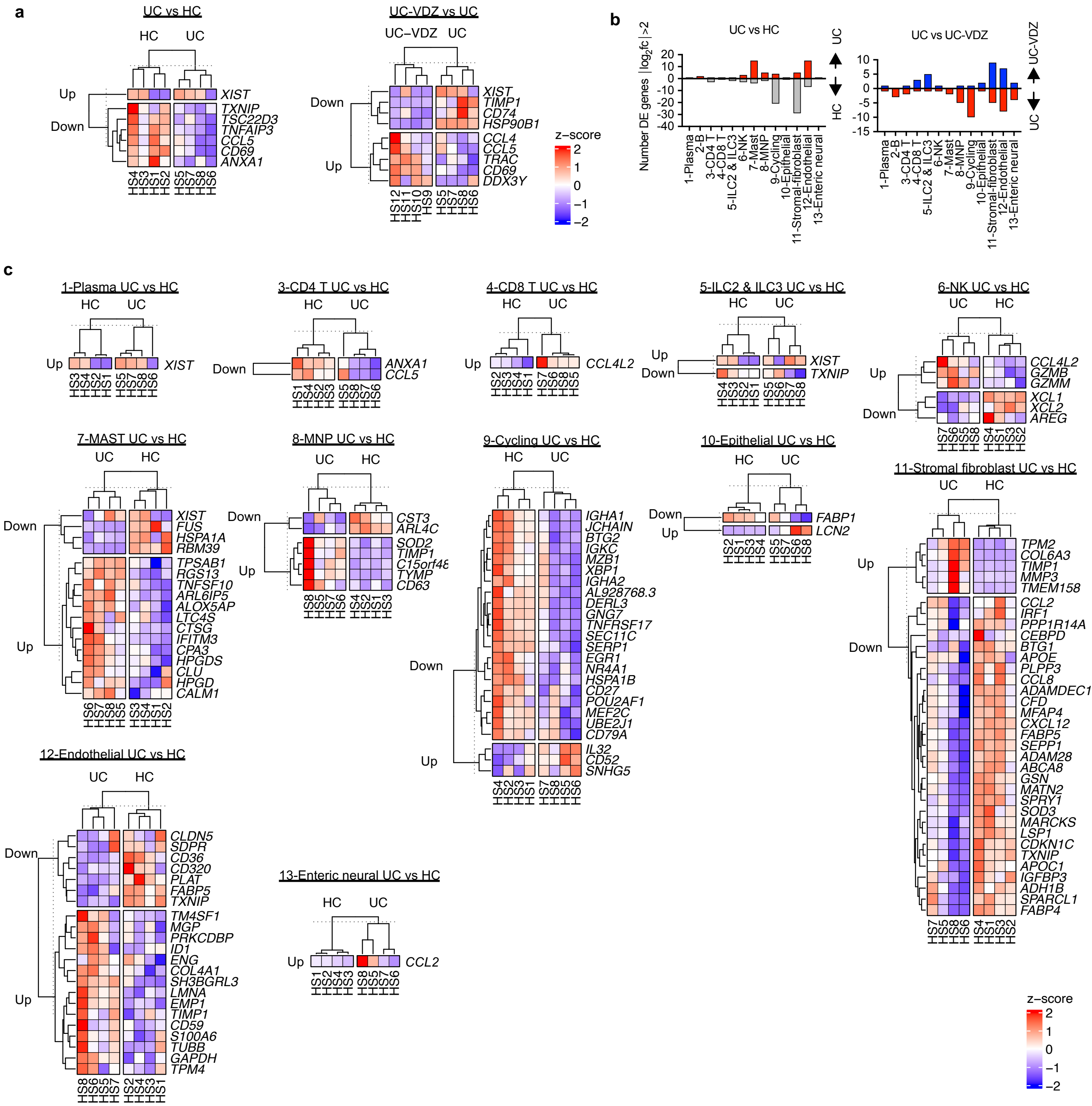

Supplementary Fig.7| Biopsy scRNA-seq DE genes in cell subsets comparing UC vs HC and UC-VDZ vs UC. a, Heatmap of expression z-scores for scRNA-seq DE genes for all biopsy cells, with $\log_2$fc >1 or <-1 and Bonferroni-corrected p-value<0.1, comparing UC (Up/Down) relative to HC and UC-VDZ (Up/Down) relative to UC identified by MAST analysis. **b,** Number of scRNA-seq DE genes in biopsy cell subsets with $\log_2$fc >2 or <-2 in UC versus HC and UC-VDZ versus UC identified by MAST analysis. **c,** Heatmap of expression z-scores for scRNA-seq DE genes for the indicated cell subsets with $\log_2$fc >2 or <-2 and Bonferroni-corrected p-value <0.1 in UC (Up/Down) relative to HC identified by MAST analysis. Ribosomal and mitochondrial genes are not displayed; cell subsets without DE genes are not displayed.

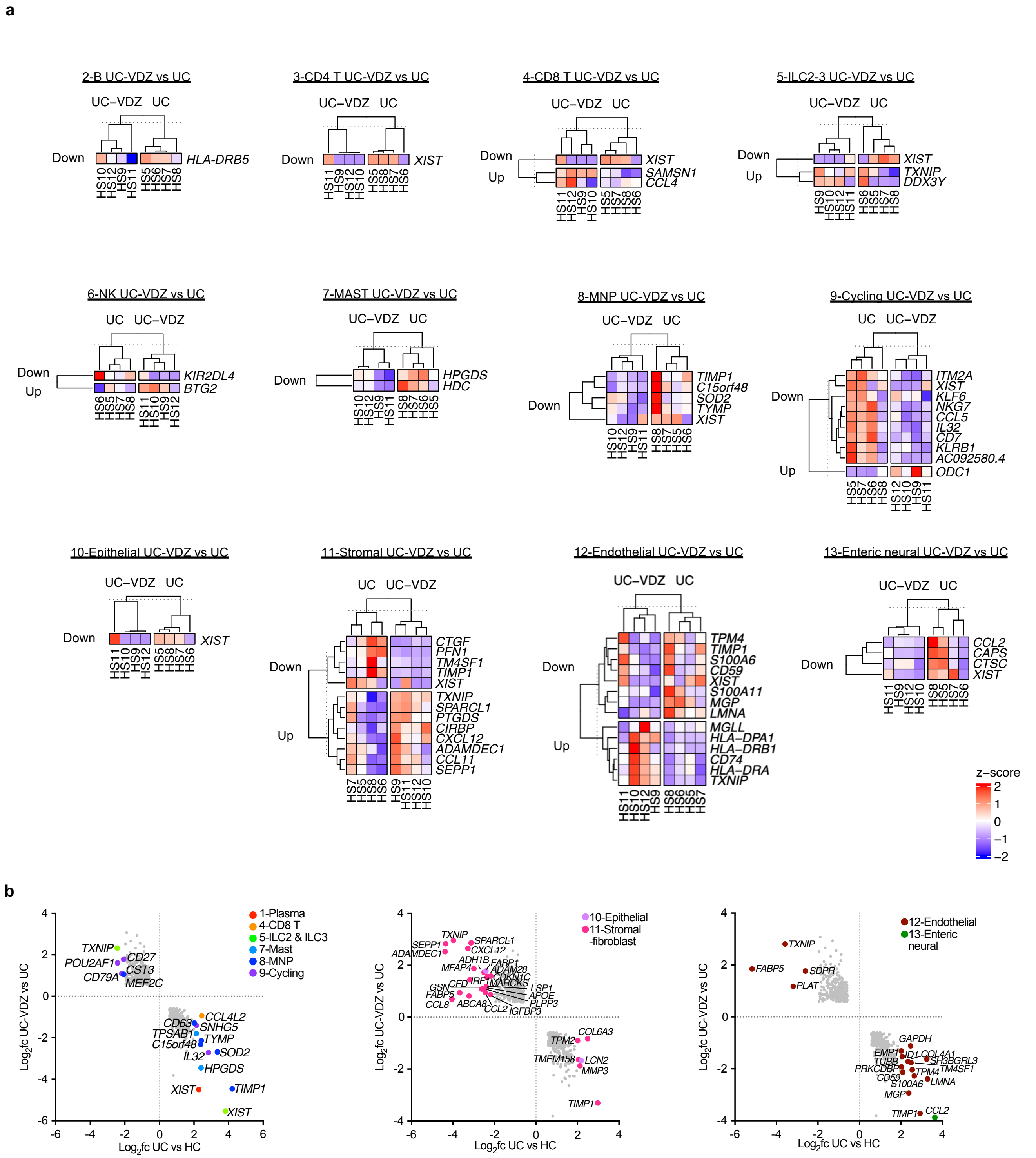

Supplementary Fig.8| Biopsy scRNA-seq DE genes in cell subsets comparing UC-VDZ vs UC and reciprocal expression analysis. a, Heatmap of expression z-scores for scRNA-seq DE genes for the indicated cell subsets with $\log_2$fc >2 or <-2 and Bonferroni-corrected p-value <0.1 in UC-VDZ (Up/Down) relative to UC identified by MAST analysis. **b,** scRNA-seq DE genes in biopsy coarse cell annotation subsets with $\log_2$fc >2 or <-2 in UC versus HC and an inverse $\log_2$fc for UC-VDZ versus UC with $\log_2$fc <-0.5 or >0.5 identified by MAST analysis, respectively. Ribosomal and mitochondrial genes are not displayed; cell subsets without DE genes are not displayed.

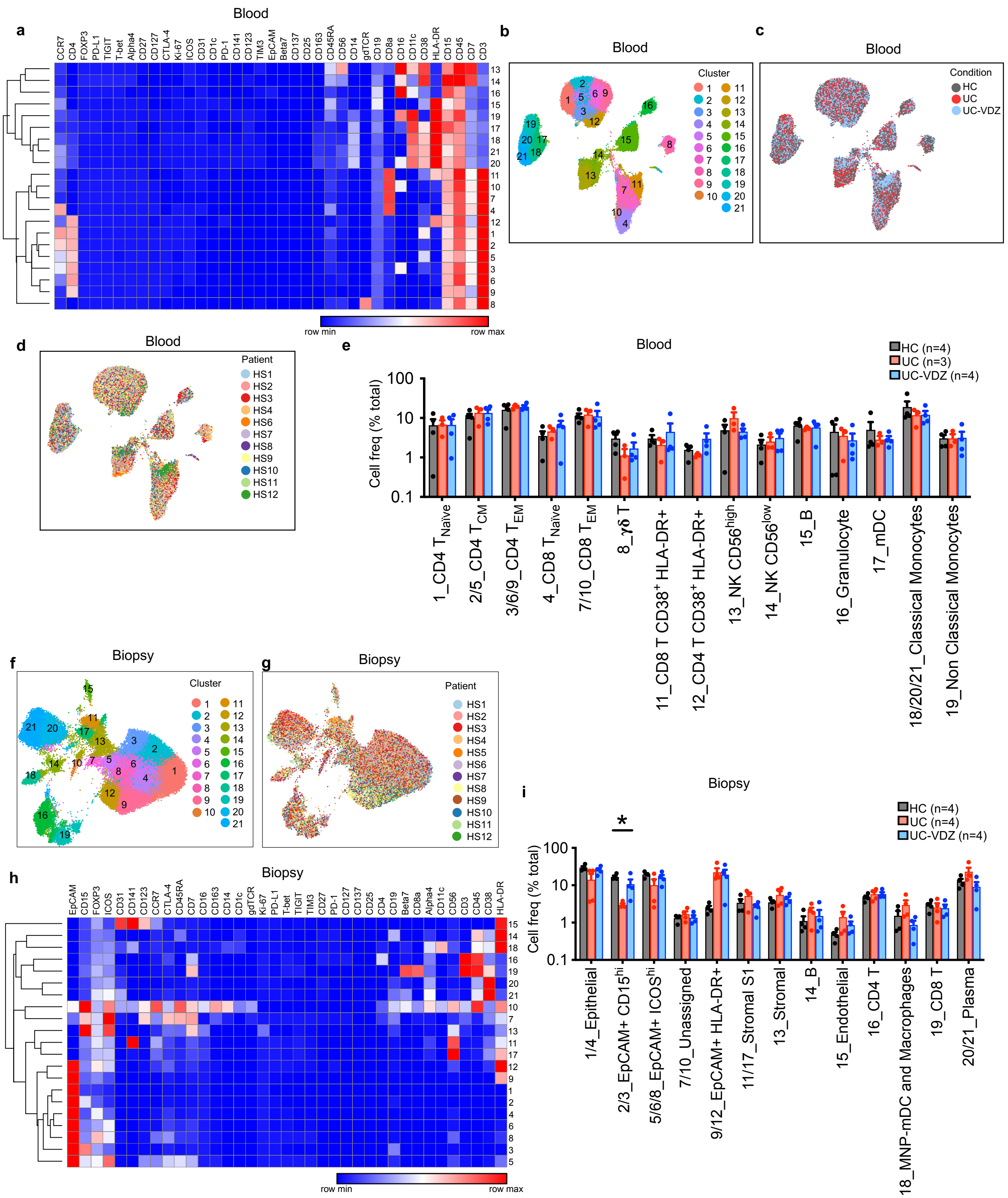

Supplementary Fig. 9| Heatmaps and total frequencies obtained from unsupervised analysis of CyTOF data from blood and biopsies. a,h, Heatmap visualization of mean intensity of each marker for the 21 identified clusters in blood and biopsies, respectively. **b-d,** UMAP visualization of the indicated samples (100,000 out of 1,390,156 live cell events displayed for blood) highlighting (**b**) FlowSOM identified clusters, (**c**) disease and treatment status, and (**d**) patient sample. **e,i,** Cell frequencies for each annotated cell subset per condition for (**e**) blood and (**i**) biopsy samples **(**mean ± SEM; n=number of patients; each dot represents one patient sample). **f,g,** UMAP visualization of the indicated samples (60,000 out of 684,249 live cell events displayed for biopsies) highlighting (**f**) FlowSOM identified clusters, and (**g**) patient sample. One-way ANOVA Kruskal-Wallis test was performed for each cell subset individually in panels (**e**) and (**i**), followed by global FDR correction of all p-values for all cell subsets, with q<0.1 threshold for discovery (*q<0.1, only significant differences are indicated).

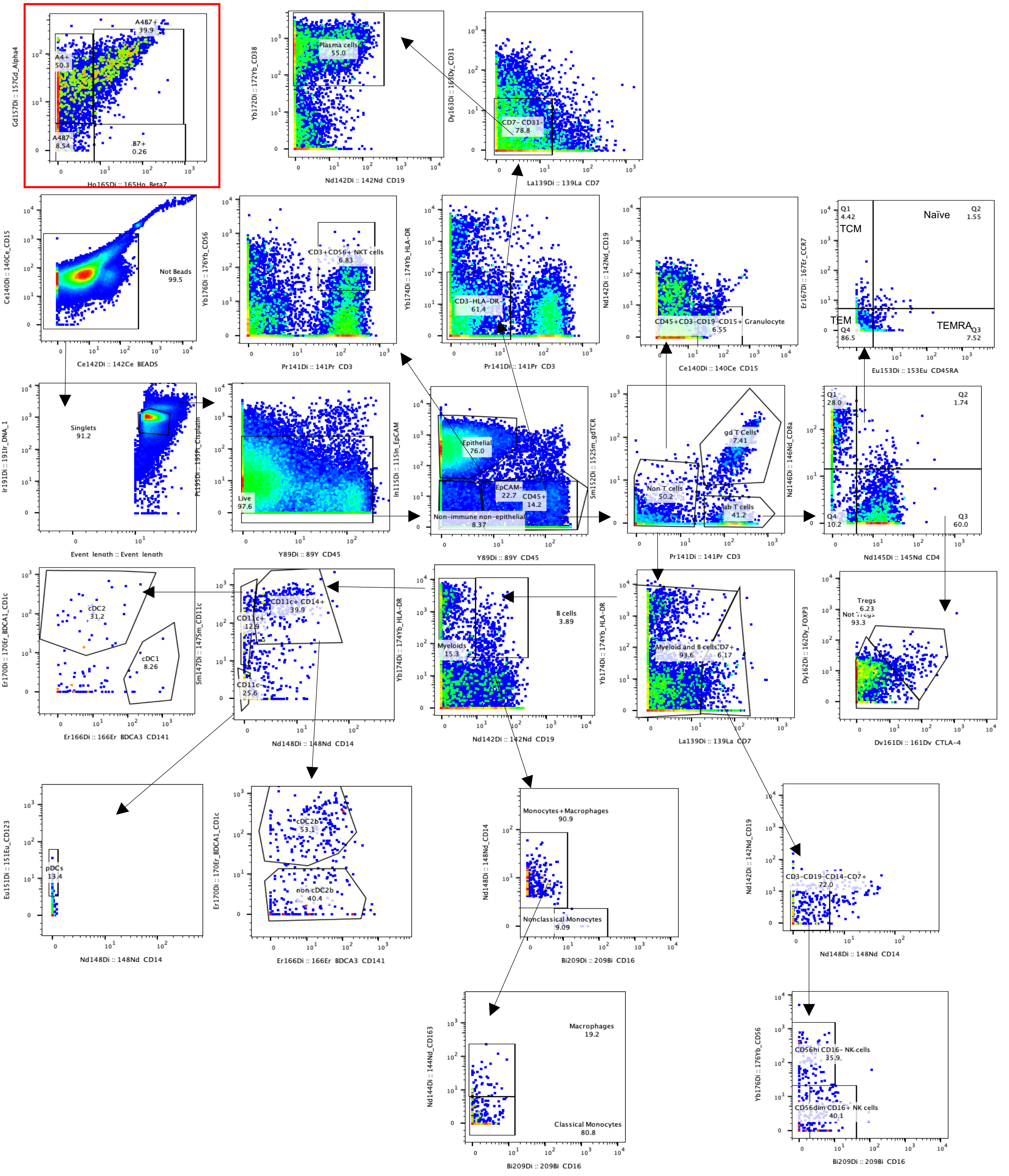

Supplementary Fig. 10| Representative supervised CyTOF gating scheme.

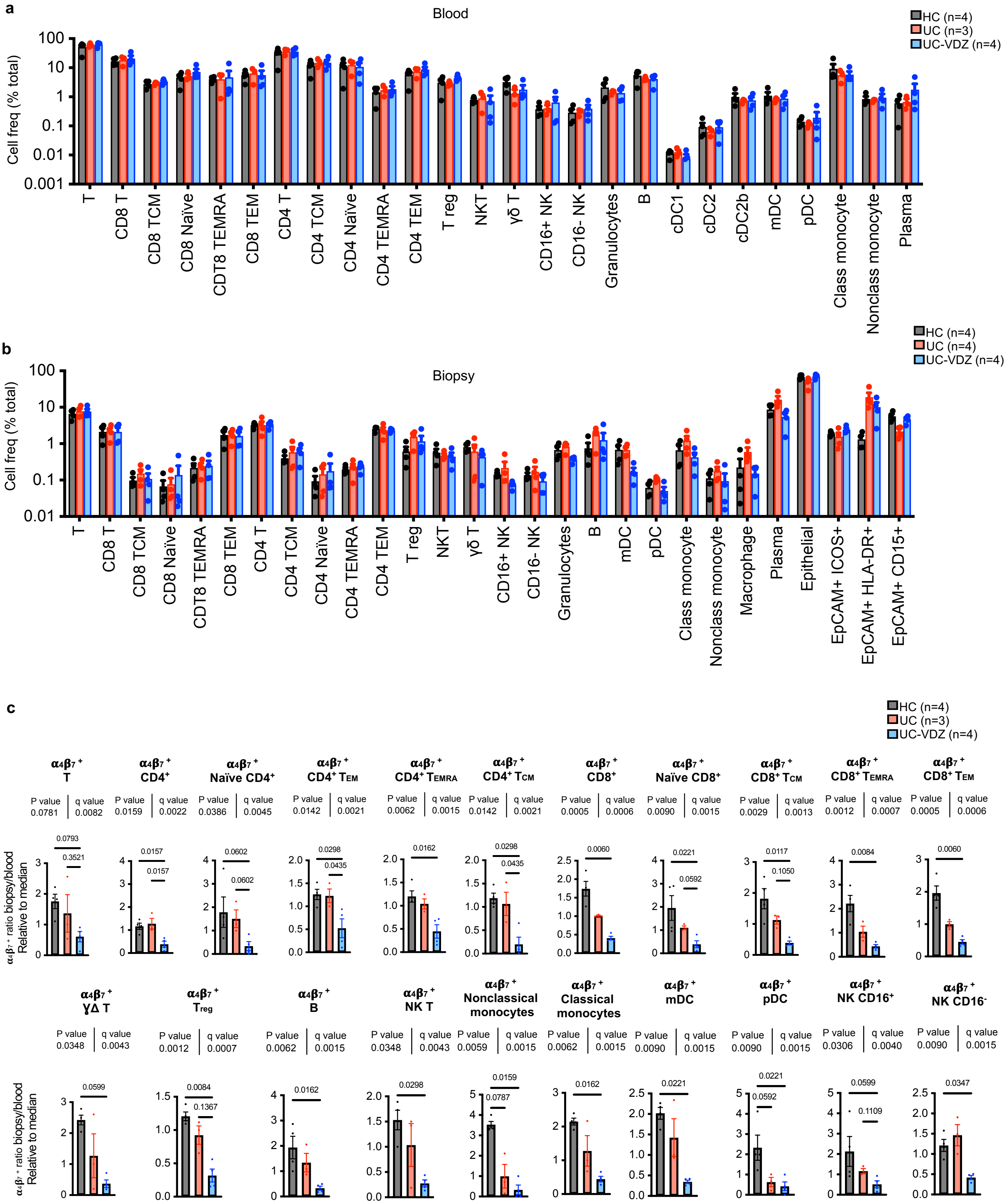

Supplementary Fig. 11| Total frequencies obtained from supervised analysis of CyTOF data from blood and biopsies and significant $\alpha_4\beta_7^+$ cell subsets. a,b, Cell frequencies for each annotated cell subset per condition in blood and biopsy, respectively. **c,** $\alpha_4\beta_7^+$ cell frequency in the biopsy versus blood for the indicated cell subsets relative to the median for each cell subset. For panels **a-b**, mean ± SEM; n=number of patients; each dot represents one patient sample; multiple one-way ANOVA Kruskal-Wallis test with FDR correction; q<0.1 threshold for discovery; individual subsets are shown in (**c**) with exact p-value and q-value; individual inter-column q-values are displayed only for cell subsets with overall FDR corrected q<0.01. Class monocyte-classical monocyte; Nonclass monocyte-nonclassical monocyte; mDC-cDC1,cDC2,cDC2b.

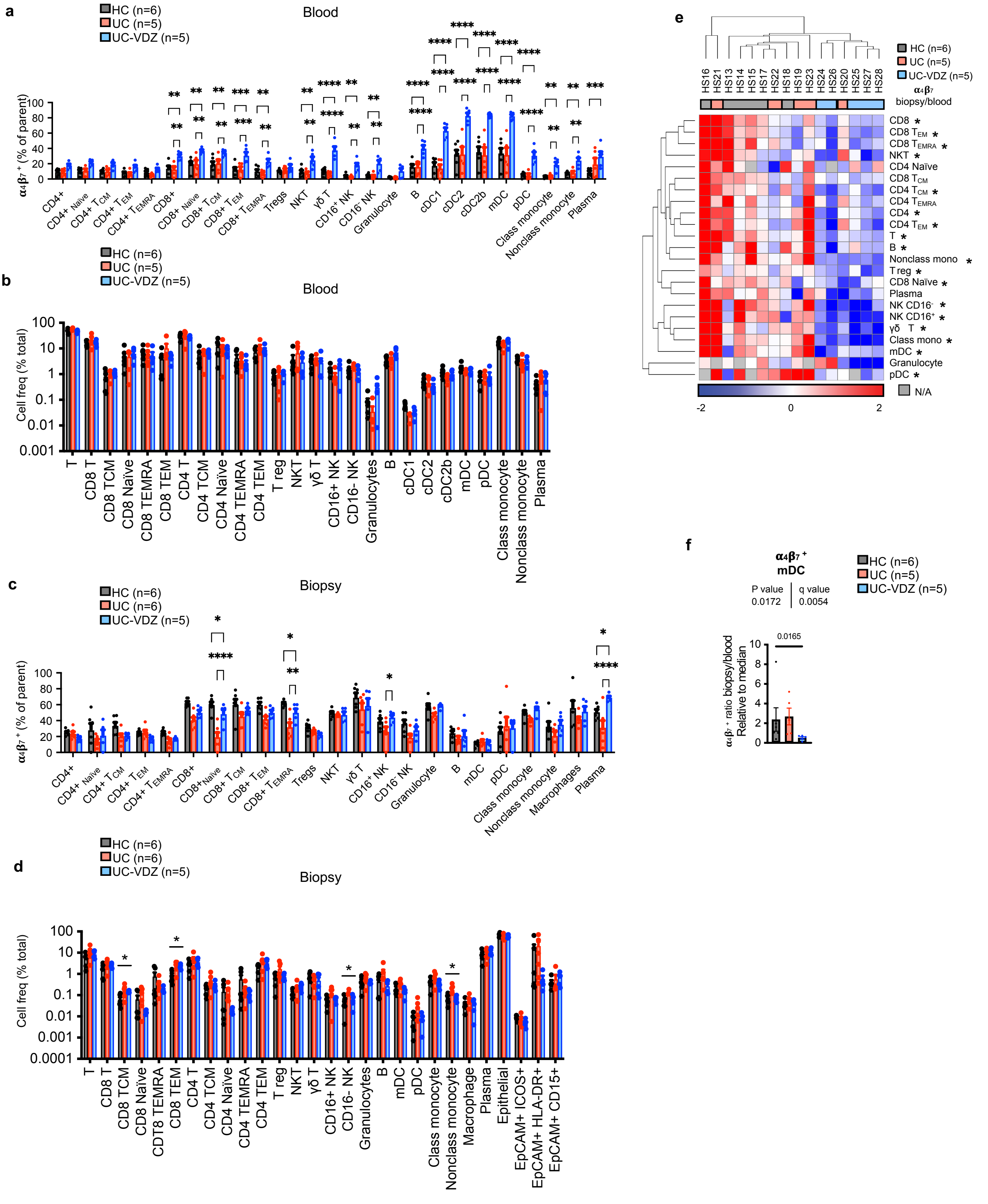

Supplementary Fig. 12| $\alpha_4\beta_7^+$ cell subsets and total frequencies obtained from supervised analysis of CyTOF data from blood and biopsies in a second case control study of patients in near or complete endoscopic remission (HS13-HS30). a,c, Percentage of $\alpha_4\beta_7^+$ cells in each defined cell subset per condition for blood and biopsy samples, respectively (mean ± SEM; n=number of patients; each dot represents one patient sample; two-way ANOVA comparing HC vs UC-VDZ and UC vs UC-VDZ with FDR correction; q<0.1 threshold for discovery; *q<0.05; **q<0.01; ***q<0.001; and ****q<0.0001; only significant differences are indicated. **b,d,** Cell frequencies for each annotated cell subset per condition in blood and biopsy, respectively (mean ± SEM; n=number of patients, multiple one-way ANOVA Kruskal-Wallis test with FDR correction; q<0.1 threshold for discovery). **e,** Heatmap of biopsy/blood ratio of $\alpha_4\beta_7^+$ cells for each cell subset by patient (hierarchically clustered by Euclidian distance, average linkage). **f,** $\alpha_4\beta_7^+$ cell frequency in the biopsy versus blood for mDC relative to the median (mean ± SEM; n=number of patients; each dot represents one patient sample).

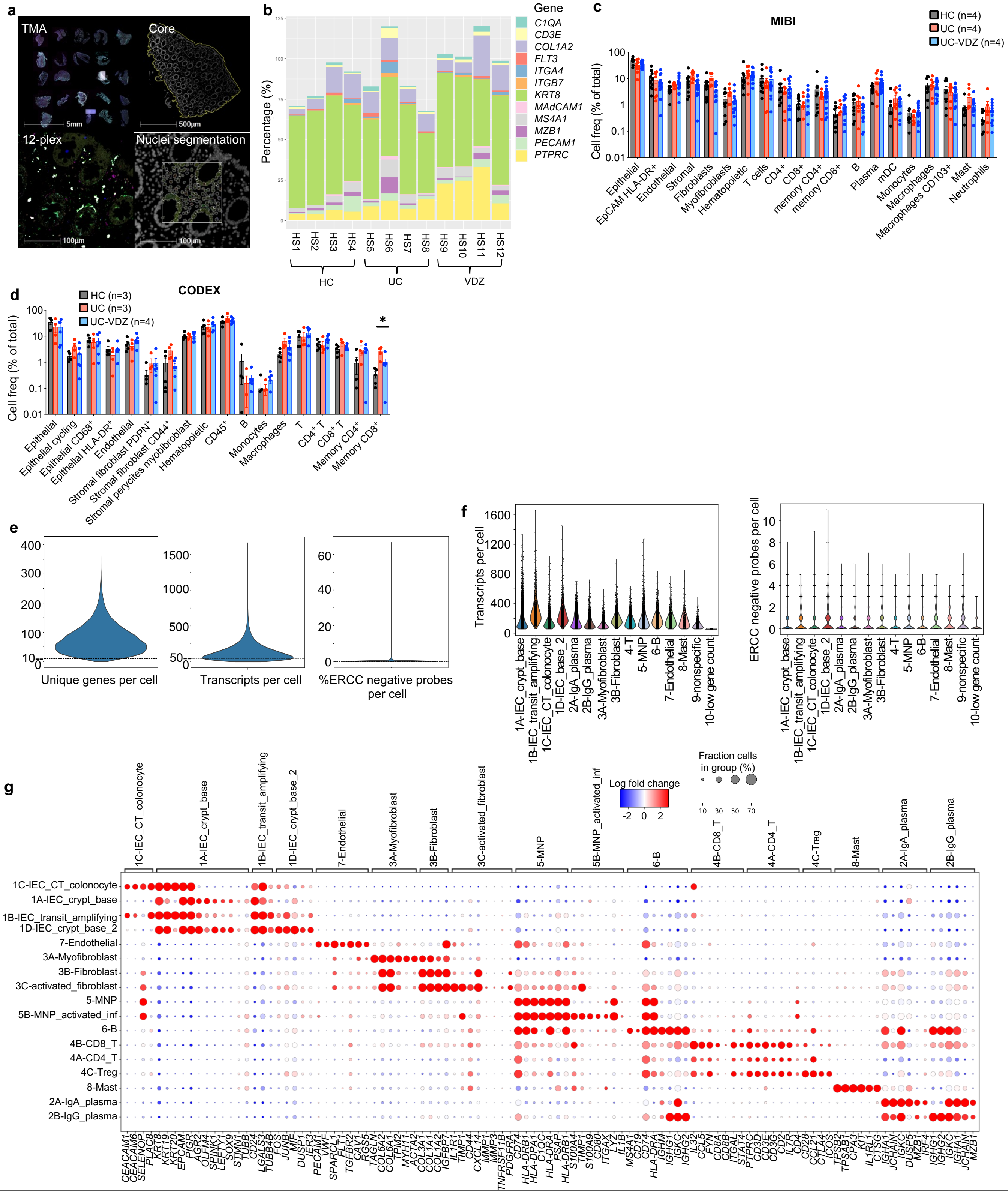

Supplementary Fig.13| Spatial transcriptomics and proteomics on FFPE tissue microarray (TMA). a, Image analysis workflow for 12-plex RNA-ISH assay using HALO, scan of the entire TMA (0.5X), with representative images (4X), representative overlay of the 12 markers (20X), and representative nuclei segmentation and localization of the 12 different markers (20X), which are representative of 19 cores. **b,** Stacked bar plots for cells expressing the indicated gene; values are greater than 100% due to cells co-expressing genes of interest. **c,d,** Cell frequencies as a percent of total for the indicated cell subsets identified by (**c**) MIBI and (**d**) CODEX; multiple one-way ANOVA Kruskal-Wallis test with FDR correction; q<0.1 threshold for discovery; *=q<0.1. **e,** Violin plots showing unique genes, transcripts, and %ERCC negative probes per cell. Cells with <10 unique genes or <50 transcripts per cell were excluded (dashed line). **f,** Transcripts and ERCC negative probes per cell, stratified by coarse annotation. **g,** Dot plot representation of curated landmark genes for annotated subsets. Genes ranked by Wilcoxon rank-sum. ERCC, External RNA Controls Consortium.

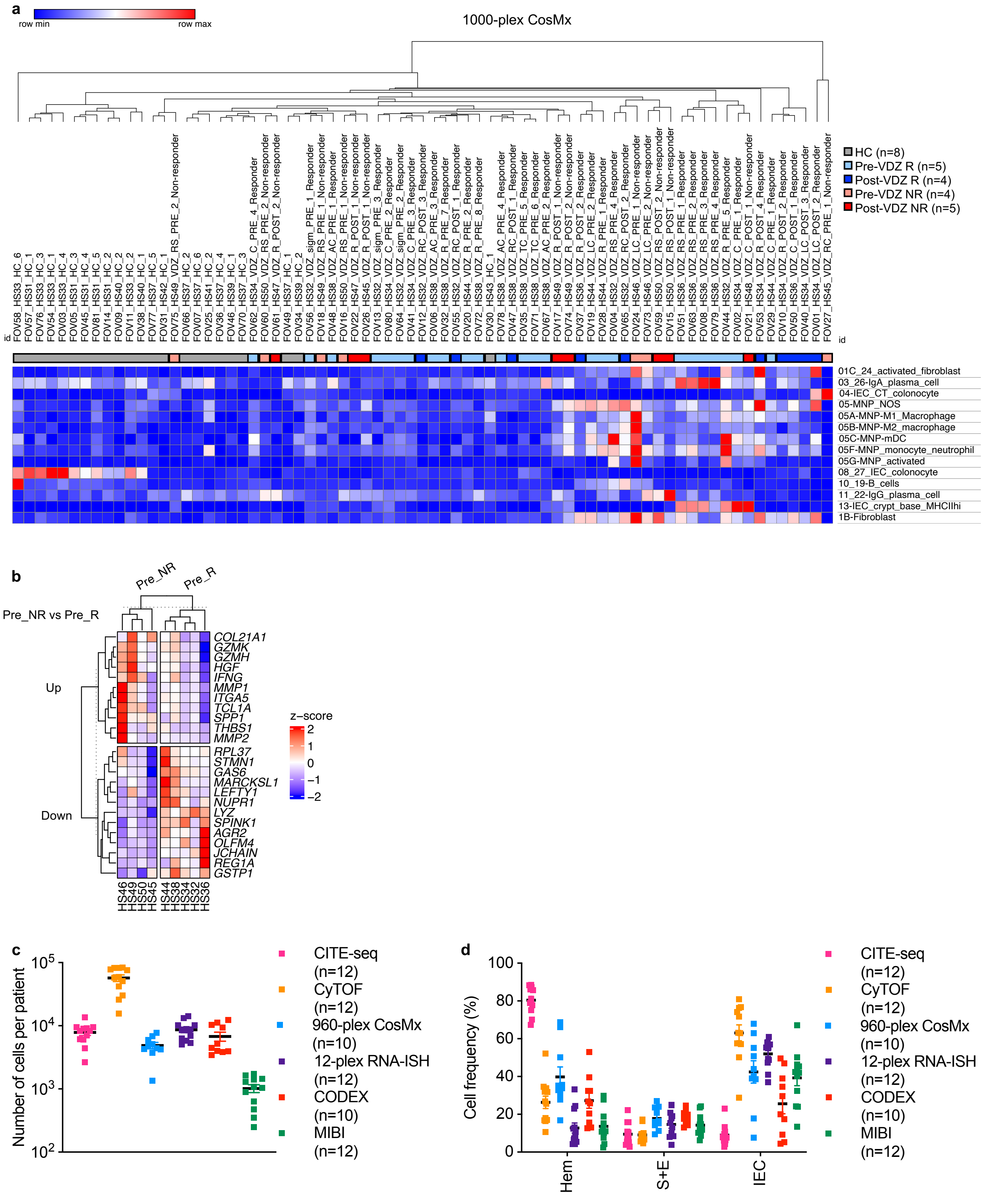

Supplementary Fig.14| CosMx spatial transcriptomic on TMA from retrospective longitudinal study and comparison of total cells analyzed per patient and cell frequency per tissue compartment among study subjects HS1-HS12. a, Heatmap of selected cell subsets by patient fov (hierarchically clustered by Euclidian distance, average linkage, columns color-coded by group). **b.** Heatmap of expression z-scores for the indicated genes in VDZ non-responders (Pre_NR) (Up/Down) relative to responders (Pre_R) before treatment identified by DESeq2. **c,** Total number of cells analyzed per patient using different multi-omic methods. **d,** Cell frequency per patient expressed as percent of total for the indicated cellular compartment among different multi-omic methods. For (**c, d**), mean ± SEM, n=number of patients, each dot represents one patient sample. Hem-hematopoietic; S+E-stromal + endothelial; IEC-intestinal epithelial cell.

Patient ID	Age (y)	Sex	Disease status	UC Medication	Disease duration (y)	Duration VDZ (mo)	Prior anti-TNF exposure	Montreal classification	Mayo Endoscopic subscore	Responder to VDZ
HS1	50-59	M	HC	None	n/a	n/a	No	n/a	0	n/a
HS2	40-49	M	HC	None	n/a	n/a	No	n/a	0	n/a
HS3	20-29	F	HC	None	n/a	n/a	No	n/a	0	n/a
HS4	50-59	F	HC	None	n/a	n/a	No	n/a	0	n/a
HS5	20-29	F	UC	5-ASA	15	n/a	No	E2	2	n/a
HS6	40-49	M	UC	5-ASA	25	n/a	Yes	E3	2	n/a
HS7	60-69	F	UC	5-ASA	7	n/a	No	E2	1	n/a
HS8	50-59	F	UC	5-ASA	5	n/a	No	E1	1	n/a
				Median	11 (7-18)					
HS9	50-59	M	UC	VDZ	25	8	Yes	E2	1	Yes
HS10	30-39	M	UC	VDZ	8	7	No	E2	1	Yes
HS11	20-29	F	UC	VDZ	8	64	Yes	E2	3	No
HS12	50-59	M	UC	VDZ	10	2	Yes	E3	1	Yes
					9 (8-14)					
p-value	ns	ns	p=0.0025	p<0.0001	ns	n/a	ns	ns	p=0.01	
HS13	20-29	M	HC	None	n/a	n/a	No	n/a	0	n/a
HS14	30-39	M	HC	None	n/a	n/a	No	n/a	0	n/a
HS15	20-29	F	HC	None	n/a	n/a	No	n/a	0	n/a
HS16	60-69	F	HC	None	n/a	n/a	No	n/a	0	n/a
HS17	50-59	F	HC	None	n/a	n/a	No	n/a	0	n/a
HS18	40-49	F	HC	None	n/a	n/a	No	n/a	0	n/a
HS19	40-49	M	UC	ADA	27	n/a	Yes	E2	0	n/a
HS20*	30-39	F	UC	ADA	20	n/a	Yes	E2	1-2	n/a
HS21	50-59	M	UC	IFX/MTX	6	n/a	Yes	E3	0	n/a
HS22	30-39	F	UC	5-ASA	6	n/a	No	E3	0	n/a
HS23	40-49	M	UC	IFX/AZA	10	n/a	Yes	E3	1	n/a
HS30	40-49	F	UC	ADA	2	n/a	Yes	E3	0	n/a
					8 (5-22)					
HS24	50-59	M	UC	VDZ	5.5	45	Yes	E2	0	Yes
HS25	60-69	M	UC	VDZ	37	5	No	E3	0	Yes
HS26	30-39	F	UC	VDZ	21	55	Yes	E3	0	Yes
HS27	30-39	F	UC	VDZ	1	6	No	E1	0	Yes
HS28	40-49	M	UC	VDZ	8	45	Yes	E1	0	Yes
					8 (3-29)					
p-value	ns	ns	p=0.0002	p<0.0001	ns	n/a	p=0.0329	ns	ns	
HS31 (HS16)	60-69	F	HC	None	n/a	n/a	No	n/a	0	n/a
HS33 (HS14)	30-39	M	HC	None	n/a	n/a	No	n/a	0	n/a
HS35 (HS15)	20-29	F	HC	None	n/a	n/a	No	n/a	0	n/a
HS37 (HS13)	20-29	M	HC	None	n/a	n/a	No	n/a	0	n/a
HS39	20-29	F	HC	None	n/a	n/a	No	n/a	0	n/a
HS40	60-69	M	HC	None	n/a	n/a	No	n/a	0	n/a
HS41	50-59	M	HC	None	n/a	n/a	No	n/a	0	n/a
HS42	40-49	M	HC	None	n/a	n/a	No	n/a	0	n/a
HS43	50-59	M	HC	None	n/a	n/a	No	n/a	0	n/a
HS32	20-29	F	UC	VDZ	2	4	No	E2	pre 2 ; post 0	Yes
HS34 (HS27)	30-39	F	UC	VDZ	1	6	No	E1	pre 2; post 0	Yes
HS36	40-49	M	UC	VDZ	3	2	Yes	E2	pre 3; post 0	Yes
HS38 (HS9)	50-59	M	UC	VDZ	25	7	Yes	E3	pre 2; post 0	Yes
HS44	30-39	F	UC	VDZ	2	2	No	E1	pre 1	Yes
HS45	40-49	M	UC	VDZ	14	19	Yes	E2	pre 1; post 3	No
HS46	40-49	n/a	UC	VDZ	21	2	Yes	E2	pre 3; post 3	No
HS47	30-39	M	UC	VDZ	11	5	Yes	E2	post 3	No
HS48	70-79	M	UC	VDZ	8	6	Yes	E3	pre 2; post 2	No
HS49	30-39	F	UC	VDZ	2	3	Yes	E2	pre 2; post 3	No
HS50	20-29	M	UC	VDZ	0	5	Yes	E2	pre 2; post 2	No
					3 (2-14)					
p-value	ns	ns	p<0.0001	p<0.0001	n/a	n/a	p=0.0010	ns	pre, ns; post, p=0.0016	

Supplementary Table 1. Baseline demographic and clinical data for study participants. Categorical variables were analyzed by Chi-square test and continuous variables were compared using one-way ANOVA with FDR correction or Mann-Whitney test where appropriate. ns, not significant; n/a, not applicable; pre, pre-VDZ tretament; post, post-VDZ treatment. *HS20 had a short segment of moderate proctitis, otherwise in endoscopic remission with no change in therapy. HS31, HS33, HS35, HS37, HS34, HS38 were additional samples from HS16, HS14, HS15, HS13, HS27, and HS9, respectively. ADA-adalimumab; IFX-infliximab; VDZ-vedolizumab; AZA-azathioprine; 5-ASA-5-aminosalicylic acid.

Patient ID	Disease status	Medication	Biopsy location-severity	Mayo Endoscopic subscore	Geboes grade	Biopsy sample ID	Blood scRNA-seq	Biopsy scRNA-seq CITE-seq	Blood CyTOF	Biopsy CyTOF	12-plex RNA-ISH	CODEX	MIBI	CosMx SMI 960-plex
HS1	HC	N/A	R-0	0	0.0	HS1-CLR1	Y	Y	Y	Y	Y (1-core)	N/A	N/A	Y (1-FOV)
			L-0		1.1	HS1-CLL1		Y			Y (1-core)	N/A	Y (2-FOV)	Y (2-FOV)
HS2	HC	N/A	R-0	0	1.1	HS2-CLR1	Y	Y	Y	Y	Y (1-core)	Y (1-core)	Y (3-FOV)	Y (1-FOV)
			L-0		1.1	HS2-CLL1		Y			Y (1-core)	Y (1-core)	Y (3-FOV)	Y (1-FOV)
HS3	HC	N/A	R-0	0	0.0	HS3-CLR1	Y	Y	Y	Y	N/A	Y (1-core)	N/A	Y (1-FOV)
			L-0		0.0	HS3-CLL1		Y			Y (1-core)	Y (1-core)	Y (1-FOV)	N/A
HS4	HC	N/A	R-0	0	0.0	HS4-CLR1	Y	Y	Y	Y	Y (1-core)	N/A	N/A	Y (1-FOV)
			L-0		0.0	HS4-CLL1		Y			Y (1-core)	Y (1-core)	Y (1-FOV)	Y (1-FOV)
HS5	UC	5-ASA	R-0	2	0.0	HS5-CLR1	N/A	Y	N/A	Y	Y (1-core)	Y (1-core)	Y (2-FOV)	Y (1-FOV)
			L-2		0.2	HS5-CLL1		Y			Y (1-core)	N/A	Y (1-FOV)	N/A
HS6	UC	5-ASA	R-2	2	2A.2	HS6-CLR1	Y	Y	Y	Y	Y (1-core)	N/A	Y (2-FOV)	N/A
			L-2		3.1	HS6-CLL1		Y			N/A	N/A	N/A	N/A
HS7	UC	5-ASA	R-1	1	1.1	HS7-CLR1	Y	Y	Y	Y	Y (1-core)	Y (1-core)	N/A	Y (1-FOV)
			L-0		0.0	HS7-CLL1		Y			Y (1-core)	Y (1-core)	Y (2-FOV)	Y (1-FOV)
HS8	UC	5-ASA	L-1	1	3.2	HS8-CLL1	Y	Y	Y	Y	Y (1-core)	Y (1-core)	Y (1-FOV)	Y (1-FOV)
HS9	UC	VDZ	R-0	1	0.0	HS9-CLR1	Y	Y	Y	Y	Y (1-core)	Y (1-core)	Y (3-FOV)	N/A
			L-1		0.0	HS9-CLL1		Y			Y (1-core)	Y (1-core)	Y (3-FOV)	N/A
HS10	UC	VDZ	R-0	1	1.1	HS10-CLR1	Y	Y	Y	Y	Y (1-core)	Y (1-core)	Y (1-FOV)	Y (1-FOV)
			L-1		0.1	HS10-CLL1		N/A			N/A	N/A	N/A	Y (1-FOV)
HS11	UC	VDZ	R-0	3	0.1	HS11-CLR1	Y	Y	Y	Y	N/A	N/A	N/A	Y (1-FOV)
			L-3		3.2	HS11-CLL1		Y			Y (1-core)	Y (1-core)	Y (1-FOV)	Y (1-FOV)
HS12	UC	VDZ	R-1	1	1.2	HS12-CLR1	Y	Y	Y	Y	Y (1-core)	Y (1-core)	Y (3-FOV)	N/A
			L-1		1.3	HS12-CLL1		N/A			Y (1-core)	Y (1-core)	Y (3-FOV)	Y (1-FOV)

Supplementary Table 2. Biospecimens analyzed by each multi-omics method. R, right colon; L, left colon; 5-ASA, 5-aminosalicylate; CLR1, right colon biopsy 1; CLL1, left colon biopsy; Y, yes; N/A not applicable. FOV-field of view

Assay type	Target	Ion channel / DNA barcode	Metal / DNA barcode ID	Clone/Probe	Concentration (µg/ml) /Dilution (ratio)	h5ad file symbol	Catalog #	Vendor
CITE-seq (TotalSeq)	CD103 (Integrin αE)	GACCTCATTGTGAAT	A0145	Ber-ACT8	proprietary	CD103	399907	BioLegend
	CD35	ACTTCCGTCGATCTT	A0167	E11	proprietary	CD35	399907	BioLegend
	CD25	TTTGTCCTGTACGCC	A0085	BC96	proprietary	CD25	399907	BioLegend
	CD62L	GTCCCTGCAACTTGA	A0147	DREG-56	proprietary	CD62L	399907	BioLegend
	CD3	CTCATTGTAACTCCT	A0034	UCHT1	proprietary	CD3	399907	BioLegend
	CD33	TAACTCAGGGCCTAT	A0052	P67.6	proprietary	CD33.1	399907	BioLegend
	CD4	TGTTCCCGCTCAACT	A0072	RPA-T4	proprietary	CD4.2	399907	BioLegend
	CD58 (LFA-3)	GTTCCTATGGACGAC	A0174	TS2/9	proprietary	CD58.1	399907	BioLegend
	CD8a	GCTGCGCTTTCCATT	A0080	RPA-T8	proprietary	CD8a	399907	BioLegend
	CD56	TCCTTTCCTGATAGG	A0047	5.1H11	proprietary	CD56--NCAM	399907	BioLegend
	CD11b	GACAAGTGATCTGCA	A0161	ICRF44	proprietary	CD11b	399907	BioLegend
	CD183 (CXCR3)	GCGATGGTAGATTAT	A0140	G025H7	proprietary	CD183--CXCR3	399907	BioLegend
	CD30	TCAGGGTGTGCTGTA	A0028	BY88	proprietary	CD30	399907	BioLegend
	CD336 (NKp44)	GGGCAATTAGCGAGT	A0802	P44-8	proprietary	CD336--NKp44	399907	BioLegend
	CD69	GTCTCTTGGCTTAAA	A0146	FN50	proprietary	CD69.1	399907	BioLegend
	CD335 (NKp46)	ACAATTTGAACAGCG	A0101	9E2	proprietary	CD335--NKp46	399907	BioLegend
	CD49f	TTCCGAGGATGATCT	A0070	GoH3	proprietary	CD49f	399907	BioLegend
	CD161	GTACGCAGTCCTTCT	A0149	HP-3G10	proprietary	CD161	399907	BioLegend
	CD45RO	CTCCGAATCATGTTG	A0087	UCHL1	proprietary	CD45RO	399907	BioLegend
	CD20	TTCTGGGTCCCTAGA	A0100	2H7	proprietary	CD20	399907	BioLegend
	KLRG1 (MAFA)	CTTATTTCCTGCCCT	A0153	SA231A2	proprietary	KLRG1--MAFA	399907	BioLegend
	CD19	CTGGGCAATTACTCG	A0050	HIB19	proprietary	CD19.1	399907	BioLegend
	CD32	GCTTCCGAATTACCG	A0142	FUN-2	proprietary	CD32	399907	BioLegend
	CD276 (B7-H3)	GACTGGGAGGGTATT	A0010	DCN.70	proprietary	CD276--B7-H3	399907	BioLegend
	CD279 (PD-1)	ACAGCGCCGTATTTA	A0088	EH12.2H7	proprietary	CD279--PD-1	399907	BioLegend
	CD197 (CCR7)	AGTTCAGTCAACCGA	A0148	G043H7	proprietary	CD197--CCR7	399907	BioLegend
	CD252 (OX40L)	TTTAGTGATCCGACT	A0021	11C3.1	proprietary	N/A	399907	BioLegend
	CD223 (LAG-3)	CATTTGTCTGCCGGT	A0152	11C3C65	proprietary	CD223--LAG-3	399907	BioLegend
	HLA-DR	AATAGCGAGCAAGTA	A0159	L243	proprietary	HLA-DR	399907	BioLegend
	CD45RA	TCAATCCTTCCGCTT	A0063	HI100	proprietary	CD45RA	399907	BioLegend
	CD196 (CCR6)	GATCCCTTTGTCACT	A0143	G034E3	proprietary	CD196--CCR6	399907	BioLegend
	CD2	TACGATTTGTCAGGG	A0367	TS1/8	proprietary	CD2.1	399907	BioLegend
	CD326 (Ep-CAM)	TTCCGAGCAAGTATC	A0123	9C4	proprietary	CD326--EpCAM	399907	BioLegend
	CD169 (Sialoadhesin, Siglec-1)	TACTCAGCGTGTTTG	A0206	7-239	proprietary	CD169--Sialoadhesin--Siglec-1	399907	BioLegend
	CD41	ACGTTGTGGCCTTGT	A0353	HIP8	proprietary	CD41	399907	BioLegend
	CD49b	GCTTTCTTCAGTATG	A0371	P1E6-C5	proprietary	CD49b	399907	BioLegend
	CD303 (BDCA-2)	GAGATGTCCGAATTT	A0370	201A	proprietary	CD303--BDCA-2	399907	BioLegend
	CD29	GTATTCCCTCAGTCA	A0369	TS2/16	proprietary	CD29	399907	BioLegend
	CD184 (CXCR4)	TCAGGTCCTTTCAAC	A0366	12G5	proprietary	CD184--CXCR4	399907	BioLegend
	CD98	GCACCAACAGCCATT	A0374	MEM-108	proprietary	CD98	399907	BioLegend
	CD124 (IL-4Rα)	CCGTCCTGATAGATG	A0363	G077F6	proprietary	N/A	399907	BioLegend
	CD135 (Flt-3/Flk-2)	CAGTAGATGGAGCAT	A0351	BV10A4H2	proprietary	CD135	399907	BioLegend
	CD370 (CLEC9A/DNGR1)	CTGCATTTCAGTAAG	A0207	8F9	proprietary	CD370--CLEC9A--DNGR1	399907	BioLegend
	CD21	AACCTAGTAGTTCGG	A0181	Bu32	proprietary	CD21	399907	BioLegend
	CD39	TTACCTGGTATCCGT	A0176	A1	proprietary	CD39	399907	BioLegend
	CD141 (Thrombomodulin)	GGATAACCGCGCTTT	A0163	M80	proprietary	CD141--Thrombomodulin	399907	BioLegend
	CD64	AAGTATGCCCTACGA	A0162	10.1	proprietary	CD64	399907	BioLegend
	CD152 (CTLA-4)	ATGGTTCACGTAATC	A0151	BNI3	proprietary	CD152	399907	BioLegend
	CD61	AGGTTGGAGTAGACT	A0372	VI-PL2	proprietary	CD61	399907	BioLegend
	CD163	GCTTCTCCTTCCTTA	A0358	GHI/61	proprietary	CD163.1	399907	BioLegend
	CD357 (GITR)	ACCTTTCGACACTCG	A0360	108-17	proprietary	CD357--GITR	399907	BioLegend
	CD137 (4-1BB)	CAGTAAGTTCGGGAC	A0355	4B4-1	proprietary	CD137	399907	BioLegend
	CD81 (TAPA-1)	GTATCCTTCCTTGGC	A0373	5A6	proprietary	CD81--TAPA-1	399907	BioLegend
	CD366 (Tim-3)	TGTCCTACCCAACTT	A0169	F38-2E2	proprietary	CD366--Tim-3	399907	BioLegend
	CD57 Recombinant	AACTCCCTATGGAGG	A0168	QA17A04	proprietary	CD57	399907	BioLegend
	CD278 (ICOS)	CGCGCACCCATTAAA	A0171	C398.4A	proprietary	CD278--ICOS	399907	BioLegend
	CD95 (Fas)	CCAGCTCATTAGAGC	A0156	DX2	proprietary	CD95--Fas	399907	BioLegend
	CD80	ACGAATCAATCTGTG	A0005	2D10	proprietary	CD80.1	399907	BioLegend
	CD138 (Syndecan-1)	ACTCTTTCGTTTACG	A0055	MI15	proprietary	CD138--Syndecan-1	399907	BioLegend
	CD70	CGCGAACATAAGAAG	A0027	113-16	proprietary	CD70.1	399907	BioLegend
	CD269 (BCMA)	CAGATGATCCACCAT	A0056	19F2	proprietary	CD269--BCMA	399907	BioLegend
	IgM	TAGCGAGCCCGTATA	A0136	MHM-88	proprietary	IgM	399907	BioLegend
	CD59	AATTAGCCGTCGAGA	A0361	p282 (H19)	proprietary	CD59.1	399907	BioLegend
	CD194 (CCR4)	AGCTTACCTGCACGA	A0071	L291H4	proprietary	CD194--CCR4	399907	BioLegend
	CD275 (B7-H2, ICOSL)	GTGCATTCAACAGTA	A0009	2D3	proprietary	CD275--B7-H2--ICOSL	399907	BioLegend
	CD314 (NKG2D)	CGTGTTTGTTCCTCA	A0165	1D11	proprietary	CD314--NKG2D	399907	BioLegend
	CD86	GTCTTTGTCAGTGCA	A0006	IT2.2	proprietary	CD86.1	399907	BioLegend
	Galectin-9	ACTCACTGGAGTCTC	A0016	9M1-3	proprietary	N/A	399907	BioLegend
	CD195 (CCR5)	CCAAAGTAAGAGCCA	A0141	J418F1	proprietary	CD195--CCR5	399907	BioLegend
	CD1c	GAGCTACTTCACTCG	A0160	L161	proprietary	CD1c	399907	BioLegend
	CD28	TGAGAACGACCCTAA	A0386	CD28.2	proprietary	CD28.1	399907	BioLegend
	TSLPR (TSLP-R)	CAGTCCTCTCTGTCA	A0387	1D3	proprietary	TSLPR	399907	BioLegend
	CD38	TGTACCCGCTTGTGA	A0389	HIT2	proprietary	CD38.1	399907	BioLegend
	CD155 (PVR)	ATCACATCGTTGCCA	A0023	SKII.4	proprietary	CD155--PVR	399907	BioLegend
	CD270 (HVEM, TR2)	TGATAGAAACAGACC	A0020	122	proprietary	CD270--HVEM--TR2	399907	BioLegend
	CD178 (Fas-L)	CCGGTCCTCTGTATT	A0177	NOK-1	proprietary	CD178--FasL	399907	BioLegend
	CD127 (IL-7Rα)	GTGTGTTGTCCTATG	A0390	A019D5	proprietary	CD127--IL-7Ra	399907	BioLegend
	CD7	TGGATTCCCGGACTT	A0066	CD7-6B7	proprietary	CD7.1	399907	BioLegend
	CD117 (c-kit)	AGACTAATAGCTGAC	A0061	104D2	proprietary	CD117--c-kit	399907	BioLegend
	CD10	CAGCCATTCATTAGG	A0062	HI10a	proprietary	CD10	399907	BioLegend
	CD40	CTCAGATGGAGTATG	A0031	5C3	proprietary	CD40.1	399907	BioLegend
	CD48	CTACGACGTAGAAGA	A0029	BJ40	proprietary	CD48.1	399907	BioLegend
	CD154	GCTAGATAGATGCAA	A0032	24-31	proprietary	CD154	399907	BioLegend
	CD47	GCATTCTGTCACCTA	A0026	CC2C6	proprietary	CD47.1	399907	BioLegend
	CD112 (Nectin-2)	AACCTTCCGTCTAAG	A0024	TX31	proprietary	CD112--Nectin-2	399907	BioLegend
	CD52	CTTTGTACGAGCAAA	A0033	HI186	proprietary	CD52.1	399907	BioLegend
	CD71	CCGTGTTCCTCATTA	A0394	CY1G4	proprietary	CD71	399907	BioLegend
	CD22	GGGTTGTTGTCTTTG	A0393	S-HCL-1	proprietary	CD22.1	399907	BioLegend
	CD15 (SSEA-1)	TCACCAGTACCTAGT	A0392	W6D3	proprietary	CD15--BEA-1	399907	BioLegend

XCR1	AAGACGCATGTCAAC	A0208	S15046E	proprietary	XCR1.1	399907	BioLegend
CD26	GGTGGCTAGATAATG	A0396	BA5b	proprietary	CD26	399907	BioLegend
B7-H4	TGTATGTCTGCCTTG	A0395	MIH43	proprietary	B7-H4	399907	BioLegend
CD31	ACCTTTATGCCACGG	A0124	WM59	proprietary	CD31	399907	BioLegend
CD14	TCTCAGACCTCCGTA	A0081	M5E2	proprietary	CD14.2	399907	BioLegend
TIGIT (VSTM3)	TTGCTTACCGCCAGA	A0089	A15153G	proprietary	TIGIT--VSTM3	399907	BioLegend
CD294 (CRTH2)	TGTTTACGAGAGCCC	A0102	BM16	proprietary	CD294--CRTH2	399907	BioLegend
CD45	TCCCTTGCGATTTAC	A0048	2D1	proprietary	CD45	399907	BioLegend
CD34	GCAGAAATCTCCCTT	A0054	581	proprietary	CD34.1	399907	BioLegend
CD244 (2B4)	TCGCTTGGATGGTAG	A0189	C1.7	proprietary	CD244--2B4	399907	BioLegend
CD185 (CXCR5)	AATTCAACCGTCGCC	A0144	J252D4	proprietary	CD185--CXCR5	399907	BioLegend
CD18	TATTGGGACACTTCT	A0385	TS1/18	proprietary	CD18	399907	BioLegend
CD119 (IFN-? R ? chain)	TGTGTATTCCCTTGT	A0219	GIR-208	proprietary	CD119--IFN-G-R-A-chain	399907	BioLegend
CD134 (OX40)	AACCCACCGTTGTTA	A0158	Ber-ACT35 (ACT35)	proprietary	CD134--OX40	399907	BioLegend
CD24	AGATTCCTTCGTGTT	A0180	ML5	proprietary	CD24.1	399907	BioLegend
TCR γ/δ	CTTCCGATTCATTCA	A0139	B1	proprietary	TCR-G--TCR-D	399907	BioLegend
CD115 (CSF-1R)	AATCACGGTCCTTGT	A0398	9-4D2-1E4	proprietary	CD115--CSF-1R	399907	BioLegend
CD1d	TCGAGTCGCTTATCA	A0164	51.1	proprietary	CD1d	399907	BioLegend
CD192 (CCR2)	GAGTTCCCTTACCTG	A0242	K036C2	proprietary	CD192--CCR2	399907	BioLegend
CD105	ATCGTCGAGAGCTAG	A0068	43A3	proprietary	CD105	399907	BioLegend
CD144 (VE-Cadherin)	TCCACTCATTCTGTA	A0400	BV9	proprietary	CD144--VE-cadherin	399907	BioLegend
CD193 (CCR3)	ACCAATCCTTTCGTC	A0397	5E8	proprietary	CD193--CCR3	399907	BioLegend
CD55	GCTCATTACCCATTA	A0383	JS11	proprietary	CD55.1	399907	BioLegend
CD79b (Igβ)	ATTCTTCAACCGAAG	A0187	CB3-1	proprietary	CD79b--Ig-B	399907	BioLegend
CD123	CTTCACTCTGTCAGG	A0064	6H6	proprietary	CD123	399907	BioLegend
CD11c	TACGCCTATAACTTG	A0053	S-HCL-3	proprietary	CD11c	399907	BioLegend
CD54	CTGATAGACTTGAGT	A0217	HA58	proprietary	CD54	399907	BioLegend
CD268 (BAFF-R)	CGAAGTCGATCCGTA	A0215	11C1	proprietary	CD268--BAFF-R	399907	BioLegend
TIM-4	CGTCATATAGTATGG	A0428	9F4	proprietary	Tim-4	399907	BioLegend
CD1a	GATCGTGTTGTGTTA	A0402	HI149	proprietary	CD1a	399907	BioLegend
CD301 (CLEC10A)	ACCTAGAAATCAGCA	A0401	H037G3	proprietary	CD301--CLEC10A	399907	BioLegend
CD63	GAGATGTCTGCAACT	A0404	H5C6	proprietary	CD63.1	399907	BioLegend
CD44	AATCCTTCCGAATGT	A0125	BJ18	proprietary	CD44.1	399907	BioLegend
IgD	CAGTCTCCGTAGAGT	A0384	IA6-2	proprietary	IgD	399907	BioLegend
CD72	CAGTCGTGGTAGATA	A0419	3F3	proprietary	CD72.1	399907	BioLegend
CD93	GCGCTACTTCCTTGA	A0446	VIMD2	proprietary	CD93.1	399907	BioLegend
CD85g (ILT7)	TGTCAGTTCCTATGA	A0409	17G10.2	proprietary	CD85g--ILT7	399907	BioLegend
CD172a (SIRPα)	CGTGTTTAACTTGAG	A0408	15-414	proprietary	CD172a--SIRPa	399907	BioLegend
CD284 (TLR4)	GCTTAGCTGTATCCG	A0405	HTA125	proprietary	CD284--TLR4	399907	BioLegend
CD304 (Neuropilin-1)	GGACTAAGTTTCGTT	A0406	12C2	proprietary	CD304--Neuropilin-1	399907	BioLegend
CD36	TTCTTTGCCTTGCCA	A0407	5-271	proprietary	CD36.1	399907	BioLegend
CD102 (ICAM-2)	TGACCTTCCTCTCCT	A0244	CBR-IC2/2	proprietary	CD102	399907	BioLegend
TCR α/β	CGTAACGTAGAGCGA	A0224	IP26	proprietary	TCR-A--TCR-B	399907	BioLegend
CD122 (IL-2R?)	TCATTTCCTCCGATT	A0246	TU27	proprietary	N/A	399907	BioLegend
CD83	CCACTCATTTCCGGT	A0359	HB15e	proprietary	CD83.1	399907	BioLegend
CD267 (TACI)	AGTGATGGAGCGAAC	A0247	1A1	proprietary	CD267--TACI	399907	BioLegend
anti-human IgA	AAGATGTCCGAGCAA	A0186	#N/A	proprietary	N/A	399907	BioLegend
CD107a (LAMP-1)	CAGCCCACTGCAATA	A0155	H4A3	proprietary	CD107a--LAMP-1	399907	BioLegend
CD146	CCTTGGATAACATCA	A0134	P1H12	proprietary	CD146	399907	BioLegend
CD140b (PDGFR?)	CAATGGTTCACTGCC	A0129	18A2	proprietary	CD140b	399907	BioLegend
CD49a	ACTGATGGACTCAGA	A0575	TS2/7	proprietary	CD49a	399907	BioLegend
α	AACTTCTGTGGTAGC	A0584	6B11	proprietary	TCR-V-A-24-J-A-18--iNKT cell	399907	BioLegend
CD9	GAGTCACCAATCTGC	A0579	HI9a	proprietary	CD9.1	399907	BioLegend
TCR Vα7.2	TACGAGCAGTATTCA	A0581	3C10	proprietary	TCR-V-A-7.2	399907	BioLegend
TCR Vγ9	AAGTGATGGTATCTG	A0583	B3	proprietary	TCR-V-G-9	399907	BioLegend
CD49d	CCATTCAACTTCCGG	A0576	9F10	proprietary	CD49d	399907	BioLegend
C5L2	ACAATTTGTCTGCGA	A0572	1D9-M12	proprietary	C5L2	399907	BioLegend
CD338 (ABCG2)	TAAGACTTGGCCGTC	A0569	5D3	proprietary	CD338--ABCG2	399907	BioLegend
CD73 (Ecto-5'-nucleotidase)	CAGTTCCTCAGTTCG	A0577	AD2	proprietary	CD73--Ecto-5-nucleotidase	399907	BioLegend
anti-human CD79a (Ig?)	CTTATCACCCGCTTT	A0578	#N/A	proprietary	N/A	399907	BioLegend
CD158b (KIR2DL2/L3, NKAT2)	GACCCGTAGTTTGAT	A0592	DX27	proprietary	CD158b--KIR2DL2--KIR2DL3--NKAT2	399907	BioLegend
CD226 (DNAM-1)	AGACCAACTCATTCA	A0805	TX25	proprietary	CD226--DNAM-1.1	399907	BioLegend
CD186 (CXCR6)	GACAGTCGATGCAAC	A0804	K041E5	proprietary	CD186--CXCR6	399907	BioLegend
CD158f (KIR2DL5)	AAAGTGATGCCACTG	A0600	UP-R1	proprietary	CD158f--KIR2DL5	399907	BioLegend
CD158e1 (KIR3DL1, NKB1)	GGACGCTTTCCTTGA	A0599	DX9	proprietary	CD158e1--KIR3DL1--NKB1	399907	BioLegend
CD140a (PDGFR?)	ATGCGCCGAGAATTA	A0128	16A1	proprietary	CD140a	399907	BioLegend
CD354 (TREM-1)	TAGCCGTTTCCTTTG	A0586	TREM-26	proprietary	N/A	399907	BioLegend
TCR Vδ2	TCAGTCAGATGGTAT	A0582	B6	proprietary	TCR-V-D-2	399907	BioLegend
CD202b (Tie2/Tek)	CGATCCCTTACCTAT	A0588	33.1 (Ab33)	proprietary	CD202b--Tie2--Tek	399907	BioLegend
CD96 (TACTILE)	TGGCCTATAAATGGT	A0175	NK92.39	proprietary	CD96--TACTILE	399907	BioLegend
CD158 (KIR2DL1/S1/S3/S5)	TATCAACCAACGCTT	A0420	HP-MA4	proprietary	CD158--KIR2DL1--KIR2DS1--KIR2DS3--KIR2DS5	399907	BioLegend
CD337 (NKp30)	AAAGTCACTCTGCCG	A0801	P30-15	proprietary	CD337--NKp30	399907	BioLegend
CD253 (Trail)	GCCATTCCTGCCTAA	A0803	RIK-2	proprietary	CD253--TRAIL	399907	BioLegend
CD319 (CRACC)	AGTATGCCATGTCTT	A0830	162.1	proprietary	CD319--CRACC	399907	BioLegend
CD305 (LAIR1)	ATTTCCATTCCCTGT	A0590	NKTA255	proprietary	CD305--LAIR1	399907	BioLegend
CD325 (N-Cadherin)	CCTTCCCTTTCCTCT	A0433	8C11	proprietary	CD325--N-Cadherin	399907	BioLegend
mast cell tryptase	ACTGATAGACCCGCT	A0580	AA1	proprietary	N/A	399907	BioLegend
CLEC12A	CATTAGAGTCTGCCA	A0853	50C1	proprietary	CD371--CLEC12A	399907	BioLegend
CD90 (Thy1)	GCATTGTACGATTCA	A0060	5E10	proprietary	CD90--Thy1	399907	BioLegend
CD273 (B7-DC, PD-L2)	TCAACGCTTGGCTAG	A0008	24F.10C12	proprietary	CD273--B7-DC--PD-L2	399907	BioLegend
CD272 (BTLA)	GTTATTGGACTAAGG	A0170	MIH26	proprietary	CD272--BTLA	399907	BioLegend
CD5	CATTAACGGGATGCC	A0138	UCHT2	proprietary	CD5.1	399907	BioLegend
CD23	TCTGTATAACCGTCT	A0897	EBVCS-5	proprietary	CD23	399907	BioLegend
CD85j (ILT2)	CCTTGTGAGGCTATG	A0896	GHI/75	proprietary	CD85j--ILT2	399907	BioLegend
CD94	CTTTCCGGTCCTACA	A0867	DX22	proprietary	CD94	399907	BioLegend
CD27	GCACTCCTGCATGTA	A0154	O323	proprietary	CD27.1	399907	BioLegend
CD328 (Siglec-7)	CTTAGCATTTCACTG	A0902	6-434	proprietary	CD328--Siglec-7	399907	BioLegend
CD82	TCCCACTTCCGCTTT	A0920	ASL-24	proprietary	N/A	399907	BioLegend
CD16	AAGTTCACTCTTTGC	A0083	3G8	proprietary	CD16	399907	BioLegend
MERTK	TCCTGCATGTACCCA	A0423	590H11G1E3	proprietary	MERTK.1	399907	BioLegend

	IgG Fc	CTGGAGCGATTAGAA	A0375	M1310G05	proprietary	IgG-Fc	399907	BioLegend
	FcεRIα	CTCGTTTCCGTATCG	A0352	AER-37 (CRA-1)	proprietary	FceRIa	399907	BioLegend
	CD274 (B7-H1, PD-L1)	GTTGTCCGACAATAC	A0007	29E.2A3	proprietary	CD274–B7-H1–PD-L1	399907	BioLegend
	CD254 (TRANCE, RANKL)	TCCGTGTTAGTTTGT	A0356	MIH24	proprietary	CD254–TRANCE–RANKL	399907	BioLegend
	CX3CR1	AGTATCGTCTCTGGG	A0179	K0124E1	proprietary	CX3CR1.1	399907	BioLegend
	CD137L (4-1BB Ligand)	ATTCGCCTTACGCAA	A0022	5F4	proprietary	CD137L–4-1BB-Ligand	399907	BioLegend
	Siglec-8	CTTCTCCTCAGCAAT	A0199	7C9	proprietary	N/A	399907	BioLegend
	CD45	89	Y	HI30	1.5		304002	BioLegend
	EpCAM	115	In	9C4	1.5		324202	BioLegend
	CD7	139	La	M-T701	1.5		555359	BD Bioscience
	CD15	140	Ce	W6D3	1.5		323002	BioLegend
	CD3	141	Pr	UCHT1	0.4		300402	BioLegend
	CD19	142	Nd	HIB19	0.4		302202	BioLegend
	CD163	144	Nd	GHI61	3		333602	BioLegend
	CD4	145	Nd	RPA-T4	0.4		14-0049-82	Thermo
	CD8a	146	Nd	RPA-T8	0.4		301002	BioLegend
	CD11c	147	Sm	BU15	0.4		MA1-82142	Thermo
	CD14	148	Nd	M5E2	1.5		301802	BioLegend
	CD127	149	Sm	A019D5	1.5		351302	BioLegend
	CD123	151	Eu	6H6	0.8		14-1239-82	Thermo
	gdTCR	152	Sm	5A6.E9	0.4		HB-9772	ATCC
	CD45RA	153	Eu	HI100	1.5		304102	BioLegend
	TIM3	154	Sm	F38-2E2	3		345002	BioLegend
	TIGIT	155	Gd	A15153G	1.5		372720	BioLegend
	PD-L1	156	Gd	29E.2A3	1.5		329702	BioLegend
CyTOF	Integrin a4	157	Gd	9F10	1.5		304302	BioLegend
	CD27	158	Gd	O323	0.4		302802	BioLegend
	CD137_4-1BB	159	Tb	4B4-1	1.5		14-9056-82	Thermo
	Tbet	160	Gd	4B10	0.4		14-5825-82	Thermo
	CTLA-4	161	Dy	14D3	0.4		14-1529-82	Thermo
	FOXP3	162	Dy	PCH101	0.8		14-4776-82	Thermo
	CD31	163	Dy	WM59	0.8		303102	BioLegend
	Integrin b7	165	Ho	FIB504	1.5		321202	BioLegend
	CD141_BDCA3	166	Er	M80	0.8		344102	BioLegend
	CCR7	167	Er	GO43H7	1.5		353202	BioLegend
	Ki-67	168	Er	Ki-67	0.8		350502	BioLegend
	CD25	169	Tm	M-A251	0.4		356102	BioLegend
	CD1c_BDCA1	170	Er	L161	0.8		331502	BioLegend
	CD38	172	Yb	HIT2	0.4		303502	BioLegend
	ICOS	173	Yb	C398.4A	0.8		313502	BioLegend
	HLA-DR	174	Yb	L243	1.5		307665	BioLegend
	PD-1	175	Lu	EH12.2H7	1.5		329902	BioLegend
	CD56	176	Yb	NCAM16.2	0.8		559043	BD Bioscience
	CD16	209	Bi	3G8	0.4		302049	BioLegend
	COL1A2	488		432721-T1				ACD bio-techne
	MS4A1	550		426771-T2				ACD bio-techne
	FLT3	650		419491-T3				ACD bio-techne
	ITG$\alpha 4$	488		408591-T4				ACD bio-techne
	MZB1	550		579241-T5				ACD bio-techne
RNA-ISH	*C1QA*	650		485451-T6				ACD bio-techne
	ITG$\beta 7$	488		605941-T7				ACD bio-techne
	MAdCAM-1	550		404711-T8				ACD bio-techne
	KRT8	650		310261-T9				ACD bio-techne
	PECAM-1	488		487381-T10				ACD bio-techne
	CD3E	550		553971-T11				ACD bio-techne
	PTPRC	650		601991-T12				ACD bio-techne
	HLA-A	AF750	BX004	EP1395Y	1:200		ab216653	Abcam
	CD34	ATTO550	BX020	QBEND/10	1:150		MA1-10205	Thermofisher
	CD4	Cy5	BX003	EPR6855	1:200		ab133616	Abcam
	CD20	AF750	BX007	L26	1:200		14-0202-82	Thermofisher
	CD14	ATTO550	BX037	EPR3653	1:500		ab133335	Abcam
	CD68	Cy5	BX015	KP1	1:200		MA5-13324	Thermofisher
	Vimentin	AF750	BX022	091D3	1:200		677802	BioLegend
	CD8	ATTO550	BX026	C8/144B	1:200		372908	BioLegend
	CD11c	Cy5	BX024	118/A5	1:200		14-9761-82	Thermofisher
	CD31	AF750	BX001	EP3095	1:100		ab134168	Abcam
	E-Cadherin	ATTO550	BX014	4A2C7	1:300		33-4000	Thermofisher
	CD45	Cy5	BX021	D9M8I	1:300		#13917	CST
	SMA	AF750	BX013	1A4	1:200		ab7817	Abcam
	CD45RO	ATTO550	BX017	UCHL1	1:200		304202	BioLegend
CODEX	CD3e	Cy5	BX045	EP449E	1:200		ab52959	Abcam
	Pan-cytokeratin	AF750	BX019	AE1/AE3	1:200		914204	BioLegend
	CD44	ATTO550	BX005	156-3C11	1:400		#3570	CST
	HLA-DR	Cy5	BX033	EPR3692	1:200		ab92511	Abcam
	Granzyme B	ATTO550	BX041	D6E9W	1:50		#46890	CST
	Collagen IV	Cy5	BX042	EPR209660	1:200		ab214417	Abcam
	Podoplanin	ATTO550	BX023	NC-08	1:200		337002	BioLegend
	FOXP3	AF647	BX031	259D/C7	1:100		560044	BD Pharmigen
	KI67	ATTO550	BX047	B56	1:300		556003	BD Pharmigen
	CD163	AF647	BX016	D6U1J	1:50		#93498	CST
	CD19	ATTO550	BX028	RM332	1:50		31-1219-00	RevMab
	CD11b	Cy5	BX030	EP1345Y	1:50		ab52478	Abcam
	CD21	ATTO550	BX032	EP3093	1:400		ab75985	Abcam
	CD57	AF647	BX049	HNK-1	1:100		359610	BioLegend
	dsDNA	89	Y	3519 DNA	0.2		ab27156	Abcam
	E-Cadherin	113	In	36	0.5		ab287971	Abcam
	Histone H3	115	In	D1H2	1:100		711501-100	Ionpath
	FoxP3-AF488	141	Pr	236A/E7	2		561181	BD Pharmigen
	anti-488	141	Pr	polyclonal	3		#A-11094	Thermofisher
	CD163	142	Nd	EPR14643-36	0.6		ab215976	Abcam
	CD4	143	Nd	EPR6855	1:100		714301-100	Ionpath

	CD11c	144	Nd	EP1347Y	1:100	714401-100	Ionpath
	CD14	145	Nd	D7A2T	0.6	#43878	CST
	CD16	146	Nd	D1N9L	0.6	#72204	CST
	LAG3	147	Sm	17B4	5	NBP1-97657	Novus
	PD-1	148	Nd	D4W2J	1:100	714801-100	Ionpath
	PD-L1	149	Sm	E1L3N	1:100	714902-100	Ionpath
	Granzyme B	150	Nd	D6E9W	1:100	715002-100	Ionpath
	CD56	151	Eu	MRQ-42	1:100	715101-100	Ionpath
	CD31	152	Sm	EP3095	1:100	715201-100	Ionpath
	Ki67	153	Eu	D2H10	0.15	#44092	CST
	CD138	154	Sm	EPR6454	5	ab2261108	Abcam
	CD117	155	Gd	YR145	1:100	715501-100	Ionpath
MIBI	CD68	156	Gd	D4B9C	1:100	715601-100	Ionpath
	CD103	157	Gd	EPR4166(2)	15	ab271889	Abcam
	CD8	158	Gd	C8/144B	1:100	715801-100	Ionpath
	CD3	159	Tb	D7A6E	1:100	715903-100	Ionpath
	Tbet	160	Gd	D6N8B	5	#27112	CST
	CD45RO	161	Dy	UCHL1	1:100	716101-100	ionpath
	TIM3	162	Dy	EPR22241	1:100	716201-100	Ionpath
	Vimentin	163	Dy	D21H3	1:100	716301-100	Ionpath
	CD27	164	Dy	EPR8569	5	ab256583	Abcam
	Pan-Keratin	165	Ho	AE1/AE3	1:100	716501-100	Ionpath
	aSMA	166	Er	SP171	2.5	ab242395	Abcam
	CD20	167	Er	L26	1:100	716701-100	Abcam
	CD11b	168	Er	D6X1N	0.5	ab187537	Abcam
	BDCA3 (CD141)	169	Tm	E7Y9P	10	#34149	CST
	CD21	170	Er	EP3093	1:100	717001-100	Ionpath
	IDO1	171	Yb	EPR20374	1:100	717101-100	Ionpath
	HLA-DR	172	Yb	EPR3692	1:100	717201-100	Ionpath
	EpCAM	173	Yb	D9S3P	0.5	#55725	CST
	CD45	175	Yb	2B11/PD7/26	1:100	717501-100	Ionpath
	HLA Class 1, ABC	176	Yb	EMR8-5	1:100	717602-100	Ionpath
CosMx SMI 960-plex panel	*AATK*						Nanostring
	ABL1						Nanostring
	ABL2						Nanostring
	ACE						Nanostring
	ACE2						Nanostring
	ACKR1						Nanostring
	ACKR3						Nanostring
	ACKR4						Nanostring
	ACTA2						Nanostring
	ACTG2						Nanostring
	ACVR1						Nanostring
	ACVR1B						Nanostring
	ACVR2A						Nanostring
	ACVRL1						Nanostring
	ADGRA2						Nanostring
	ADGRA3						Nanostring
	ADGRB2						Nanostring
	ADGRB3						Nanostring
	ADGRD1						Nanostring
	ADGRE1						Nanostring
	ADGRE2						Nanostring
	ADGRE5						Nanostring
	ADGRF1						Nanostring
	ADGRF3						Nanostring
	ADGRF4						Nanostring
	ADGRF5						Nanostring
	ADGRG1						Nanostring
	ADGRG2						Nanostring
	ADGRG3						Nanostring
	ADGRG5						Nanostring
	ADGRG6						Nanostring
	ADGRL1						Nanostring
	ADGRL2						Nanostring
	ADGRL4						Nanostring
	ADGRV1						Nanostring
	ADIPOQ						Nanostring
	ADIRF						Nanostring
	ADM2						Nanostring
	ADORA2A						Nanostring
	AGR2						Nanostring
	AHI1						Nanostring
	AHR						Nanostring
	AKT1						Nanostring
	ALCAM						Nanostring
	ANGPT1						Nanostring
	ANGPT2						Nanostring
	ANGPT4						Nanostring
	ANGPTL1						Nanostring
	ANXA1						Nanostring
	ANXA2						Nanostring
	ANXA4						Nanostring
	APOA1						Nanostring
	APOB						Nanostring
	APOD						Nanostring
	APP						Nanostring
	AQP3						Nanostring
	AR						Nanostring
	AREG						Nanostring
	ARF1						Nanostring

ARG1	Nanostring
ARHGDIB	Nanostring
ARTN	Nanostring
ATF3	Nanostring
ATG10	Nanostring
ATG12	Nanostring
ATG5	Nanostring
ATM	Nanostring
ATR	Nanostring
AXL	Nanostring
AZGP1	Nanostring
AZU1	Nanostring
B2M	Nanostring
B3GNT7	Nanostring
BAG3	Nanostring
BATF3	Nanostring
BAX	Nanostring
BCL2	Nanostring
BCL2L1	Nanostring
BECN1	Nanostring
BEST1	Nanostring
BGN	Nanostring
BID	Nanostring
BIRC5	Nanostring
BMP1	Nanostring
BMP2	Nanostring
BMP3	Nanostring
BMP4	Nanostring
BMP5	Nanostring
BMP6	Nanostring
BMP7	Nanostring
BMPR1A	Nanostring
BMPR2	Nanostring
BMX	Nanostring
BRCA1	Nanostring
BST1	Nanostring
BST2	Nanostring
BTG1	Nanostring
BTK	Nanostring
C11orf96	Nanostring
C1QA	Nanostring
C1QB	Nanostring
C1QC	Nanostring
C5AR2	Nanostring
C9orf16	Nanostring
CALB1	Nanostring
CALD1	Nanostring
CALM1	Nanostring
CALM2	Nanostring
CALM3	Nanostring
CAMP	Nanostring
CASP3	Nanostring
CASP8	Nanostring
CASR	Nanostring
CAV1	Nanostring
CCL11	Nanostring
CCL13	Nanostring
CCL15	Nanostring
CCL18	Nanostring
CCL19	Nanostring
CCL2	Nanostring
CCL20	Nanostring
CCL21	Nanostring
CCL23	Nanostring
CCL26	Nanostring
CCL28	Nanostring
CCL3	Nanostring
CCL3L3	Nanostring
CCL4	Nanostring
CCL4L2	Nanostring
CCL5	Nanostring
CCL7	Nanostring
CCL8	Nanostring
CCND1	Nanostring
CCR1	Nanostring
CCR10	Nanostring
CCR2	Nanostring
CCR5	Nanostring
CCR7	Nanostring
CCRL2	Nanostring
CD14	Nanostring
CD163	Nanostring
CD164	Nanostring
CD19	Nanostring
CD2	Nanostring
CD209	Nanostring
CD24	Nanostring
CD27	Nanostring
CD274	Nanostring
CD276	Nanostring
CD28	Nanostring

CD300A	Nanostring
CD33	Nanostring
CD34	Nanostring
CD36	Nanostring
CD37	Nanostring
CD38	Nanostring
CD3D	Nanostring
CD3E	Nanostring
CD3G	Nanostring
CD4	Nanostring
CD40	Nanostring
CD40LG	Nanostring
CD44	Nanostring
CD47	Nanostring
CD48	Nanostring
CD52	Nanostring
CD53	Nanostring
CD55	Nanostring
CD58	Nanostring
CD59	Nanostring
CD5L	Nanostring
CD63	Nanostring
CD68	Nanostring
CD69	Nanostring
CD70	Nanostring
CD74	Nanostring
CD79A	Nanostring
CD80	Nanostring
CD81	Nanostring
CD83	Nanostring
CD84	Nanostring
CD86	Nanostring
CD8A	Nanostring
CD8B	Nanostring
CD9	Nanostring
CDH1	Nanostring
CDH11	Nanostring
CDH5	Nanostring
CDKN1A	Nanostring
CDKN3	Nanostring
CEACAM1	Nanostring
CEACAM6	Nanostring
CELSR1	Nanostring
CELSR2	Nanostring
CENPF	Nanostring
CFD	Nanostring
CFLAR	Nanostring
CHEK1	Nanostring
CHEK2	Nanostring
CHGA	Nanostring
CHI3L1	Nanostring
CIDEA	Nanostring
CIITA	Nanostring
CLCF1	Nanostring
CLDN4	Nanostring
CLEC10A	Nanostring
CLEC12A	Nanostring
CLEC14A	Nanostring
CLEC1A	Nanostring
CLEC2B	Nanostring
CLEC2D	Nanostring
CLEC4A	Nanostring
CLEC4D	Nanostring
CLEC4E	Nanostring
CLEC5A	Nanostring
CLEC7A	Nanostring
CLOCK	Nanostring
CLU	Nanostring
CMKLR1	Nanostring
CNTFR	Nanostring
COL11A1	Nanostring
COL12A1	Nanostring
COL14A1	Nanostring
COL15A1	Nanostring
COL16A1	Nanostring
COL17A1	Nanostring
COL18A1	Nanostring
COL1A1	Nanostring
COL1A2	Nanostring
COL21A1	Nanostring
COL27A1	Nanostring
COL3A1	Nanostring
COL4A1	Nanostring
COL4A2	Nanostring
COL4A5	Nanostring
COL5A1	Nanostring
COL5A2	Nanostring
COL5A3	Nanostring
COL6A1	Nanostring
COL6A2	Nanostring
COL6A3	Nanostring

COL8A1	Nanostring
COL9A1	Nanostring
COL9A2	Nanostring
COL9A3	Nanostring
COTL1	Nanostring
CPA3	Nanostring
CPB1	Nanostring
CRIP1	Nanostring
CRP	Nanostring
CRYAB	Nanostring
CSF1	Nanostring
CSF1R	Nanostring
CSF2	Nanostring
CSF2RA	Nanostring
CSF2RB	Nanostring
CSF3	Nanostring
CSF3R	Nanostring
CSHL1	Nanostring
CSK	Nanostring
CST7	Nanostring
CTLA4	Nanostring
CTNNB1	Nanostring
CTSG	Nanostring
CTSW	Nanostring
CUZD1	Nanostring
CX3CL1	Nanostring
CX3CR1	Nanostring
CXCL1	Nanostring
CXCL10	Nanostring
CXCL12	Nanostring
CXCL14	Nanostring
CXCL16	Nanostring
CXCL17	Nanostring
CXCL2	Nanostring
CXCL3	Nanostring
CXCL5	Nanostring
CXCL6	Nanostring
CXCL8	Nanostring
CXCL9	Nanostring
CXCR1	Nanostring
CXCR2	Nanostring
CXCR3	Nanostring
CXCR4	Nanostring
CXCR5	Nanostring
CXCR6	Nanostring
CYP19A1	Nanostring
CYP1B1	Nanostring
CYSTM1	Nanostring
CYTOR	Nanostring
DCN	Nanostring
DDC	Nanostring
DDIT3	Nanostring
DDR1	Nanostring
DDR2	Nanostring
DDX58	Nanostring
DHRS2	Nanostring
DLL1	Nanostring
DMBT1	Nanostring
DNMT1	Nanostring
DNMT3A	Nanostring
DNTT	Nanostring
DPP4	Nanostring
DST	Nanostring
DUSP1	Nanostring
DUSP2	Nanostring
DUSP4	Nanostring
DUSP5	Nanostring
DUSP6	Nanostring
EFNA1	Nanostring
EFNA4	Nanostring
EFNA5	Nanostring
EFNB1	Nanostring
EFNB2	Nanostring
EFNB3	Nanostring
EGF	Nanostring
EGFR	Nanostring
EIF5A	Nanostring
ELANE	Nanostring
EMP3	Nanostring
ENG	Nanostring
ENTPD1	Nanostring
EOMES	Nanostring
EPCAM	Nanostring
EPHA2	Nanostring
EPHA3	Nanostring
EPHA4	Nanostring
EPHA7	Nanostring
EPHB2	Nanostring
EPHB3	Nanostring
EPHB4	Nanostring
EPHB6	Nanostring

EPOR	Nanostring
ERBB2	Nanostring
ERBB3	Nanostring
ESAM	Nanostring
ESR1	Nanostring
ETS1	Nanostring
ETV4	Nanostring
ETV5	Nanostring
EZH2	Nanostring
EZR	Nanostring
FABP4	Nanostring
FABP5	Nanostring
FAS	Nanostring
FASLG	Nanostring
FASN	Nanostring
FCER1G	Nanostring
FCGBP	Nanostring
FCGR3A	Nanostring
FCRLA	Nanostring
FES	Nanostring
FFAR2	Nanostring
FFAR3	Nanostring
FFAR4	Nanostring
FGF1	Nanostring
FGF12	Nanostring
FGF13	Nanostring
FGF18	Nanostring
FGF2	Nanostring
FGF7	Nanostring
FGF9	Nanostring
FGFR1	Nanostring
FGFR2	Nanostring
FGFR3	Nanostring
FGG	Nanostring
FGR	Nanostring
FKBP11	Nanostring
FLT1	Nanostring
FLT3LG	Nanostring
FN1	Nanostring
FOS	Nanostring
FOXF1	Nanostring
FOXP3	Nanostring
FPR1	Nanostring
FYB1	Nanostring
FYN	Nanostring
FZD1	Nanostring
FZD3	Nanostring
FZD4	Nanostring
FZD5	Nanostring
FZD6	Nanostring
FZD7	Nanostring
FZD8	Nanostring
G6PC2	Nanostring
G6PD	Nanostring
GADD45B	Nanostring
GAS6	Nanostring
GATA3	Nanostring
GC	Nanostring
GCG	Nanostring
GDF10	Nanostring
GDF15	Nanostring
GDF3	Nanostring
GDF6	Nanostring
GDF9	Nanostring
GDNF	Nanostring
GLUD1	Nanostring
GLUL	Nanostring
GNLY	Nanostring
GPBAR1	Nanostring
GPER1	Nanostring
GPNMB	Nanostring
GPR183	Nanostring
GPX1	Nanostring
GPX3	Nanostring
GSN	Nanostring
GSTP1	Nanostring
GZMA	Nanostring
GZMB	Nanostring
GZMH	Nanostring
GZMK	Nanostring
H2AZ1	Nanostring
H4C3	Nanostring
HAVCR2	Nanostring
HBA1	Nanostring
HBB	Nanostring
HCAR2	Nanostring
HCAR3	Nanostring
HCK	Nanostring
HCST	Nanostring
HDAC1	Nanostring
HDAC11	Nanostring

HDAC3	Nanostring
HDAC4	Nanostring
HDAC5	Nanostring
HGF	Nanostring
HIF1A	Nanostring
HILPDA	Nanostring
HLA-A	Nanostring
HLA-B	Nanostring
HLA-C	Nanostring
HLA-DPA1	Nanostring
HLA-DPB1	Nanostring
HLA-DQA1	Nanostring
HLA-DQB1	Nanostring
HLA-DRA	Nanostring
HLA-DRB1	Nanostring
HLA-DRB5	Nanostring
HLA-E	Nanostring
HMGB2	Nanostring
HMGN2	Nanostring
HPGDS	Nanostring
HSD17B2	Nanostring
HSD3B2	Nanostring
HSP90AA1	Nanostring
HSP90AB1	Nanostring
HSP90B1	Nanostring
HSPA1A	Nanostring
HSPA1B	Nanostring
HSPB1	Nanostring
HTT	Nanostring
IAPP	Nanostring
ICAM1	Nanostring
ICAM2	Nanostring
ICAM3	Nanostring
ICOS	Nanostring
ICOSLG	Nanostring
IDO1	Nanostring
IER3	Nanostring
IFI27	Nanostring
IFIH1	Nanostring
IFIT1	Nanostring
IFITM1	Nanostring
IFITM3	Nanostring
IFNA1	Nanostring
IFNAR1	Nanostring
IFNAR2	Nanostring
IFNB1	Nanostring
IFNG	Nanostring
IFNGR1	Nanostring
IFNGR2	Nanostring
IFNL2	Nanostring
IFNL3	Nanostring
IGF1	Nanostring
IGF1R	Nanostring
IGF2	Nanostring
IGF2R	Nanostring
IGFBP3	Nanostring
IGFBP5	Nanostring
IGFBP6	Nanostring
IGFBP7	Nanostring
IGHA1	Nanostring
IGHD	Nanostring
IGHG1	Nanostring
IGHG2	Nanostring
IGHM	Nanostring
IGKC	Nanostring
IL10	Nanostring
IL10RA	Nanostring
IL10RB	Nanostring
IL11	Nanostring
IL11RA	Nanostring
IL12A	Nanostring
IL12B	Nanostring
IL12RB1	Nanostring
IL12RB2	Nanostring
IL13RA1	Nanostring
IL15	Nanostring
IL15RA	Nanostring
IL16	Nanostring
IL17A	Nanostring
IL17B	Nanostring
IL17D	Nanostring
IL17RA	Nanostring
IL17RB	Nanostring
IL17RE	Nanostring
IL18	Nanostring
IL18R1	Nanostring
IL1A	Nanostring
IL1B	Nanostring
IL1R1	Nanostring
IL1R2	Nanostring
IL1RAP	Nanostring

IL1RL1	Nanostring
IL1RN	Nanostring
IL2	Nanostring
IL20	Nanostring
IL20RA	Nanostring
IL22RA1	Nanostring
IL23A	Nanostring
IL24	Nanostring
IL27RA	Nanostring
IL2RA	Nanostring
IL2RB	Nanostring
IL2RG	Nanostring
IL32	Nanostring
IL33	Nanostring
IL34	Nanostring
IL36G	Nanostring
IL3RA	Nanostring
IL4R	Nanostring
IL6	Nanostring
IL6R	Nanostring
IL6ST	Nanostring
IL7	Nanostring
IL7R	Nanostring
INHA	Nanostring
INHBA	Nanostring
INHBB	Nanostring
INS	Nanostring
INSR	Nanostring
IRF3	Nanostring
IRF4	Nanostring
ITGA1	Nanostring
ITGA2	Nanostring
ITGA3	Nanostring
ITGA5	Nanostring
ITGA6	Nanostring
ITGA9	Nanostring
ITGAE	Nanostring
ITGAL	Nanostring
ITGAM	Nanostring
ITGAV	Nanostring
ITGAX	Nanostring
ITGB1	Nanostring
ITGB2	Nanostring
ITGB4	Nanostring
ITGB5	Nanostring
ITGB6	Nanostring
ITGB8	Nanostring
ITK	Nanostring
ITM2A	Nanostring
JAG1	Nanostring
JAK1	Nanostring
JAK2	Nanostring
JCHAIN	Nanostring
JUN	Nanostring
JUNB	Nanostring
KDR	Nanostring
KIT	Nanostring
KITLG	Nanostring
KLF2	Nanostring
KLK3	Nanostring
KLRB1	Nanostring
KLRK1	Nanostring
KRAS	Nanostring
KRT1	Nanostring
KRT10	Nanostring
KRT13	Nanostring
KRT14	Nanostring
KRT15	Nanostring
KRT16	Nanostring
KRT17	Nanostring
KRT18	Nanostring
KRT19	Nanostring
KRT20	Nanostring
KRT23	Nanostring
KRT24	Nanostring
KRT4	Nanostring
KRT5	Nanostring
KRT6A	Nanostring
KRT6B	Nanostring
KRT6C	Nanostring
KRT7	Nanostring
KRT8	Nanostring
KRT80	Nanostring
KRT86	Nanostring
LAG3	Nanostring
LAIR1	Nanostring
LAMP2	Nanostring
LAMP3	Nanostring
LCN2	Nanostring
LDLR	Nanostring
LEFTY1	Nanostring

LEFTY2	Nanostring
LEP	Nanostring
LGALS1	Nanostring
LGALS3	Nanostring
LGALS3BP	Nanostring
LGALS9	Nanostring
LIF	Nanostring
LIFR	Nanostring
LINC02446	Nanostring
LMNA	Nanostring
LPAR5	Nanostring
LTB	Nanostring
LTBR	Nanostring
LTF	Nanostring
LUM	Nanostring
LY6D	Nanostring
LY75	Nanostring
LYN	Nanostring
LYZ	Nanostring
MAF	Nanostring
MALAT1	Nanostring
MAML2	Nanostring
MAP1LC3B	Nanostring
MAPK13	Nanostring
MAPK14	Nanostring
MARCO	Nanostring
MECOM	Nanostring
MEG3	Nanostring
MERTK	Nanostring
MET	Nanostring
MGP	Nanostring
MIF	Nanostring
MKI67	Nanostring
MMP1	Nanostring
MMP10	Nanostring
MMP12	Nanostring
MMP14	Nanostring
MMP16	Nanostring
MMP19	Nanostring
MMP2	Nanostring
MMP3	Nanostring
MMP7	Nanostring
MMP8	Nanostring
MMP9	Nanostring
MPO	Nanostring
MRC1	Nanostring
MRC2	Nanostring
MS4A1	Nanostring
MS4A4A	Nanostring
MSMB	Nanostring
MST1R	Nanostring
MT1X	Nanostring
MT2A	Nanostring
MTOR	Nanostring
MTRNR2L1	Nanostring
MX1	Nanostring
MXRA8	Nanostring
MYC	Nanostring
MYH11	Nanostring
MYL9	Nanostring
MZB1	Nanostring
MZT2A	Nanostring
NANOG	Nanostring
NCR1	Nanostring
NDRG1	Nanostring
NEAT1	Nanostring
NFKB1	Nanostring
NFKBIA	Nanostring
NGFR	Nanostring
NKG7	Nanostring
NLRC4	Nanostring
NLRC5	Nanostring
NLRP1	Nanostring
NLRP2	Nanostring
NLRP3	Nanostring
NOD2	Nanostring
NOSIP	Nanostring
NOTCH1	Nanostring
NOTCH2	Nanostring
NOTCH3	Nanostring
NPPB	Nanostring
NPPC	Nanostring
NPR1	Nanostring
NPR2	Nanostring
NPR3	Nanostring
NR1H2	Nanostring
NR1H3	Nanostring
NR1H4	Nanostring
NR3C1	Nanostring
NRG1	Nanostring
NRG4	Nanostring

NRIP3	Nanostring
NRXN1	Nanostring
NRXN3	Nanostring
NTRK2	Nanostring
OAS1	Nanostring
OAS2	Nanostring
OAS3	Nanostring
OASL	Nanostring
OLFM4	Nanostring
OLR1	Nanostring
OSM	Nanostring
OSMR	Nanostring
OXER1	Nanostring
OXGR1	Nanostring
P2RX5	Nanostring
P2RY12	Nanostring
PARP1	Nanostring
PCNA	Nanostring
PDCD1	Nanostring
PDCD1LG2	Nanostring
PDGFA	Nanostring
PDGFB	Nanostring
PDGFC	Nanostring
PDGFD	Nanostring
PDGFRA	Nanostring
PDGFRB	Nanostring
PECAM1	Nanostring
PF4	Nanostring
PGF	Nanostring
PGR	Nanostring
PHLDA2	Nanostring
PIGR	Nanostring
PLA2R1	Nanostring
PLAC8	Nanostring
PNOC	Nanostring
POU5F1	Nanostring
PPARA	Nanostring
PPARD	Nanostring
PPARG	Nanostring
PPBP	Nanostring
PRF1	Nanostring
PROK2	Nanostring
PROKR1	Nanostring
PRSS2	Nanostring
PSAP	Nanostring
PSCA	Nanostring
PTGDR2	Nanostring
PTGDS	Nanostring
PTGES	Nanostring
PTGES2	Nanostring
PTGES3	Nanostring
PTGIS	Nanostring
PTGS1	Nanostring
PTGS2	Nanostring
PTHLH	Nanostring
PTK2	Nanostring
PTK6	Nanostring
PTPRC	Nanostring
PTPRCAP	Nanostring
PTTG1	Nanostring
QRFPR	Nanostring
RAC1	Nanostring
RAC2	Nanostring
RAD51	Nanostring
RAMP1	Nanostring
RAMP2	Nanostring
RAMP3	Nanostring
RARA	Nanostring
RARB	Nanostring
RARG	Nanostring
RARRES1	Nanostring
RARRES2	Nanostring
RB1	Nanostring
RBPJ	Nanostring
REG1A	Nanostring
RELA	Nanostring
RELT	Nanostring
RGCC	Nanostring
RGS1	Nanostring
RGS2	Nanostring
RGS5	Nanostring
RNF43	Nanostring
ROR1	Nanostring
RORA	Nanostring
RPL21	Nanostring
RPL22	Nanostring
RPL32	Nanostring
RPL34	Nanostring
RPL37	Nanostring
RPS4Y1	Nanostring
RSPO1	Nanostring

RSPO2	Nanostring
RSPO3	Nanostring
RUNX3	Nanostring
RXRA	Nanostring
RXRB	Nanostring
RYK	Nanostring
S100A10	Nanostring
S100A2	Nanostring
S100A4	Nanostring
S100A6	Nanostring
S100A8	Nanostring
S100A9	Nanostring
S100B	Nanostring
S100P	Nanostring
SAA1	Nanostring
SAA2	Nanostring
SAT1	Nanostring
SCG5	Nanostring
SCGB3A1	Nanostring
SEC23A	Nanostring
SEC61G	Nanostring
SELENOP	Nanostring
SELL	Nanostring
SELPLG	Nanostring
SERPINA1	Nanostring
SERPINA3	Nanostring
SERPINB5	Nanostring
SERPINH1	Nanostring
SFN	Nanostring
SIGIRR	Nanostring
SLC2A1	Nanostring
SLC2A4	Nanostring
SLC40A1	Nanostring
SLPI	Nanostring
SMAD2	Nanostring
SMAD3	Nanostring
SMAD4	Nanostring
SMARCB1	Nanostring
SMO	Nanostring
SNAI1	Nanostring
SNAI2	Nanostring
SOD1	Nanostring
SOD2	Nanostring
SOSTDC1	Nanostring
SOX2	Nanostring
SOX4	Nanostring
SOX9	Nanostring
SPARCL1	Nanostring
SPINK1	Nanostring
SPOCK2	Nanostring
SPP1	Nanostring
SPRY2	Nanostring
SPRY4	Nanostring
SQSTM1	Nanostring
SRC	Nanostring
SREBF1	Nanostring
SRGN	Nanostring
SST	Nanostring
ST6GAL1	Nanostring
ST6GALNAC3	Nanostring
STAT1	Nanostring
STAT3	Nanostring
STAT4	Nanostring
STAT5A	Nanostring
STAT5B	Nanostring
STAT6	Nanostring
STMN1	Nanostring
SUCNR1	Nanostring
SYK	Nanostring
TACSTD2	Nanostring
TAGLN	Nanostring
TAP1	Nanostring
TAP2	Nanostring
TBX21	Nanostring
TCL1A	Nanostring
TEK	Nanostring
TFEB	Nanostring
TGFB1	Nanostring
TGFB2	Nanostring
TGFB3	Nanostring
TGFBR1	Nanostring
TGFBR2	Nanostring
THBS1	Nanostring
THBS2	Nanostring
TIE1	Nanostring
TIGIT	Nanostring
TIMP1	Nanostring
TLR1	Nanostring
TLR2	Nanostring
TLR3	Nanostring
TLR4	Nanostring

	TLR5	Nanostring
	TLR7	Nanostring
	TLR8	Nanostring
	TM4SF1	Nanostring
	TNF	Nanostring
	TNFAIP6	Nanostring
	TNFRSF10A	Nanostring
	TNFRSF10B	Nanostring
	TNFRSF10D	Nanostring
	TNFRSF11A	Nanostring
	TNFRSF11B	Nanostring
	TNFRSF12A	Nanostring
	TNFRSF13B	Nanostring
	TNFRSF14	Nanostring
	TNFRSF17	Nanostring
	TNFRSF18	Nanostring
	TNFRSF19	Nanostring
	TNFRSF1A	Nanostring
	TNFRSF1B	Nanostring
	TNFRSF21	Nanostring
	TNFRSF4	Nanostring
	TNFRSF9	Nanostring
	TNFSF10	Nanostring
	TNFSF12	Nanostring
	TNFSF13B	Nanostring
	TNFSF14	Nanostring
	TNFSF15	Nanostring
	TNFSF18	Nanostring
	TNFSF4	Nanostring
	TNFSF8	Nanostring
	TNFSF9	Nanostring
	TOP2A	Nanostring
	TOX	Nanostring
	TP53	Nanostring
	TPM1	Nanostring
	TPM2	Nanostring
	TPSAB1	Nanostring
	TPSB2	Nanostring
	TSC22D1	Nanostring
	TSHZ2	Nanostring
	TSLP	Nanostring
	TTR	Nanostring
	TUBB	Nanostring
	TUBB4B	Nanostring
	TWIST1	Nanostring
	TWIST2	Nanostring
	TXK	Nanostring
	TYK2	Nanostring
	TYMS	Nanostring
	TYROBP	Nanostring
	UBE2C	Nanostring
	UCP1	Nanostring
	UPK3A	Nanostring
	VCAM1	Nanostring
	VCAN	Nanostring
	VEGFA	Nanostring
	VEGFB	Nanostring
	VEGFC	Nanostring
	VEGFD	Nanostring
	VHL	Nanostring
	VIM	Nanostring
	VPREB3	Nanostring
	VSIR	Nanostring
	VTN	Nanostring
	VWF	Nanostring
	WIF1	Nanostring
	WNT10B	Nanostring
	WNT11	Nanostring
	WNT2	Nanostring
	WNT2B	Nanostring
	WNT3	Nanostring
	WNT5A	Nanostring
	WNT5B	Nanostring
	WNT7A	Nanostring
	WNT7B	Nanostring
	WNT9A	Nanostring
	XBP1	Nanostring
	XCL1	Nanostring
	XCL2	Nanostring
	YBX3	Nanostring
	YES1	Nanostring
	ZFP36	Nanostring
CosMx SMI 1000-plex panel	*AATK*	Nanostring
	ABL1	Nanostring
	ABL2	Nanostring
	ACACB	Nanostring
	ACE	Nanostring
	ACKR1	Nanostring
	ACKR3	Nanostring
	ACKR4	Nanostring
	ACP5	Nanostring

ACTA2	Nanostring
ACTG2	Nanostring
ACVR1	Nanostring
ACVR1B	Nanostring
ACVR2A	Nanostring
ACVRL1	Nanostring
ADGRA2	Nanostring
ADGRA3	Nanostring
ADGRE2	Nanostring
ADGRE5	Nanostring
ADGRF1	Nanostring
ADGRF3	Nanostring
ADGRF5	Nanostring
ADGRG1	Nanostring
ADGRG3	Nanostring
ADGRG5	Nanostring
ADGRG6	Nanostring
ADGRL1	Nanostring
ADGRL2	Nanostring
ADGRL4	Nanostring
ADGRV1	Nanostring
ADIPOQ	Nanostring
ADIRF	Nanostring
ADM2	Nanostring
AGR2	Nanostring
AHI1	Nanostring
AHR	Nanostring
AIF1	Nanostring
AKT1	Nanostring
ALCAM	Nanostring
ALOX5AP	Nanostring
ANGPT1	Nanostring
ANGPT2	Nanostring
ANGPTL1	Nanostring
ANKRD1	Nanostring
ANXA1	Nanostring
ANXA2	Nanostring
ANXA4	Nanostring
APOA1	Nanostring
APOC1	Nanostring
APOD	Nanostring
APOE	Nanostring
APP	Nanostring
AQP3	Nanostring
AR	Nanostring
AREG	Nanostring
ARF1	Nanostring
ARG1	Nanostring
ARHGDIB	Nanostring
ARID5B	Nanostring
ATF3	Nanostring
ATG10	Nanostring
ATG12	Nanostring
ATG5	Nanostring
ATM	Nanostring
ATP5F1B	Nanostring
ATP5F1E	Nanostring
ATR	Nanostring
AXL	Nanostring
AZGP1	Nanostring
AZU1	Nanostring
B2M	Nanostring
B3GNT7	Nanostring
BAG3	Nanostring
BASP1	Nanostring
BAX	Nanostring
BBLN	Nanostring
BCL2	Nanostring
BCL2L1	Nanostring
BECN1	Nanostring
BEST1	Nanostring
BGN	Nanostring
BID	Nanostring
BIRC3	Nanostring
BIRC5	Nanostring
BMP1	Nanostring
BMP2	Nanostring
BMP3	Nanostring
BMP4	Nanostring
BMP5	Nanostring
BMP7	Nanostring
BMPR1A	Nanostring
BMPR2	Nanostring
BRAF	Nanostring
BRCA1	Nanostring
BST1	Nanostring
BST2	Nanostring
BTF3	Nanostring
BTG1	Nanostring
BTK	Nanostring
C11orf96	Nanostring

C1QA	Nanostring
C1QB	Nanostring
C1QC	Nanostring
C5AR2	Nanostring
CACNA1C	Nanostring
CALB1	Nanostring
CALD1	Nanostring
CALM1	Nanostring
CALM2	Nanostring
CALM3	Nanostring
CAMP	Nanostring
CARMN	Nanostring
CASP3	Nanostring
CASP8	Nanostring
CASR	Nanostring
CAV1	Nanostring
CCDC80	Nanostring
CCL11	Nanostring
CCL13	Nanostring
CCL15	Nanostring
CCL17	Nanostring
CCL18	Nanostring
CCL19	Nanostring
CCL2	Nanostring
CCL20	Nanostring
CCL21	Nanostring
CCL22	Nanostring
CCL26	Nanostring
CCL28	Nanostring
CCL3/L1/L3	Nanostring
CCL4/L1/L2	Nanostring
CCL5	Nanostring
CCL8	Nanostring
CCND1	Nanostring
CCR1	Nanostring
CCR10	Nanostring
CCR2	Nanostring
CCR5	Nanostring
CCR7	Nanostring
CCRL2	Nanostring
CD14	Nanostring
CD163	Nanostring
CD164	Nanostring
CD19	Nanostring
CD1C	Nanostring
CD2	Nanostring
CD209	Nanostring
CD22	Nanostring
CD24	Nanostring
CD27	Nanostring
CD274	Nanostring
CD276	Nanostring
CD28	Nanostring
CD300A	Nanostring
CD33	Nanostring
CD34	Nanostring
CD36	Nanostring
CD37	Nanostring
CD38	Nanostring
CD3D	Nanostring
CD3E	Nanostring
CD3G	Nanostring
CD4	Nanostring
CD40	Nanostring
CD40LG	Nanostring
CD44	Nanostring
CD47	Nanostring
CD48	Nanostring
CD52	Nanostring
CD53	Nanostring
CD55	Nanostring
CD58	Nanostring
CD59	Nanostring
CD5L	Nanostring
CD63	Nanostring
CD68	Nanostring
CD69	Nanostring
CD70	Nanostring
CD74	Nanostring
CD79A	Nanostring
CD80	Nanostring
CD81	Nanostring
CD83	Nanostring
CD84	Nanostring
CD86	Nanostring
CD8A	Nanostring
CD8B	Nanostring
CD9	Nanostring
CD93	Nanostring
CDH1	Nanostring
CDH11	Nanostring

CDH19	Nanostring
CDH5	Nanostring
CDKN1A	Nanostring
CDKN3	Nanostring
CEACAM1	Nanostring
CEACAM6	Nanostring
CELSR1	Nanostring
CELSR2	Nanostring
CENPF	Nanostring
CFD	Nanostring
CFLAR	Nanostring
CHEK1	Nanostring
CHEK2	Nanostring
CHI3L1	Nanostring
CIDEA	Nanostring
CIITA	Nanostring
CLCF1	Nanostring
CLDN4	Nanostring
CLEC10A	Nanostring
CLEC12A	Nanostring
CLEC14A	Nanostring
CLEC1A	Nanostring
CLEC2B	Nanostring
CLEC2D	Nanostring
CLEC4A	Nanostring
CLEC4D	Nanostring
CLEC4E	Nanostring
CLEC5A	Nanostring
CLEC7A	Nanostring
CLOCK	Nanostring
CLU	Nanostring
CMKLR1	Nanostring
CNTFR	Nanostring
COL11A1	Nanostring
COL12A1	Nanostring
COL14A1	Nanostring
COL15A1	Nanostring
COL16A1	Nanostring
COL17A1	Nanostring
COL18A1	Nanostring
COL1A1	Nanostring
COL1A2	Nanostring
COL21A1	Nanostring
COL27A1	Nanostring
COL3A1	Nanostring
COL4A1	Nanostring
COL4A2	Nanostring
COL4A5	Nanostring
COL5A1	Nanostring
COL5A2	Nanostring
COL5A3	Nanostring
COL6A1	Nanostring
COL6A2	Nanostring
COL6A3	Nanostring
COL8A1	Nanostring
COL9A2	Nanostring
COL9A3	Nanostring
COTL1	Nanostring
COX4I2	Nanostring
CPA3	Nanostring
CPB1	Nanostring
CRIP1	Nanostring
CRP	Nanostring
CRYAB	Nanostring
CSF1	Nanostring
CSF1R	Nanostring
CSF2	Nanostring
CSF2RA	Nanostring
CSF2RB	Nanostring
CSF3	Nanostring
CSF3R	Nanostring
CSHL1	Nanostring
CSK	Nanostring
CSPG4	Nanostring
CST7	Nanostring
CSTB	Nanostring
CTLA4	Nanostring
CTNNB1	Nanostring
CTSD	Nanostring
CTSG	Nanostring
CTSW	Nanostring
CUZD1	Nanostring
CX3CL1	Nanostring
CX3CR1	Nanostring
CXCL1/2/3	Nanostring
CXCL10	Nanostring
CXCL12	Nanostring
CXCL13	Nanostring
CXCL14	Nanostring
CXCL16	Nanostring
CXCL17	Nanostring

CXCL5	Nanostring
CXCL8	Nanostring
CXCL9	Nanostring
CXCR1	Nanostring
CXCR2	Nanostring
CXCR3	Nanostring
CXCR4	Nanostring
CXCR5	Nanostring
CXCR6	Nanostring
CYP1B1	Nanostring
CYP2U1	Nanostring
CYSTM1	Nanostring
CYTOR	Nanostring
DCN	Nanostring
DDC	Nanostring
DDIT3	Nanostring
DDR1	Nanostring
DDR2	Nanostring
DDX58	Nanostring
DHRS2	Nanostring
DLL1	Nanostring
DLL4	Nanostring
DMBT1	Nanostring
DNMT1	Nanostring
DNMT3A	Nanostring
DPP4	Nanostring
DPT	Nanostring
DST	Nanostring
DUSP1	Nanostring
DUSP2	Nanostring
DUSP4	Nanostring
DUSP5	Nanostring
DUSP6	Nanostring
EFNA1	Nanostring
EFNA4	Nanostring
EFNA5	Nanostring
EFNB1	Nanostring
EFNB2	Nanostring
EGF	Nanostring
EGFR	Nanostring
EIF5A/L1	Nanostring
ELANE	Nanostring
EMP3	Nanostring
ENG	Nanostring
ENO1	Nanostring
ENTPD1	Nanostring
EOMES	Nanostring
EPCAM	Nanostring
EPHA2	Nanostring
EPHA3	Nanostring
EPHA4	Nanostring
EPHA7	Nanostring
EPHB2	Nanostring
EPHB3	Nanostring
EPHB4	Nanostring
EPHB6	Nanostring
EPOR	Nanostring
ERBB2	Nanostring
ERBB3	Nanostring
ESAM	Nanostring
ESR1	Nanostring
ETS1	Nanostring
ETV4	Nanostring
ETV5	Nanostring
EZH2	Nanostring
EZR	Nanostring
FABP4	Nanostring
FABP5	Nanostring
FAM30A	Nanostring
FAP	Nanostring
FAS	Nanostring
FASLG	Nanostring
FASN	Nanostring
FAU	Nanostring
FCER1G	Nanostring
FCGBP	Nanostring
FCGR3A/B	Nanostring
FCRLA	Nanostring
FES	Nanostring
FFAR2	Nanostring
FFAR3	Nanostring
FFAR4	Nanostring
FGF1	Nanostring
FGF12	Nanostring
FGF13	Nanostring
FGF18	Nanostring
FGF2	Nanostring
FGF7	Nanostring
FGF9	Nanostring
FGFR1	Nanostring
FGFR2	Nanostring

FGFR3	Nanostring
FGG	Nanostring
FGR	Nanostring
FHIT	Nanostring
FKBP11	Nanostring
FKBP5	Nanostring
FLT1	Nanostring
FLT3LG	Nanostring
FN1	Nanostring
FOS	Nanostring
FOXF1	Nanostring
FOXP3	Nanostring
FPR1	Nanostring
FYB1	Nanostring
FYN	Nanostring
FZD1	Nanostring
FZD3	Nanostring
FZD4	Nanostring
FZD5	Nanostring
FZD6	Nanostring
FZD7	Nanostring
FZD8	Nanostring
G0S2	Nanostring
G6PD	Nanostring
GADD45B	Nanostring
GAS6	Nanostring
GATA3	Nanostring
GC	Nanostring
GCG	Nanostring
GDF15	Nanostring
GLUD1	Nanostring
GLUL	Nanostring
GNLY	Nanostring
GPBAR1	Nanostring
GPER1	Nanostring
GPNMB	Nanostring
GPR183	Nanostring
GPX1	Nanostring
GPX3	Nanostring
GSK3B	Nanostring
GSN	Nanostring
GSTP1	Nanostring
GZMA	Nanostring
GZMB	Nanostring
GZMH	Nanostring
GZMK	Nanostring
H2AZ1	Nanostring
H4C3	Nanostring
HAVCR2	Nanostring
HBA1/2	Nanostring
HBB	Nanostring
HCAR2/3	Nanostring
HCK	Nanostring
HCST	Nanostring
HDAC1	Nanostring
HDAC11	Nanostring
HDAC3	Nanostring
HDAC4	Nanostring
HDAC5	Nanostring
HEXB	Nanostring
HEY1	Nanostring
HGF	Nanostring
HIF1A	Nanostring
HILPDA	Nanostring
HLA-DPA1	Nanostring
HLA-DPB1	Nanostring
HLA-DQA1	Nanostring
HLA-DQB1/2	Nanostring
HLA-DRA	Nanostring
HLA-DRB	Nanostring
HMGB2	Nanostring
HMGCS1	Nanostring
HMGN2	Nanostring
HPGDS	Nanostring
HSD17B2	Nanostring
HSP90AA1	Nanostring
HSP90AB1	Nanostring
HSP90B1	Nanostring
HSPA1A/B	Nanostring
HSPB1	Nanostring
HSPG2	Nanostring
HTT	Nanostring
IAPP	Nanostring
ICA1	Nanostring
ICAM1	Nanostring
ICAM2	Nanostring
ICAM3	Nanostring
ICOS	Nanostring
ICOSLG	Nanostring
IDO1	Nanostring
IER3	Nanostring

IFI27	Nanostring
IFI44L	Nanostring
IFI6	Nanostring
IFIH1	Nanostring
IFIT1	Nanostring
IFIT3	Nanostring
IFITM1	Nanostring
IFITM3	Nanostring
IFNA1/13	Nanostring
IFNAR1	Nanostring
IFNAR2	Nanostring
IFNG	Nanostring
IFNGR1	Nanostring
IFNGR2	Nanostring
IFNL2/3	Nanostring
IGF1	Nanostring
IGF1R	Nanostring
IGF2	Nanostring
IGF2R	Nanostring
IGFBP3	Nanostring
IGFBP5	Nanostring
IGFBP6	Nanostring
IGFBP7	Nanostring
IGHA1	Nanostring
IGHD	Nanostring
IGHG1	Nanostring
IGHG2	Nanostring
IGHM	Nanostring
IGKC	Nanostring
IKZF3	Nanostring
IL10	Nanostring
IL10RA	Nanostring
IL10RB	Nanostring
IL11	Nanostring
IL11RA	Nanostring
IL12A	Nanostring
IL12B	Nanostring
IL12RB1	Nanostring
IL12RB2	Nanostring
IL13RA1	Nanostring
IL15	Nanostring
IL15RA	Nanostring
IL16	Nanostring
IL17A	Nanostring
IL17B	Nanostring
IL17D	Nanostring
IL17RA	Nanostring
IL17RB	Nanostring
IL17RE	Nanostring
IL18	Nanostring
IL18R1	Nanostring
IL1A	Nanostring
IL1B	Nanostring
IL1R1	Nanostring
IL1R2	Nanostring
IL1RAP	Nanostring
IL1RL1	Nanostring
IL1RN	Nanostring
IL2	Nanostring
IL20	Nanostring
IL20RA	Nanostring
IL22RA1	Nanostring
IL23A	Nanostring
IL24	Nanostring
IL27RA	Nanostring
IL2RA	Nanostring
IL2RB	Nanostring
IL2RG	Nanostring
IL32	Nanostring
IL33	Nanostring
IL34	Nanostring
IL36G	Nanostring
IL3RA	Nanostring
IL4R	Nanostring
IL6	Nanostring
IL6R	Nanostring
IL6ST	Nanostring
IL7	Nanostring
IL7R	Nanostring
INHA	Nanostring
INHBA	Nanostring
INHBB	Nanostring
INS	Nanostring
INSIG1	Nanostring
INSR	Nanostring
IRF3	Nanostring
IRF4	Nanostring
ISG15	Nanostring
ITGA1	Nanostring
ITGA2	Nanostring
ITGA3	Nanostring

ITGA5	Nanostring
ITGA6	Nanostring
ITGA8	Nanostring
ITGA9	Nanostring
ITGAE	Nanostring
ITGAL	Nanostring
ITGAM	Nanostring
ITGAV	Nanostring
ITGAX	Nanostring
ITGB1	Nanostring
ITGB2	Nanostring
ITGB4	Nanostring
ITGB5	Nanostring
ITGB6	Nanostring
ITGB8	Nanostring
ITK	Nanostring
ITM2A	Nanostring
ITM2B	Nanostring
JAG1	Nanostring
JAK1	Nanostring
JAK2	Nanostring
JCHAIN	Nanostring
JUN	Nanostring
JUNB	Nanostring
KDR	Nanostring
KIT	Nanostring
KITLG	Nanostring
KLF2	Nanostring
KLK3	Nanostring
KLRB1	Nanostring
KLRF1	Nanostring
KLRK1	Nanostring
KRAS	Nanostring
KRT1	Nanostring
KRT10	Nanostring
KRT13	Nanostring
KRT14	Nanostring
KRT15	Nanostring
KRT16	Nanostring
KRT17	Nanostring
KRT18	Nanostring
KRT19	Nanostring
KRT20	Nanostring
KRT23	Nanostring
KRT4	Nanostring
KRT5	Nanostring
KRT6A/B/C	Nanostring
KRT7	Nanostring
KRT8	Nanostring
KRT80	Nanostring
KRT86	Nanostring
LAG3	Nanostring
LAIR1	Nanostring
LAMA4	Nanostring
LAMP2	Nanostring
LAMP3	Nanostring
LCN2	Nanostring
LDB2	Nanostring
LDHA	Nanostring
LDLR	Nanostring
LEFTY1	Nanostring
LEP	Nanostring
LGALS1	Nanostring
LGALS3	Nanostring
LGALS3BP	Nanostring
LGALS9	Nanostring
LGR5	Nanostring
LIF	Nanostring
LIFR	Nanostring
LINC01781	Nanostring
LINC01857	Nanostring
LINC02446	Nanostring
LMNA	Nanostring
LPAR5	Nanostring
LTB	Nanostring
LTBR	Nanostring
LTF	Nanostring
LUM	Nanostring
LY6D	Nanostring
LY75	Nanostring
LYN	Nanostring
LYVE1	Nanostring
LYZ	Nanostring
MAF	Nanostring
MALAT1	Nanostring
MAML2	Nanostring
MAP1LC3B/2	Nanostring
MAP2K1	Nanostring
MAPK13	Nanostring
MAPK14	Nanostring
MARCKSL1	Nanostring

MARCO	Nanostring
MB	Nanostring
MECOM	Nanostring
MEG3	Nanostring
MERTK	Nanostring
MET	Nanostring
MFAP5	Nanostring
MGP	Nanostring
MHC I	Nanostring
MIF	Nanostring
MIR4435-2HG	Nanostring
MKI67	Nanostring
MMP1	Nanostring
MMP12	Nanostring
MMP14	Nanostring
MMP19	Nanostring
MMP2	Nanostring
MMP7	Nanostring
MMP9	Nanostring
MPO	Nanostring
MRC1	Nanostring
MRC2	Nanostring
MS4A1	Nanostring
MS4A4A	Nanostring
MS4A6A	Nanostring
MSMB	Nanostring
MSR1	Nanostring
MST1R	Nanostring
MT1X	Nanostring
MT2A	Nanostring
MTOR	Nanostring
MX1	Nanostring
MXRA8	Nanostring
MYC	Nanostring
MYH11	Nanostring
MYH6	Nanostring
MYL12A	Nanostring
MYL4	Nanostring
MYL7	Nanostring
MYL9	Nanostring
MZB1	Nanostring
MZT2A/B	Nanostring
NACA	Nanostring
NANOG	Nanostring
NCAM1	Nanostring
NCR1	Nanostring
NDRG1	Nanostring
NDUFA4L2	Nanostring
NEAT1	Nanostring
NELL2	Nanostring
NFKB1	Nanostring
NFKBIA	Nanostring
NGFR	Nanostring
NKG7	Nanostring
NLRC4	Nanostring
NLRC5	Nanostring
NLRP1	Nanostring
NLRP2	Nanostring
NLRP3	Nanostring
NOD2	Nanostring
NOSIP	Nanostring
NOTCH1	Nanostring
NOTCH2	Nanostring
NOTCH3	Nanostring
NPPC	Nanostring
NPR1	Nanostring
NPR2	Nanostring
NPR3	Nanostring
NR1H2	Nanostring
NR1H3	Nanostring
NR2F2	Nanostring
NR3C1	Nanostring
NRG1	Nanostring
NRXN1	Nanostring
NRXN3	Nanostring
NTRK2	Nanostring
NUPR1	Nanostring
NUSAP1	Nanostring
OAS1	Nanostring
OAS2	Nanostring
OAS3	Nanostring
OASL	Nanostring
OLFM4	Nanostring
OLR1	Nanostring
OSM	Nanostring
OSMR	Nanostring
P2RX5	Nanostring
PARP1	Nanostring
PCNA	Nanostring
PDCD1	Nanostring
PDCD1LG2	Nanostring

PDGFA	Nanostring
PDGFB	Nanostring
PDGFC	Nanostring
PDGFD	Nanostring
PDGFRA	Nanostring
PDGFRB	Nanostring
PDS5A	Nanostring
PECAM1	Nanostring
PF4/V1	Nanostring
PFN1	Nanostring
PGF	Nanostring
PGK1	Nanostring
PGR	Nanostring
PHLDA2	Nanostring
PIGR	Nanostring
PLAC8	Nanostring
PLAC9	Nanostring
PLCG1	Nanostring
PLD3	Nanostring
PNOC	Nanostring
POU5F1	Nanostring
PPARA	Nanostring
PPARD	Nanostring
PPARG	Nanostring
PPIA	Nanostring
PRF1	Nanostring
PROX1	Nanostring
PRSS2	Nanostring
PRTN3	Nanostring
PSAP	Nanostring
PSCA	Nanostring
PSD3	Nanostring
PTEN	Nanostring
PTGDR2	Nanostring
PTGDS	Nanostring
PTGES	Nanostring
PTGES2	Nanostring
PTGES3	Nanostring
PTGIS	Nanostring
PTGS1	Nanostring
PTGS2	Nanostring
PTK2	Nanostring
PTK6	Nanostring
PTPRC	Nanostring
PTPRCAP	Nanostring
PTTG1	Nanostring
PXDN	Nanostring
QRFPR	Nanostring
RAC1	Nanostring
RAC2	Nanostring
RACK1	Nanostring
RAG1	Nanostring
RAMP1	Nanostring
RAMP2	Nanostring
RAMP3	Nanostring
RARA	Nanostring
RARB	Nanostring
RARG	Nanostring
RARRES1	Nanostring
RARRES2	Nanostring
RB1	Nanostring
RBM47	Nanostring
RBPJ	Nanostring
REG1A	Nanostring
RELA	Nanostring
RELT	Nanostring
RGCC	Nanostring
RGS1	Nanostring
RGS13	Nanostring
RGS2	Nanostring
RGS5	Nanostring
RNF43	Nanostring
ROR1	Nanostring
RORA	Nanostring
RPL21	Nanostring
RPL22	Nanostring
RPL32	Nanostring
RPL34	Nanostring
RPL37	Nanostring
RPS4Y1	Nanostring
RSPO3	Nanostring
RUNX3	Nanostring
RXRA	Nanostring
RXRB	Nanostring
RYK	Nanostring
RYR2	Nanostring
S100A10	Nanostring
S100A2	Nanostring
S100A4	Nanostring
S100A6	Nanostring
S100A8	Nanostring

S100A9	Nanostring
S100B	Nanostring
S100P	Nanostring
SAA1/2	Nanostring
SAT1	Nanostring
SCG5	Nanostring
SCGB3A1	Nanostring
SEC23A	Nanostring
SEC61G	Nanostring
SELENOP	Nanostring
SELL	Nanostring
SELPLG	Nanostring
SERPINA1	Nanostring
SERPINA3	Nanostring
SERPINB5	Nanostring
SERPINH1	Nanostring
SFN	Nanostring
SH3BGRL3	Nanostring
SIGIRR	Nanostring
SLA	Nanostring
SLC2A1	Nanostring
SLC40A1	Nanostring
SLCO2B1	Nanostring
SLPI	Nanostring
SMAD2	Nanostring
SMAD3	Nanostring
SMAD4	Nanostring
SMARCB1	Nanostring
SMO	Nanostring
SNAI1	Nanostring
SNAI2	Nanostring
SOD1	Nanostring
SOD2	Nanostring
SORBS1	Nanostring
SOSTDC1	Nanostring
SOX2	Nanostring
SOX4	Nanostring
SOX9	Nanostring
SPARCL1	Nanostring
SPINK1	Nanostring
SPOCK2	Nanostring
SPP1	Nanostring
SPRY2	Nanostring
SPRY4	Nanostring
SQLE	Nanostring
SQSTM1	Nanostring
SRC	Nanostring
SREBF1	Nanostring
SRGN	Nanostring
SRSF2	Nanostring
SST	Nanostring
ST6GAL1	Nanostring
ST6GALNAC3	Nanostring
STAT1	Nanostring
STAT3	Nanostring
STAT4	Nanostring
STAT5A	Nanostring
STAT5B	Nanostring
STAT6	Nanostring
STMN1	Nanostring
SYK	Nanostring
TACSTD2	Nanostring
TAGLN	Nanostring
TAP1	Nanostring
TAP2	Nanostring
TBX21	Nanostring
TCAP	Nanostring
TCF7	Nanostring
TCL1A	Nanostring
TEK	Nanostring
TFEB	Nanostring
TGFB1	Nanostring
TGFB2	Nanostring
TGFB3	Nanostring
TGFBI	Nanostring
TGFBR1	Nanostring
TGFBR2	Nanostring
THBS1	Nanostring
THBS2	Nanostring
THSD4	Nanostring
TIE1	Nanostring
TIGIT	Nanostring
TIMP1	Nanostring
TLR1	Nanostring
TLR2	Nanostring
TLR3	Nanostring
TLR4	Nanostring
TLR5	Nanostring
TLR7	Nanostring
TLR8	Nanostring
TM4SF1	Nanostring

TNF	Nanostring
TNFAIP6	Nanostring
TNFRSF10A	Nanostring
TNFRSF10B	Nanostring
TNFRSF10D	Nanostring
TNFRSF11A	Nanostring
TNFRSF11B	Nanostring
TNFRSF12A	Nanostring
TNFRSF13B	Nanostring
TNFRSF14	Nanostring
TNFRSF17	Nanostring
TNFRSF18	Nanostring
TNFRSF19	Nanostring
TNFRSF1A	Nanostring
TNFRSF1B	Nanostring
TNFRSF21	Nanostring
TNFRSF4	Nanostring
TNFRSF9	Nanostring
TNFSF10	Nanostring
TNFSF12	Nanostring
TNFSF13B	Nanostring
TNFSF14	Nanostring
TNFSF15	Nanostring
TNFSF4	Nanostring
TNFSF8	Nanostring
TNFSF9	Nanostring
TNNC1	Nanostring
TNNT2	Nanostring
TNXA/B	Nanostring
TOP2A	Nanostring
TOX	Nanostring
TP53	Nanostring
TPI1	Nanostring
TPM1	Nanostring
TPM2	Nanostring
TPSAB1/B2	Nanostring
TPT1	Nanostring
TSC22D1	Nanostring
TSHZ2	Nanostring
TTN	Nanostring
TTR	Nanostring
TUBB	Nanostring
TUBB4B	Nanostring
TWIST1	Nanostring
TWIST2	Nanostring
TXK	Nanostring
TYK2	Nanostring
TYMS	Nanostring
TYROBP	Nanostring
UBA52	Nanostring
UBE2C	Nanostring
UPK3A	Nanostring
VCAM1	Nanostring
VCAN	Nanostring
VEGFA	Nanostring
VEGFB	Nanostring
VEGFC	Nanostring
VEGFD	Nanostring
VHL	Nanostring
VIM	Nanostring
VPREB3	Nanostring
VSIR	Nanostring
VTN	Nanostring
VWA1	Nanostring
VWF	Nanostring
WIF1	Nanostring
WNT10B	Nanostring
WNT11	Nanostring
WNT3	Nanostring
WNT5A	Nanostring
WNT5B	Nanostring
WNT7A	Nanostring
WNT7B	Nanostring
WNT9A	Nanostring
XBP1	Nanostring
XCL1/2	Nanostring
XKR4	Nanostring
YBX3	Nanostring
YES1	Nanostring
ZBTB16	Nanostring
ZFP36	Nanostring

Supplementary Table 3. Antibodies and reagents.

Assay type	Cell subsets	Gating Strategy
CyTOF supervised	Epithelial	EpCAM^{+} CD45^{-}
	EpCAM^{+} CD15^{+}	EpCAM^{+} CD15^{+}
	EpCAM^{+} ICOS^{+}	EpCAM^{+} ICOS^{+}
	T	CD45^{+} EpCAM^{-} CD3^{+} gdTCR^{-}
	gd T	CD45^{+} EpCAM^{-} CD3^{+} gdTCR^{+}
	CD8^{+} T	CD45^{+} EpCAM^{-} CD3^{+} gdTCR^{-} CD4^{-} CD8a^{+}
	Naïve CD8^{+}	CD45^{+} CD3^{+} CD4^{-} CD8a^{+} CD45RA^{+} CCR7^{+}
	CD8$^{+}_{TCM}$	CD45^{+} CD3^{+} CD4^{-} CD8a^{+} CD45RA^{-} CCR7^{+}
	CD8$^{+}_{TEM}$	CD45^{+} CD3^{+} CD4^{-} CD8a^{+} CD45RA^{-} CCR7^{-}
	CD8$^{+}_{TEMRA}$	CD45^{+} CD3^{+} CD4^{-} CD8a^{+} CD45RA^{+} CCR7^{-}
	CD4^{+} T	CD45^{+} EpCAM^{-} CD3^{+} gdTCR^{-} CD4^{+} CD8a^{-}
	Naïve CD4^{+}	CD45^{+} CD3^{+} CD4^{+} CD8a^{-}CD45RA^{+} CCR7^{+}
	CD4$^{+}_{TCM}$	CD45^{+} CD3^{+} CD4^{+} CD8a^{-}CD45RA^{-} CCR7^{+}
	CD4$^{+}_{TEM}$	CD45^{+} CD3^{+} CD4^{+} CD8a^{-} CD45RA^{-} CCR7^{-}
	CD4$^{+}_{TEMRA}$	CD45^{+} CD3^{+} CD4^{+} CD8a^{-}CD45RA^{+} CCR7^{-}
	T_{reg}	CD45^{+} CD3^{+} CD4^{+} CD8a^{-} FOXP3^{+} CTLA-4^{+} CD127^{-}
	NKT	CD45^{+} EpCAM^{-} CD3^{+} CD16^{+} CD56^{+}
	CD16^{-} NK	CD45^{+} EpCAM^{-} CD3^{-} gdTCR^{-} HLA-DR^{+} CD19^{-} CD14^{-} CD7^{+} CD56^{hi} CD16^{-}
	CD16^{+} NK	CD45^{+} EpCAM^{-} CD3^{-} gdTCR^{-} HLA-DR^{+} CD19^{-} CD14^{-} CD7^{+} CD56^{dim} CD16^{+}
	Granulocytes	CD45^{+} CD3^{-} CD19^{-} CD15^{+}
	B	CD45^{+} EpCAM^{-} CD3^{-} gdTCR^{-} HLA-DR^{+} CD7^{-} CD19^{+}
	Plasma	CD45^{+} EpCAM^{-} CD3^{-} HLA-DR^{-} CD7^{-} CD31^{-} CD19^{-} CD38^{+}
	cDC1	CD45^{+} CD3^{-} CD7^{-} HLA-DR^{+} CD19^{-} CD14^{-} CD11c^{+} CD1c^{-} CD141^{+}
	cDC2	CD45^{+} CD3^{-} CD7^{-} HLA-DR^{+} CD19^{-} CD14^{-} CD11c^{+} CD1c^{+} CD141^{-}
	cDC2b	CD45^{+} CD3^{-} CD7^{-} HLA-DR^{+} CD19^{-} CD14^{+} CD11c^{+} CD1c^{+}
	pDC	CD45^{+} CD3^{-} CD7^{-} HLA-DR^{+} CD19^{-} CD14^{-} CD123^{+} CD11c^{-} CD1c^{+}
	Classical Monocytes	CD45^{+} CD3^{-} CD7^{-} HLA-DR^{+} CD19^{-} CD14^{+} CD16^{-} CD163^{-}
	Non-Classical Monocytes	CD45^{+} CD3^{-} CD7^{-} HLA-DR^{+}CD19^{-} CD14^{-} CD16^{+}
	Macrophages	CD45^{+} CD3^{-} CD7^{-} HLA-DR^{+}CD19^{-} CD14^{+} CD16^{-} CD163^{+}
CyTOF unsupervised blood	1_ CD4^{naïve}	CD45^{+} CD3^{+} CD4^{+} CD8a^{-} CD45RA^{+} CCR7^{+}
	2,5_ CD4^{TCM}	CD45^{+} CD3^{+} CD4^{+} CD8a^{-} CD45RA^{-} CCR7^{+}
	3,6,9,_ CD4^{TEM}	CD45^{+} CD3^{+} CD4^{+} CD8a^{-} CD45RA^{-} CCR7^{-}
	4_ CD8^{naïve}	CD45^{+} CD3^{+} CD8a^{+} CD4^{-} CD45RA^{+} CCR7^{+}
	7,10_ CD8^{TEM}	CD45^{+} CD3^{+} CD8a^{+} CD4^{-} CD45RA^{-} CCR7^{-}
	8_gd T	CD45^{+} CD3^{+} gdTCR^{+}
	11_CD8 T CD38^{+} HLA-DR^{+}	CD45^{+} CD8a^{+} HLA-DR^{+} CD38^{+}
	12_CD4 T CD38^{+} HLA-DR^{+}	CD45^{+} CD4^{+} HLA-DR^{+} CD38^{+}
	13_NK CD56^{high}	CD45^{+} CD7^{+} CD56^{hi} CD16^{hi}
	14_NK CD56^{low}	CD45^{+} CD7^{+} CD56^{low} CD16^{low}
	15_B	CD45^{+} HLA-DR^{+} CD7^{-} CD19^{+}
	16_Granulocyte	CD45^{+} CD16^{+} CD15^{+}
	17_mDC	CD45^{+} CD7^{-} HLA-DR^{+} CD14^{low} CD123^{-} CD11c^{+}
	18,20,21_Classical Monocytes	CD45^{+} CD7^{-} HLA-DR^{+} CD14^{+} CD16^{-} CD163^{-}
	19_Non classical Monocytes	CD45^{+} CD7^{-} HLA-DR^{+} CD14^{-} CD16^{+}
CyTOF unsupervised biopsy	1,4_Epithelial	EpCAM^{+}
	2,3_EpCAM^{+} CD15^{high}	EpCAM^{+} CD15^{hi}
	5,6,8_EpCAM^{+} ICOS^{high}	EpCAM^{+} ICOS^{hi}
	9,12_EpCAM^{+} HLA-DR^{+}	EpCAM^{+} HLA-DR^{+}
	11,17_Stromal S1	CD45^{-} CD31^{-} EpCAM^{-} CD56^{+}
	13_Stromal	CD45^{-} CD31^{-} EpCAM^{-}
	14_B	CD45^{+} HLA-DR^{+} CD19^{+}
	15_Endothelial	CD45^{-} HLA-DR^{+} EpCAM^{-} CD31^{+}
	16_CD4 T	CD45^{+} CD3^{+} CD4^{+} CD8a^{-}
	18_MNP-mDC and Macrophages	CD45^{+} CD7^{-} HLA-DR^{+} CD14^{low} CD16^{-} CD11c^{+} CD19^{-} CD163^{+}
	19_CD8 T	CD45^{+} CD3^{+} CD8a^{+} CD4^{-}
	20,21_Plasma	CD45^{+} EpCAM^{-} CD3^{-} HLA-DR^{-} CD7^{-} CD19^{-} CD38^{+}
	Epithelial	E-cadherin^{+} CD45^{-} CD31^{-}
	Epithelial CD68+	E-cadherin^{+} CD68^{+} CD3^{-} CD163^{-}
	Epithelial HLA-DR+	E-cadherin^{+} HLA-DR^{+} CD3^{-} CD163^{-}

CODEX supervised	Epithelial cycling	E-cadherin^{+} Ki67^{+}
	Endothelial	CD31^{+} E-cadherin^{-} CD45^{-}
	Stromal fibroblast CD44^{+}	Vimentin^{+} CD44^{+} CD45^{-} CD31^{-} CD20^{-} CD14^{-} CD3^{-} E-cadherin^{-} PDPN^{-}
	Stromal pericytes myofibroblats	Vimentin^{+} SMA^{+} Collagen IV^{+}
	Stromal fibroblast PDPN^{+}	Vimentin^{+} PDPN^{+} CD45^{-} CD31^{-} CD20^{-} CD14^{-} CD3^{-} E-cadherin^{-} CD44^{-}
	Hematopoietic	CD45^{+} E-cadherin^{-} CD31^{-}
	CD45^{+}	CD45+
	Monocyte	CD45^{+} HLA-DR^{+} CD3^{-} CD163^{-} CD20^{-} CD14^{+} CD68^{+}
	Macrophages	CD45^{+} HLA-DR^{+} CD3^{-} CD163^{+}
	B	CD45^{+} HLA-DR^{+} CD20^{+} CD3^{-} E-cadherin^{-} CD163^{-}
	T	CD45^{+} CD3^{+} E-cadherin^{-}
	CD4^{+} T	CD45^{+} CD3^{+} CD4^{+} CD8^{-}
	CD8^{+} T	CD45^{+} CD3^{+} CD4^{-} CD8^{+}
	Memory CD4^{+} T	CD45^{+} CD8^{-} CD3^{+} CD4^{+} CD45RO^{+}
	Memory CD8^{+} T	CD45^{+} CD4^{-} CD3^{+} CD8^{+} CD45RO^{+}
CODEX unsupervised	1_B	CD45^{+} HLA-DR^{+} CD19^{+} CD20^{+}
	2_Endothelial	E-cadherin- CD45^{-} CD31^{+}
	3_Epithelial	E-cadherin^{+} PCK^{+} CD45^{-} CD31^{-}
	4_Epithelial CD68^{+}	E-cadherin^{+} PCK^{+} CD68^{+}
	5_Epithelial cycling	E-cadherin^{+} PCK+ Ki67+
	6_Macrophages	CD45^{+} HLA-DR^{+} CD163^{+}
	7_Memory T	CD45^{+} CD3^{+} CD45RO^{+}
	8_Stromal fibroblast CD44^{+}	Vimentin^{+} CD44^{+} CD45^{-} CD3^{-} E-cadherin^{-}
	9_Stromal pericytes myofibroblast	Vimentin^{+} SMA^{+} Collagen IV^{+}
	10_Stromal fibroblast PDPN^{+}	Vimentin^{+} PDPN^{+} CD45^{-} CD3^{-} E-cadherin^{-}
	11_T CD45RO^{-}	CD45^{+} CD3^{+} CD45RO^{-}
MIBI supervised	Hematopoietic	CD45^{+} EpCAM^{-} CD31^{-}
	Epithelial	EpCAM^{+} CD45^{-} CD31^{-}
	Epithelial HLA-DR+	EpCAM^{+} HLA-DR^{+}
	Endothelial	CD31^{+} EpCAM^{-} CD45^{-}
	Stromal	Vimentin^{+} EpCAM^{-} CD45^{-} CD31^{-}
	T	CD45^{+} CD3^{+}
	CD4^{+} T	CD45^{+} CD3^{+} CD4^{+} CD8a^{-}
	CD8^{+} T	CD45^{+} CD3^{+} CD4^{-} CD8a^{+}
	Memory CD4^{+} T	CD45^{+} CD8^{-} CD3^{+} CD4^{+} CD45RO^{+}
	Memory CD8^{+} T	CD45^{+} CD4^{-} CD3^{+} CD8^{+} CD45RO^{+}
	Neutrophils	CD45^{+} EpCAM^{-} CD3^{-} CD20^{-} CD16^{+} CD56^{-} HLA-DR^{-}
	B	CD45^{+} CD3^{-} HLA-DR^{+} CD20^{+}
	Plasma	CD45^{+} CD3^{-} CD20^{-} CD138^{+}
	mDC	EpCAM^{-} CD45^{+} CD3^{-} CD20^{-} CD11c^{+}
	Mast	EpCAM^{-} CD45^{+} CD3^{-} CD20^{-} CD117^{+}
	Monocytes	HLA-DR^{+} CD163^{-} CD3^{-} CD20^{-} CD14^{+} CD68^{+}
	Macrophages	CD163^{+} CD68^{+} CD3^{-}
	Macrophages CD103^{+}	CD163^{+} CD3^{-} CD68^{+} CD103^{+}
	Fibroblasts	CD45^{-} EpCAM^{-} CD31^{-} Vimentin^{+} CD56^{-}
	Myofibroblasts	CD45^{-} EpCAM^{-} CD31^{-} Vimentin^{+} SMA^{+} CD56^{-}

Supplementary Table 4. Cell subset phenotypes for supervised and unsupervised analyses for each multi-omics method.

Cell type	Gene list	Numeric ID
MNP-pDC	*HLA-DRA*	8118548
	CD74	8115147
	HLA-DRB1	8125445
	CST3	8065403
	LYZ	7957023
	HLA-DPA1	8125556
	GZMB	7978366
	ITM2C	8048995
	SEC61B	8156838
	IRF1	8114010
	LILRA4	8039246
	APP	8069644
	C12orf75	7958253
	PPP1R14B	7949060
	JCHAIN	8100827
	TCF4	8023415
	UGCG	8157216
	ALOX5AP	7968344
	SERPINF1	8003667
	CCDC50	8084818
MNP-Monocytes	*HLA-DRA*	8118548
	CD74	8115147
	HLA-DRB1	8125445
	CST3	8065403
	LYZ	7957023
	HLA-DPA1	8125556
	S100A9	7905571
	S100A8	7920244
	TIMP1	8167185
	FCN1	8165011
	SERPINA1	7981068
	S100A4	7920271
	LILRA5	8039236
	S100A6	7920258
	TYMP	8077103
	VCAN	8106743
	SOD2	8130556
	CD44	7939341
	CD14	8114612
	EREG	8095728
	TSPO	8073612
MNP-Macrophage	*HLA-DRA*	8118548
	CD74	8115147
	HLA-DRB1	8125445
	CST3	8065403
	LYZ	7957023
	HLA-DPA1	8125556
	C1QA	7898793
	FTL	8030171
	C1QC	7898799
	C1QB	7898805
	ITM2B	7969003
	NPC2	7980146
	SLC40A1	8057677
	CCL3	8014369
	MS4A7	7940259
	CTSS	7919800
	FCGRT	8030374
	CCL4	8006602
MNP-mDC	*HLA-DRA*	8118548
	CD74	8115147
	HLA-DRB1	8125445
	CST3	8065403
	LYZ	7957023
	HLA-DPA1	8125556
	BASP1	8104601
	SLC38A1	7962516
	LTB	8124950
	CST7	8061416
	CD1C	7906348
	ACAP1	8004331
	DAPP1	8096602
	STK17A	8132503
	ADAM8	7937150
	FCER1A	7906443
	RHOF	7967202
	FSCN1	8131339
	ICAM3	8033987
	CD86	7896700
	CXCR4	8055465
	COL6A2	8069301
	CXCL14	8114249
	CALD1	8136347
	COL3A1	8046922

Stromal-Activated Fibroblast	*IGFBP7*	8100541
	C11orf96	7939492
	TIMP1	8167185
	MMP3	7951284
	MMP1	7951271
	AREG	8095736
	TMEM158	8086530
	TNFRSF11B	8152512
	ACSL4	8174474
	PLAU	7928429
	HGF	8140556
	STC1	8149825
	GJA1	8121749
	CD82	7939546
	ISG20	7985777
	NRG1	8145766
Stromal S2	*COL6A2*	8069301
	CXCL14	8114249
	CALD1	8136347
	COL3A1	8046922
	IGFBP7	8100541
	C11orf96	7939492
	F3	7917875
	POSTN	7971077
	ENHO	8160816
	PDGFRA	8095080
	SOX6	7946757
	PLAT	8150509
	NSG1	8093839
	HSD17B2	7997491
	DDHD1	7979223
	PDGFD	7951351
	AGT	7924987
	WNT5B	7953012
Endothelial	*IGFBP7*	8100541
	PLVAP	8035297
	SPARCL1	8101659
	IFITM3	7945371
	IGFBP4	8007100
	CAV1	8135594
	CCL21	8160889
	TFF3	8070567
	TFP1	8057599
	NUPR1	8000574
	MMRN1	8096415
	PROX1	7909681
	LYVE1	7946579
	AKAP12	8122807
	TBX1	8071276
	FABP4	8151532
	PPFIBP1	7954559
	RBP1	8091078
	EFEMP1	8052355
	NR2F1	8106923
	ACKR1	7906435
	NPC2	7980146
	CPE	8098204
	CLU	8149927
	MADCAM1	8023941
	DUSP23	7906469
	IL1R1	8043995
	ZNF385D	8085774
	KCTD12	7972055
	IL33	8154295
	ADGRG6	8122365
	LPCAT4	7987230
	SELP	7922200
	CTNNAL1	8163063
	TLL1	8098214
	GSN	8157582
	SLC9A3R2	7992463
	NOTCH4	8125383
	RGCC	7968789
	A2M	7960947
	MGLL	8090433
	VAMP5	8043203
	FLT1	7970763
	TSC22D1	7971350
	RBP7	7897522
	ADGRF5	8126798
	ADGRL4	7917182
	SRGN	7927964
	IL7R	8104901
	CD3E	7944179
	ARL4C	8059854
	KLRB1	7961059

CD4 T	*SPOCK2*	7934215
	KLF2	8026564
	SELL	7896687
	LEF1	8102232
	CCR7	8015031
	C1orf162	7903980
	LYPD3	8037301
	ACTN1	7979824
	TRABD2A	8053379
	GCNT4	8112668
	SLC40A1	8057677
	FHIT	8088458
	FAM117B	8047565
	S100A4	7920271
	JUN	7916609
	CCL5	8014316
	TNFAIP3	8122265
	ANXA1	8155849
	ANKRD28	8085628
	ID2	8040103
	EGR1	8108370
	GPR65	7976080
	LGALS3	7974461
	HOPX	8100507
T reg	*IL7R*	8104901
	CD3E	7944179
	ARL4C	8059854
	KLRB1	7961059
	SPOCK2	7934215
	CTLA4	8047692
	TIGIT	8081799
	MAF	8002969
	ARID5B	7927732
	BATF	7975793
	TBC1D4	7972021
	PHACTR2	8122426
	RNF19A	8152041
	TNFRSF4	7911413
	UCP2	7950307
	ICA1	8138202
	TNFRSF1B	7896721
	TNFRSF18	7911403
	DUSP4	8150076
	TOX2	8062782
Cytotoxic lymphocyte	*CCL5*	8014316
	NKG7	8038809
	GZMA	8105340
	IL32	7992828
	CCL4	8006602
	CD7	8019478
	GZMK	8105331
	ITGB2	8070826
	GZMH	7978360
	KLF2	8026564
	KLRG1	7953835
	KLRB1	7961059
	SPRY1	8097282
	CMC1	8078312
	SH2D1A	8169792
	HCST	8028104
	SH3BGRL3	7899153
	KLRD1	7953949
	CD3E	7944179
	CD3D	7952056
	CD69	7896693
	CD3G	7944185
	CD8A	8053584
B	*CD74*	8115147
	HLA-DRA	8118548
	MS4A1	7940287
	HLA-DRB1	8125445
	HLA-DPA1	8125556
	CD79A	8029136
	IGHM	7896697
	TCL1A	7981183
	FCER2	8033420
	YBX3	7961230
	KLF4	8163002
	KLF6	7931810
	GPR183	7972557
	TNFSF9	8025053
	CD27	7953333
	LDLRAD4	8020308
	ACTG1	8019183
	GAPDH	7953385
	BASP1	8104601

	PFN1	8011759
	HMGN1	8070389
	YBX1	7900585
	LCP1	7971461
	SLC25A5	8169640
	ARPC1B	8134552
	ANP32B	8156750
	ACTR3	8044686
	MARCKSL1	7914525
Plasma	*JCHAIN*	8100827
	MZB1	8114511
	SSR4	8170775
	CD79A	8029136
	IGHM	7896697
	CD44	7939341
	CD9	7953291
	CD48	7921667
	THY1	7952268
	LAG3	7953418
Epithelial	*LGALS4*	8036591
	PIGR	7923929
	KRT8	7963567
	C15orf48	7983478
	SH2D6	8043157
	TRPM5	7945742
	TLE4	8156060
	FABP1	8053654
	AQP8	7994252
	SLC26A3	8142171
	GUCA2A	7915404
	CEACAM7	8037053
	SLC26A2	8109194
	OTOP2	8009705
	BEST4	7915598
	CA4	8008900
	OLFM4	7969288
	LEFTY1	7924663
	PTGDR	7974363
	UBE2C	8063043
	HMGN2	7899187
	TOP2A	8014974
	HMGB2	8103728
	TUBA1B	7963046
Neutrophil	*S100A8*	7920244
	S100A9	7905571
	FCGR3A	7921868
	FCGR3B	7921873
	IL1R2	8043981
	GCA	8046003
	CSF3R	7914950
	S100P	8093950
	BASP1	8104601
	FPR1	8038899
	IFITM2	7937330
	ELANE	8024956
Pre-VDZ response signature	*GAS6*	7970329
	LEFTY1	7924663
	STMN1	7913864
	RPL37	8111814
	MARCKSL1	7914525
	NUPR1	8000574
	GSTP1	7941936
	LYZ	7957023
	SPINK1	8114964
	AGR2	8138381
	OLFM4	7969288
	JCHAIN	8100827
	REG1A	8042986
Pre-VDZ non-response signature	*MMP2*	7995681
	THBS1	7982597
	MMP1	7951271
	TCL1A	7981183
	COL21A1	8127201
	SPP1	8096301
	ITGA5	7963786
	HGF	8140556
	GZMK	8105331
	GZMH	7978360
	IFNG	7964787

Supplementary Table 5. Cell subset-specific gene signatures used for Gene Set Enrichment Analysis (GSEA). Numeric ID, Affymetrix numeric probe identifier corresponding to each gene.

Assay type	Patient ID	Specimen/imaged	Number of cells	% of hematopoietic	% of Stromal and Endothelial	% IEC	Multi-omic method
	HS1	Blood	899				
	HS2	Blood	3120				
	HS3	Blood	1699				
	HS4	Blood	1761				
	HS5	N/A	N/A				
	HS6	Blood	3474				
	HS7	Blood	2356				
	HS8	Blood	1111				
	HS9	Blood	1473				
	HS10	Blood	1669				
	HS11	Blood	1003				
	HS12	Blood	1565				
CITE-seq	HS1	Biopsy	9572	87.4	3.9	7.7	single cell transcriptomic and proteomic
	HS2	Biopsy	2648	85.7	5.4	8.3	
	HS3	Biopsy	8256	88.6	7.4	2.8	
	HS4	Biopsy	13662	77.8	10.5	10.7	
	HS5	Biopsy	6103	75.2	15.9	7.8	
	HS6	Biopsy	9036	70.2	22.2	6.3	
	HS7	Biopsy	9548	88.2	3.0	7.9	
	HS8	Biopsy	6076	79.1	15.8	4.3	
	HS9	Biopsy	4393	67.4	8.7	23.0	
	HS10	Biopsy	9192	80.6	5.7	13.3	
	HS11	Biopsy	7072	86.1	5.2	6.1	
	HS12	Biopsy	8342	78.9	10.8	9.6	
	HS1	Blood	57374				
	HS2	Blood	136673				
	HS3	Blood	167045				
	HS4	Blood	151381				
	HS5	N/A	N/A				
	HS6	Blood	75079				
	HS7	Blood	162640				
	HS8	Blood	121482				
	HS9	Blood	139270				
	HS10	Blood	85768				
	HS11	Blood	176299				
	HS12	Blood	117145				
CyTOF	HS1	Biopsy	82382	16.6	6.1	76.4	single cell proteomic
	HS2	Biopsy	83878	17.1	4.7	76.7	
	HS3	Biopsy	82480	32.0	8.9	57.7	
	HS4	Biopsy	57637	25.7	9.7	63.2	
	HS5	Biopsy	50659	26.7	9.4	62.9	
	HS6	Biopsy	30428	52.1	17.3	28.7	
	HS7	Biopsy	59387	34.6	5.6	57.2	
	HS8	Biopsy	41843	31.9	16.4	50.3	
	HS9	Biopsy	78302	18.0	7.0	74.1	
	HS10	Biopsy	25923	10.6	6.6	80.9	
	HS11	Biopsy	75650	33.8	7.4	57.7	
	HS12	Biopsy	15680	16.8	10.0	71.8	
	HS1	Core	8816	4.2	3.8	57.0	
	HS2	Core	10198	4.3	8.1	58.2	
	HS3	Core	6314	6.5	19.0	61.0	
	HS4	Core	5091	5.5	24.9	47.5	
	HS5	Core	8490	8.8	15.4	49.4	
	HS6	Core	8868	12.4	18.0	48.9	
Multiplexed RNA-ISH	HS7	Core	5779	7.2	10.3	55.4	FFPE spatial transcriptomic
	HS8	Core	5365	13.1	13.0	37.1	
	HS9	Core	10212	22.9	8.7	57.9	
	HS10	Core	6975	24.5	8.4	55.3	
	HS11	Core	13223	33.2	25.1	41.0	
	HS12	Core	14161	10.6	20.8	55.0	
	HS1	FOV	5131	30.6	14.3	55.2	
	HS2	FOV	6021	27.7	9.4	62.9	
	HS3	FOV	1353	50.2	11.2	38.6	
	HS4	FOV	4262	16.9	15.1	68.0	
	HS5	FOV	3774	32.8	16.4	50.8	
	HS6	FOV	N/A				
CosMx SMI 960-plex	HS7	FOV	4451	35.1	20.8	44.1	FFPE spatial transcriptomic
	HS8	FOV	4888	68.7	23.6	7.7	
	HS9	FOV	N/A				
	HS10	FOV	7765	35.7	24.0	40.3	
	HS11	FOV	6819	66.2	16.1	17.8	
	HS12	FOV	4319	33.9	27.0	39.1	
	HS1	Core	N/A				
	HS2	Core	10302	12.6	15.5	49.6	
	HS3	Core	3987	32.5	17.4	29.6	
	HS4	Core	3868	25.9	17.6	17.4	
	HS5	Core	3451	20.5	13.0	47.0	
	HS6	Core	N/A				
CODEX	HS7	Core	4487	13.2	19.8	20.1	FFPE spatial proteomic
	HS8	Core	5214	39.5	20.7	5.2	
	HS9	Core	3937	15.6	14.3	38.9	
	HS10	Core	11201	26.8	24.6	34.7	
	HS11	Core	12286	52.9	16.4	4.5	
	HS12	Core	9351	32.5	21.7	9.2	
	HS1	FOV	893	10.7	23.6	41.9	
	HS2	FOV	1455	14.4	10.3	45.4	
	HS3	FOV	1295	7.6	6.4	50.1	
	HS4	FOV	250	2.4	8.8	67.2	
	HS5	FOV	636	10.2	18.4	43.4	
	HS6	FOV	1630	29.9	16.0	13.4	
MIBI	HS7	FOV	349	11.0	24.2	42.5	FFPE spatial proteomic
	HS8	FOV	473	18.8	14.8	23.9	
	HS9	FOV	1090	11.4	12.1	40.2	
	HS10	FOV	1041	4.3	10.2	46.5	
	HS11	FOV	1732	27.7	11.8	24.2	
	HS12	FOV	1401	15.3	14.6	32.4	

Supplementary Table 6. Cell counts and percentages of hematopoietic, stromal and endothelial, and intestinal epithelial cell (IEC) subsets for each patient biospecimen and multi-omic method.

Gene	per FOV				FOVs as replicates				Biopsies as replicates				Include in gene set
**upregulated**	**baseMean**	**log2FoldChange**	**p-value**	**padj**	**baseMean**	**log2FoldChange**	**p-value**	**padj**	**baseMean**	**log2FoldChange**	**p-value**	**padj**	
KRT20	526.97688	1.04072071	0.06350846	0.41395902	1053.37471	1.829395189	0.01106976	0.29072685	1259.97142	1.759530615	0.03333355	0.58904936	
MMP2	214.781871	0.779233372	8.81E-05	0.00881248	408.395547	0.650608071	0.02130012	0.40684052	489.294934	0.704799783	0.04645766	0.64526877	Yes
CLDN4	759.82305	0.745929417	0.02768877	0.31826169	1483.20505	1.290087005	0.00365172	0.20246759	1776.28492	1.241179209	0.02295442	0.54436599	
TIMP1	276.204316	0.737634615	0.01112788	0.20232501	508.769011	0.667081209	0.12191453	0.76158335	602.95769	0.541749624	0.25696256	0.82642476	
PLAC8	935.934625	0.720560727	0.06581948	0.41395902	1848.43313	1.347448175	0.00690973	0.27583642	2314.7905	1.208635846	0.03799885	0.62106315	
THBS1	344.101653	0.672537259	0.00119803	0.04991792	647.142023	0.611143584	0.02401064	0.40684052	750.480415	0.745730473	0.00962025	0.40743756	Yes
JUN	453.634985	0.660664794	0.00672182	0.16894968	870.901615	0.956538051	0.00759029	0.28460002	1009.87437	1.096086482	NA	NA	
CEACAM1	294.859297	0.64336382	0.03858965	0.35695204	557.851558	1.107380773	0.00344384	0.20217367	673.708459	1.040242363	0.0190342	0.48659234	
MALAT1	5162.4939	0.636166653	0.19155692	0.58401499	10254.0368	1.040230675	0.08184969	0.69225416	12095.2239	1.062920598	0.11062494	0.73528713	
COL3A1	392.521296	0.633436878	0.0630143	0.41395902	739.85146	0.541377924	0.22738874	0.84271262	860.298456	0.433934687	0.42279269	0.95203628	
MMP1	207.858993	0.618646275	1.89E-07	0.00014484	385.571488	0.550571237	0.00041056	0.14987715	466.18652	0.56304112	0.00336529	0.23712057	Yes
FOS	352.469296	0.606341877	0.05283546	0.4033241	643.747795	0.869332204	0.05856023	0.60978778	714.941229	0.965660035	0.06011965	0.7039651	
SOD2	296.546792	0.598764496	0.01659691	0.26344298	564.82725	0.538386908	0.11886782	0.76044926	676.89997	0.477775366	0.20524567	0.79313927	
NEAT1	866.390363	0.572790109	0.122019	0.48996338	1726.88505	1.120800306	0.01984024	0.40684052	2078.89566	1.00077444	0.06888335	0.71379068	
MT2A	934.428365	0.571389758	0.00954193	0.19083854	1775.58372	0.495068787	0.09284265	0.70715244	2111.81567	0.565782914	0.11290352	0.74055794	
SQSTM1	474.161774	0.512804338	0.00661297	0.16894968	930.493327	0.848882786	0.00154193	0.15418283	1115.58897	0.813626862	0.01383623	0.47568006	
MMP9	255.898007	0.511030856	0.00172926	0.06404677	487.427814	0.380037984	0.06231327	0.61346199	583.529078	0.426210316	0.06352648	0.7039651	
ATF3	246.586056	0.505668525	0.00875501	0.18140874	459.530162	0.74200607	0.00798477	0.28460002	544.420923	0.785730082	0.01587284	0.48605613	
DUSP1	372.731731	0.486584761	0.00782812	0.18140874	678.460406	0.675381703	0.00409368	0.20427463	798.90527	0.704769132	0.00981204	0.40743756	
EZR	464.523069	0.436439785	0.13530634	0.50487439	906.187312	0.834827562	0.01687739	0.374303	1086.54509	0.747947535	0.07461127	0.71379068	
KRT16	609.755295	0.425423051	0.05657241	0.40890046	975.556748	0.445061083	0.14559645	0.78606569	1220.06216	0.428064554	0.22402087	0.80631339	
CDKN1A	378.607985	0.411588752	0.04890184	0.39601595	709.030108	0.777604211	0.00430737	0.20470263	865.725998	0.677779355	0.03367684	0.58904936	
CCL18	181.931178	0.399177236	0.00096405	0.04191509	333.952038	0.355215006	0.02214067	0.40684052	402.185354	0.365597111	0.05635691	0.70234803	
CRIP1	579.174589	0.377509857	0.06923619	0.41774829	1180.98787	0.665194598	0.04315395	0.55932003	1378.27245	0.741358386	0.06495967	0.7039651	
OAS1	262.689164	0.372964912	0.07135545	0.41825833	483.452543	0.728088809	0.00948109	0.29072685	588.271886	0.683797817	0.04216207	0.63067923	
CCL21	283.216261	0.34774063	0.11548864	0.48534695	543.974464	0.124593639	0.67556408	0.97295876	619.389899	0.311076366	0.18767891	0.79313927	
TCL1A	161.962139	0.324429339	4.83E-05	0.00604232	297.745557	0.286217762	0.00128782	0.15418283	358.914476	0.313275735	0.00066112	0.13182804	Yes
FKBP5	156.655595	0.319563737	0.01196562	0.20630378	288.313877	0.24083704	0.16907511	0.80350933	338.101572	0.241524611	0.21504822	0.80164351	
CXCL13	261.757772	0.304432077	0.02551588	0.31291833	486.537263	0.20020022	0.20258663	0.81386279	586.178145	0.259310993	0.15162784	0.77280279	
MYL12A	504.843393	0.303773039	0.22682194	0.61367037	993.525404	0.720716688	0.03159557	0.47063252	1160.67198	0.666088033	0.09287529	0.7263558	
OASL	258.014594	0.298766106	0.00554993	0.1541648	474.548174	0.456788851	0.00318848	0.20217367	577.31661	0.465282666	0.01485569	0.47777821	
ZFP36	466.041918	0.29221624	0.13335282	0.50487439	831.650665	0.475025375	0.05956942	0.60978778	988.611548	0.478474463	0.10912591	0.73528713	
RSPO3	65.2895623	0.290966063	0.04109392	0.35695204	122.094016	0.257943678	0.11869048	0.76044926	143.914325	0.308995191	0.09961188	0.7263558	
COL21A1	96.5411045	0.290774928	0.00083991	0.03999577	178.277478	0.319372181	0.00147965	0.15418283	213.722185	0.33860771	0.0022411	0.22083429	Yes
ARHGDIB	205.966227	0.287861641	0.04096562	0.35695204	382.444048	0.257510969	0.10132545	0.70715244	455.340504	0.218177936	0.16459059	0.78052343	
COL1A1	623.954591	0.284562889	0.33760458	0.71526394	1151.76314	0.251325271	0.49427984	0.95529411	1379.01178	0.06702276	0.88072016	0.97848848	
IL4R	220.007153	0.280776618	0.01092497	0.20231429	416.543543	0.348377681	0.01407256	0.32991373	495.252051	0.385286083	0.01478337	0.47777821	
SPP1	193.795024	0.274902042	0.00265226	0.08037161	352.005521	0.319550212	0.0020901	0.17382665	424.562732	0.317695632	0.00524	0.26121389	Yes
ITGA5	182.035166	0.273837899	0.00211066	0.07278133	340.852052	0.226084173	0.04506812	0.56934157	402.26067	0.25635497	0.03038689	0.5716175	Yes
IER3	401.465412	0.267372809	0.08111706	0.43161934	733.389792	0.512339802	0.01421472	0.32991373	878.336888	0.438805495	0.08685337	0.72408968	
KLF2	409.778611	0.259101857	0.03253334	0.338889	753.184873	0.297889683	0.02445935	0.40684052	892.492339	0.283721192	0.04879193	0.65261317	
CD163	117.519516	0.257539282	0.03652784	0.35695204	215.741815	0.235189381	0.10572635	0.72768895	256.577475	0.22120639	0.20230369	0.79313927	
CD52	178.342569	0.249309495	0.03080577	0.33484528	329.444325	0.171281424	0.22098906	0.84271262	388.811356	0.214139449	0.15035973	0.77280279	
VEGFC	116.304572	0.241700243	0.04845146	0.39601595	219.034269	0.196831987	0.19780647	0.81386279	263.045931	0.244462294	0.15182682	0.77280279	
MARCO	125.215062	0.23998011	0.00838667	0.18140874	231.83625	0.232269458	0.0238297	0.40684052	281.024054	0.265672579	0.02692013	0.55278634	
CCL3/L1/L3	418.653572	0.239374684	0.03389583	0.34587584	767.44468	0.262720995	0.08733366	0.69678309	930.295985	0.232282368	0.20422157	0.79313927	
CD22	92.3974621	0.23741245	0.07935439	0.43161934	171.503826	0.177683837	0.24582977	0.85483662	206.285795	0.191005006	0.25886286	0.82719957	
CHI3L1	218.435892	0.236505611	0.17854785	0.56041039	403.415375	0.233093136	0.33684141	0.92608189	491.699117	0.171029084	0.54958045	0.96029886	
HGF	128.808174	0.236183406	0.00028303	0.01818949	238.968156	0.247246483	0.00108059	0.15418283	287.728884	0.252449156	0.00200411	0.22083429	Yes
GZMK	177.279418	0.233999116	0.00092078	0.0418538	330.256396	0.246065621	0.00333458	0.20217367	397.674623	0.260074683	0.00454881	0.25195327	Yes
GZMH	181.54976	0.233366814	0.00033614	0.01977282	332.248125	0.215417286	0.00533193	0.23074301	398.359545	0.237092767	0.00374203	0.23712057	Yes
CSTB	349.213445	0.231526772	0.28965551	0.6758503	695.910767	0.618706889	0.04965394	0.5944744	812.204594	0.635499632	0.09632235	0.7263558	
KRT8	3308.93852	0.230305325	0.56872815	0.89607096	6431.03228	0.685827652	0.16679337	0.80350933	7524.25128	0.606972503	0.30805751	0.87752381	
COL6A3	260.388401	0.228403251	0.17222085	0.55376478	480.886454	0.1757595	0.33575148	0.9256353	565.933646	0.133127424	0.53617699	0.95668154	
IGHG1	11861.3389	0.227212704	0.72987109	0.94788453	21515.2779	0.226515772	0.81470406	0.98267871	28446.8984	-0.222486389	0.83419359	0.97848848	
P2RX5	212.30091	0.225002807	0.03911167	0.35695204	387.684989	0.186952907	0.16030126	0.80350933	470.149445	0.158137544	0.30897581	0.87763215	
MS4A1	141.663715	0.224287822	0.11323991	0.48187198	266.691471	0.145828457	0.37003973	0.93483037	317.340453	0.200614537	0.28703487	0.84792809	
ADGRL2	156.091385	0.222648518	0.06134205	0.41301069	289.506618	0.260626292	0.01292383	0.32244956	347.203971	0.279900094	0.02292565	0.54436599	
FGR	135.270051	0.218239284	0.01179528	0.20630378	248.924379	0.197430655	0.03606925	0.52169727	297.93001	0.218763278	0.04238266	0.63067923	
MMP14	223.439612	0.215868778	0.0230019	0.31251486	412.802454	0.170848858	0.14136747	0.78606569	486.266457	0.166358881	0.22042274	0.80164351	
LINC01781	161.499891	0.213090821	0.05752892	0.41092087	300.553731	0.33227904	0.01079723	0.29072685	360.094115	0.332373888	0.027883	0.5539472	
IFNG	132.922106	0.209627524	0.00083159	0.03999577	239.056038	0.201291538	0.01056766	0.29072685	287.138969	0.19944648	0.01204569	0.46190573	Yes
**downregulated**	**baseMean**	**log2FoldChange**	**p-value**	**padj**	**baseMean**	**log2FoldChange**	**p-value**	**padj**	**baseMean**	**log2FoldChange**	**p-value**	**padj**	
GAS6	221.185196	-0.422669228	4.1694E-05	0.00604232	398.898573	-0.433875128	0.00046858	0.14987715	459.862459	-0.4628411	0.00114575	0.1903854	Yes
RACK1	395.864527	-0.425699582	0.01391385	0.23582791	762.497029	-0.417365445	0.07758377	0.67046604	841.084843	-0.4667354	0.06624517	0.71017669	
LEFTY1	350.783571	-0.432882828	0.00076319	0.03999577	639.63574	-0.453902055	0.00477864	0.21677649	737.521281	-0.4766157	0.00854651	0.38731238	Yes
IFITM1	567.786594	-0.440511233	0.01166314	0.20630378	1027.34029	-0.393607065	0.09681441	0.70715244	1201.7823	-0.4863162	0.07691141	0.71664179	
SOD1	404.63738	-0.450326937	0.05683716	0.40890046	746.774687	-0.079510578	0.80252197	0.98267871	896.877388	-0.3422656	0.36383608	0.91987571	
STMN1	224.277107	-0.463971741	0.00222845	0.0742816	435.776705	-0.625848399	0.00387591	0.20358727	482.73007	-0.5502268	0.00380535	0.23712057	Yes
TAGLN	400.794087	-0.465372414	0.06054485	0.41301069	815.102372	-0.744071978	0.05150451	0.5944744	825.899445	-0.5244593	0.10191485	0.7263558	
ACTA2	208.410846	-0.467436601	0.0273665	0.31821516	409.307163	-0.674494762	0.02808689	0.45181565	422.322919	-0.4852252	0.04974784	0.65261317	
MZB1	457.395572	-0.469900866	0.11222921	0.48187198	787.510698	-0.391960885	0.30378442	0.90750876	951.358379	-0.6931322	0.07390732	0.71379068	
ENO1	674.764242	-0.47779548	0.03666936	0.35695204	1303.79448	-0.317671337	0.33289831	0.92286809	1509.60236	-0.4546723	0.25412041	0.8224827	
RPL37	1164.07257	-0.486565076	0.00184741	0.06597882	2254.52979	-0.567664791	0.00935087	0.29072685	2504.86947	-0.57782	0.01712215	0.48605613	Yes
CD24	893.47863	-0.496530225	0.09146679	0.44070361	1667.75422	-0.146530844	0.70889992	0.97295876	1924.15652	-0.2239604	0.62216763	0.96787016	
SERPINA1	316.905262	-0.505236225	0.02617137	0.31291833	572.590806	-0.337715433	0.27135833	0.87980019	665.767487	-0.5111801	0.06039109	0.7039651	
MARCKSL1	507.135416	-0.514949839	7.0838E-05	0.00787092	977.42141	-0.564491512	0.00078801	0.15418283	1092.40002	-0.5396293	0.00287948	0.22083429	Yes
CD74	1049.66347	-0.534416692	0.14557251	0.51682078	2047.43748	-0.634548304	0.18115517	0.80963857	2307.09604	-0.7836118	0.14481695	0.77280279	
NUPR1	414.135769	-0.542692124	0.00019168	0.0159736	750.641452	-0.496580349	0.00554893	0.23074301	864.005331	-0.529875	0.01254247	0.46314239	Yes
B3GNT7	326.536483	-0.560074569	0.0087201	0.18140874	631.550734	-0.67283412	0.02007386	0.40684052	674.458565	-0.5700646	0.04341242	0.63587154	
HSP90B1	244.268644	-0.5730694	0.01920323	0.28240045	455.244785	-0.546593252	0.07392523	0.6587266	506.42238	-0.6716737	0.02833632	0.5539472	
MIF	573.764636	-0.580882137	0.00731452	0.17415516	1081.86355	-0.6188022	0.0299933	0.45353505	1224.95284	-0.7397064	0.02508609	0.54779261	
GSTP1	401.351548	-0.593350286	0.00029103	0.01818949	753.491949	-0.491038079	0.02191854	0.40684052	845.178733	-0.5512753	0.02716803	0.55278634	Yes
XBP1	577.23872	-0.641598281	0.02444558	0.31251486	1025.23707	-0.56662493	0.14501216	0.78606569	1230.1811	-0.8573794	0.04169768	0.63067923	
TPM1	494.150118	-0.71891683	0.00028995	0.01818949	960.663094	-0.59210089	0.03973153	0.53583874	1046.16519	-0.5607185	0.08082574	0.72407637	
CXCL14	507.92397	-0.732029017	0.00987198	0.19266287	943.741862	-0.853766756	0.00842687	0.29000056	989.277974	-0.7483399	0.01755067	0.48605613	
FCGBP	583.972558	-0.766128118	0.02660682	0.31302146	1046.67936	-0.311774044	0.45566901	0.94573569	1176.92456	-0.5233057	0.26835162	0.84091614	
DMBT1	364.246918	-0.97960325	0.00128207	0.05128285	610.525892	-0.770871177	0.06588797	0.61971043	753.707716	-1.1509374	0.01302456	0.46376738	
LYZ	569.374814	-1.037726899	0.0003767	0.020928	1056.66259	-1.028751814	0.0023336	0.17914868	1186.49451	-1.1332978	0.00175111	0.22083429	Yes
IGKC	11391.2045	-1.081614737	0.04945073	0.39601595	19778.4443	-0.977617238	0.19049449	0.81386279	24238.2781	-1.4487737	0.07588948	0.71379068	
LCN2	373.12094	-1.118920726	0.00888903	0.18140874	613.075854	-0.902177296	0.12686875	0.76475159	774.995768	-1.4393452	0.02527428	0.54779261	
SPINK1	305.115064	-1.143197997	2.7571E-05	0.00551416	543.240618	-0.942639545	0.00792159	0.28460002	626.056659	-1.2421363	0.00186529	0.22083429	Yes
PIGR	2000.98599	-1.20820319	0.01495401	0.2451477	4094.95998	-0.793324568	0.20071777	0.81386279	4303.68823	-0.8914037	0.2312494	0.81018631	
AGR2	642.44532	-1.469522932	0.00010943	0.00994812	1253.02418	-1.492627964	0.00161069	0.15418283	1286.01099	-1.5726278	0.00282642	0.22083429	Yes
OLFM4	500.656969	-1.828631209	4.2451E-06	0.00141503	848.632798	-1.572535026	0.00330047	0.20217367	1024.66082	-2.0813306	0.0000552	0.02749459	Yes
IGHA1	19640.0736	-2.007187077	0.00024446	0.01818949	32960.2753	-1.838110589	0.01014572	0.29072685	39977.4172	-2.2911562	0.00415302	0.24356234	Yes
JCHAIN	1575.31083	-2.26824587	2.4837E-05	0.00551416	2810.2006	-2.161967137	0.00060071	0.14987715	3124.38258	-2.5234216	0.00024797	0.08240874	Yes
REG1A	807.633311	-2.727471055	2.8969E-07	0.00014484	1095.12586	-2.38186226	0.00169941	0.15418283	1327.72378	-2.9479076	0.00000482	0.0048067	Yes

Supplementary Table 7. Pseudobulk DE gene analysis of CosMx data comparing baseline biopsies prior to VDZ therapy in non-responders versus responders. In addition to q<0.1 after FDR-correction for all FOVs, DEseq2 comparisons were made treating FOVs and biopsies as replicates, with an unadjusted p<0.05 as an additional threshold for including in the gene sets.
