# Supplementary material for: Single-cell and spatial multi-omics highlight effects of anti-integrin therapy across cellular compartments in ulcerative colitis
Source: Nat Commun. 2024 Feb 19;15:1493. doi: 10.1038/s41467-024-45665-6 (PMC10876948; doi:10.1038/s41467-024-45665-6)
Supplement: Supplementary file 3 — Reporting Summary [file 41467_2024_45665_MOESM3_ESM.pdf]

nature portfolio

Corresponding author(s): Michael Kattah

Last updated by author(s): Dec 25, 2023

Reporting Summary

Nature Portfolio wishes to improve the reproducibility of the work that we publish. This form provides structure for consistency and transparency in reporting. For further information on Nature Portfolio policies, see our Editorial Policies and the Editorial Policy Checklist.

Statistics

For all statistical analyses, confirm that the following items are present in the figure legend, table legend, main text, or Methods section.

n/a	Confirmed	
☐	☒	The exact sample size (*n*) for each experimental group/condition, given as a discrete number and unit of measurement
☐	☒	A statement on whether measurements were taken from distinct samples or whether the same sample was measured repeatedly
☐	☒	The statistical test(s) used AND whether they are one- or two-sided<br>*Only common tests should be described solely by name; describe more complex techniques in the Methods section.*
☒	☐	A description of all covariates tested
☐	☒	A description of any assumptions or corrections, such as tests of normality and adjustment for multiple comparisons
☐	☒	A full description of the statistical parameters including central tendency (e.g. means) or other basic estimates (e.g. regression coefficient) AND variation (e.g. standard deviation) or associated estimates of uncertainty (e.g. confidence intervals)
☐	☒	For null hypothesis testing, the test statistic (e.g. *F*, *t*, *r*) with confidence intervals, effect sizes, degrees of freedom and *P* value noted<br>*Give P values as exact values whenever suitable.*
☒	☐	For Bayesian analysis, information on the choice of priors and Markov chain Monte Carlo settings
☐	☒	For hierarchical and complex designs, identification of the appropriate level for tests and full reporting of outcomes
☐	☒	Estimates of effect sizes (e.g. Cohen's *d*, Pearson's *r*), indicating how they were calculated

Our web collection on statistics for biologists contains articles on many of the points above.

Software and code

Policy information about availability of computer code

Data collection	Commercial Fluidigm CYTOF software was used for CyTOF data acquisition.
Data analysis	All code used in this study including R markdowns and Jupyter notebooks are available on the Ulcerative Colitis project GitHub repository (https://github.com/mkattah/UC_VDZ). Seurat v3, Cellranger 3.0.2, scanpy==1.9.1, anndata==0.8.0, squidpy==1.2.3, excellxgene==2.9.2, and MAST==1.20, were used for scRNA-seq and CosMx 960-plex RNA-ISH analysis. R-based Cytometry Clustering Optimization aNd Evaluation (Cyclone) pipeline was developed by the UCSF Data Science CoLab (https://github.com/UCSF-DSCOLAB/cyclone). Cyclone, FlowSOM 2.6.0 and FlowJo v10 were used for CyTOF and CODEX analysis. Cell segmentation and quantitation for 12-plex RNA-ISH, MIBI, and CODEX were performed using HALO version 3.4. Additional statistical analyses were performed in GraphPad Prism v9. Conda enivronments are specified in .yaml files provided at our GitHub link to facilitate reproducibility.

For manuscripts utilizing custom algorithms or software that are central to the research but not yet described in published literature, software must be made available to editors and reviewers. We strongly encourage code deposition in a community repository (e.g. GitHub). See the Nature Portfolio guidelines for submitting code & software for further information.

Data

Policy information about availability of data

All manuscripts must include a data availability statement. This statement should provide the following information, where applicable:

- Accession codes, unique identifiers, or web links for publicly available datasets
- A description of any restrictions on data availability
- For clinical datasets or third party data, please ensure that the statement adheres to our policy

The raw sequencing data generated in this study have been deposited on the dbGaP database under accession code phs003502.v1.p1. The raw sequencing data are available under controlled access for privacy concerns; access can be requested through dbGaP. Processed data are deposited as a GEO Super Series under accession code GSE250498 and are publicly available. All additional data generated in this study are provided in the Supplementary Information/Source Data file and on Figshare. Processed and annotated objects are saved in AnnData (h5ad) format78. These AnnData objects are accessible in Figshare (fresh versus cryopreserved scRNA-seq 10.6084/m9.figshare.21936240; blood scRNA-seq 10.6084/m9.figshare.21900948; biopsy scRNA-seq 10.6084/m9.figshare.21919425; biopsy CITE-seq 10.6084/m9.figshare.21919356; PBL CyTOF 10.6084/m9.figshare.21977834; biopsy CyTOF 10.6084/m9.figshare.21977798; secondary CyTOF PBL and biopsy analysis 10.6084/m9.figshare.23902065; CosMx 960-plex RNA-ISH 1st run 10.6084/m9.figshare.21919338; CosMx 1000-plex RNA-ISH 2nd longitudinal analysis 10.6084/m9.figshare.23896959). Publicly available microarray data (GSE73661) were downloaded from the NCBI gene expression omnibus. GRCh38 was used as the reference genome.

Research involving human participants, their data, or biological material

Policy information about studies with human participants or human data. See also policy information about sex, gender (identity/presentation), and sexual orientation and race, ethnicity and racism.

Reporting on sex and gender	Baseline demographic and clinical data for the study participants are provided in Supplementary Table 1. Demographic options were defined by the investigators and participants chose their classifications. We attempted to balance sex across disease and treatment groups. The study design is insufficient to determine the association of sex with disease or treatment response.
Reporting on race, ethnicity, or other socially relevant groupings	Baseline demographic and clinical data for the study participants are available through dbGaP. Demographic options were defined by the investigators and participants chose their classifications. We recruited consecutively eligible patients regardless of race or ethnicity across disease and treatment groups.
Population characteristics	Baseline demographic and clinical data for the study participants are provided in Supplementary Table 1. Demographic options were defined by the investigators and participants chose their classifications.
Recruitment	Patients undergoing colonoscopy or sigmoidoscopy for standard of care indications were screened for study eligibility. Eligible patients were recruited consecutively to minimize self-selection bias. Patients were compensated $50 for each sample collection event. For this study, HC patients were patients without known or suspected IBD undergoing elective colonoscopy or sigmoidoscopy for various indications (e.g. colorectal cancer screening). UC patients were eligible if they had an established diagnosis of UC by UCSF Gastroenterology faculty, had at least mild endoscopic disease activity (Mayo equal to or greater than 1), and if they were either on Vedolizumab or no biologic (5-ASA therapy) in the maintenace phase of treatment. Exclusion criteria included any contraindication to endoscopic forceps biopsy such as anticoagulation, dual antiplatelet agents that were not held prior to the procedure, or other high risk of bleeding. Subjects with anticipated procedural difficulty such as significant co-morbidities, prior difficult endoscopy, or any other planned high-risk endoscopic procedure were also excluded to minimize risk. All patients gave written informed consent and approval.
Ethics oversight	This study is approved by the Institutional Review Board of the University of California, San Francisco (19-27302).

Note that full information on the approval of the study protocol must also be provided in the manuscript.

Field-specific reporting

Please select the one below that is the best fit for your research. If you are not sure, read the appropriate sections before making your selection.

☒ Life sciences ☐ Behavioural & social sciences ☐ Ecological, evolutionary & environmental sciences

For a reference copy of the document with all sections, see nature.com/documents/nr-reporting-summary-flat.pdf

Life sciences study design

All studies must disclose on these points even when the disclosure is negative.

Sample size	The primary goal of this study was to determine the peripheral and mucosal cell subsets that contribute to ulcerative colitis (UC) and respond to Vedolizumab (VDZ). We wanted to compare healthy controls, patients with UC on aminosalicylates, and patients with UC on VDZ. Although we did not perform a detailed power calculation, we based our sample size on pilot scRNA-seq data showing that biopsy cryopreservation and batch processing facilitates inter-sample comparison,. We chose four patients per group as the minimum number to detect large effect sizes in relative cell subset frequency and differentially expressed genes. Our goal was to sequence >3000 cells per patient biopsy. For validation in longitudinal bulk transcriptomic data, all available pre- and post-treatment samples were included from GSE73661 to perform the indicated comparisons.

Data exclusions

Ribosomal and mitochondrial genes were not included in the final MAST output to prioritize differentially expressed genes that were most pertinent to the research question. For 12-plex RNA-ISH, MIBI, CODEX, and CosMx 960-plex RNA-ISH formalin-fixed, paraffin embedded (FFPE) analysis, low-quality cores or FOVs were excluded if they were grossly damaged or detached during processing, or if they had poor nuclei staining that precluded cell segmentation. For MIBI, FOVs that contained exclusively lymphoid aggregates, with no mucosal or submucosal cellular compartments, were also excluded.

Replication

Biopsy CITE-seq and blood scRNA-seq for all patients were performed on one day each, and both Right and Left colon biopsies were separately analyzed for each patient. The CITE-seq and scRNA-seq experiments were not replicated due to cost and limitations associated with small biospecimens. Additional research-only mucosal biopsies were obtained from two regions during endoscopic procedures for CITE-seq, CyTOF, bulk RNA-seq, and FFPE analysis. Given the number of biopsies obtained for the primary analysis, additional technical replicates could not be obtained without additional risk. CyTOF, 12-plex RNA-ISH, MIBI, CODEX, and 960-plex CosMx RNA-ISH on the same patient samples collected at the same time served as internal validation of major findings from CITE-seq analysis. The CyTOF, RNA-ISH, MIBI, CODEX, and CosMx experiments were not replicated due to cost and limitations associated with small biospecimens. However, the use of multiple, different methods on the same samples improved internal validation and served as a form of technical triangulation. For external validation, we then performed CyTOF and longitudinal CosMx spatial transcriptomics analysis in separate cohorts, and those analyses were not replicated due to cost and limitations associated with small biospecimens.

Randomization

This was a cross-sectional, case-control study design, so patients and corresponding biospecimens were not randomized. Patients were sequentially recruited with no additional pre-specified criteria other than disease and treatment status. Covariates and baseline demographics are highlighted in Supplementary Tables 1 & 2.

Blinding

All patient-derived biospecimens were de-identified with unique identifiers and researchers were blinded during all sample processing steps. Pathologists were blinded for histologic scoring. Samples were linked to disease and treatment status for data analysis. Blinding during data analysis was not employed due to the practical constraints of our case-control study design, which necessitated linking each sample to individual patients for accurate group assignment. However, to mitigate potential bias, we maintained blinding during the quantification of cell subset abundance in fields-of-view or regions-of-interest for our spatial multi-omics analyses.

Reporting for specific materials, systems and methods

We require information from authors about some types of materials, experimental systems and methods used in many studies. Here, indicate whether each material, system or method listed is relevant to your study. If you are not sure if a list item applies to your research, read the appropriate section before selecting a response.

Materials & experimental systems

n/a	Involved in the study
☐	☒ Antibodies
☒	☐ Eukaryotic cell lines
☒	☐ Palaeontology and archaeology
☒	☐ Animals and other organisms
☒	☐ Clinical data
☒	☐ Dual use research of concern
☒	☐ Plants

Methods

n/a	Involved in the study
☒	☐ ChIP-seq
☐	☒ Flow cytometry
☒	☐ MRI-based neuroimaging

Antibodies

Antibodies used

See Supplemental Table 3.

Markers for CyTOF:
CD45 (HI30) at 1.5 µg/ml, BioLegend (# 304002); EpCAM (9C4) at 1.5 µg/ml, Biolegend (# 324202); CD7 (M-T701) at 1.5 µg/ml, BD Bioscience (#555359); CD15 (W6D3) at 1.5 µg/ml, Biolegend (# 323002); CD3 (UCHT1) at 0.4 µg/ml, Biolegend (# 300402); CD19 (HIB19) at 0.4 µg/ml, Biolegend (# 302202); CD163 (GHI61) at 3 µg/ml, Biolegend (# 333602); CD4 (RPA-T4) at 0.4 µg/ml, Thermo (# 14-0049-82); CD8a (RPA-T8) at 0.4 µg/ml, Biolegend (# 301002); CD11c (BU15) at 0.4 µg/ml, Thermo (#MA-82142); CD14 (M5E2) at 1.5 µg/ml, Biolegend (# 301802); CD127 (A019D5) at 1.5 µg/ml, Biolegend (# 351302); CD123 (6H6) at 0.8 µg/ml, Thermo (# 14-1239-82); gdTCR (5A6.E9) at 0.4 µg/ml, ATCC (#HB-9772); CD45RA (HI100) at 1.5 µg/ml, Biolegend (# 304102); TIM3 (F38-2E2) at 3 µg/ml, Biolegend (#345002); TGIT (A15153G) at 1.5 µg/ml, Biolegend (# 372720); PD-L1 (29E.2A3) at 1.5 µg/ml, Biolegend (# 329702); Integrin a4 (9F10) at 1.5 µg/ml, Biolegend (# 304302); CD27 (O323) at 0.4 µg/ml, Biolegend (# 302802); CD137_4-1BB (4B4-1) at 1.5 µg/ml, Thermo (14-9056-82); Tbet (4B10) at 0.4 µg/ml, Thermo (# 14-5825-82); CTCLA-4 (14D3) at 0.4 µg/ml, Thermo (# 14-1529-82); FOXP3 (PCH101) at 0.8 µg/ml, Thermo (# 14-4776-82); CD31 (WM59) at 0.8 µg/ml, Biolegend (# 303102); Integrin b7 (FIB504) at 1.5 µg/ml, Biolegend (# 321202); CD141_BDCA3 (M80) at 0.8 µg/ml, Biolegend (# 344102); CCR7 (GO43H7) at 1.5 µg/ml, Biolegend (# 353202); Ki-67 (ki-67) at 0.8 µg/ml, Biolegend (# 350502); CD25 (M-A251) at 0.4 µg/ml, Biolegend (# 356102); CD1C_BDCA1 (L161) at 0.8 µg/ml, Biolegend (# 331502); CD38 (HIT2) at 0.4 µg/ml, Biolegend (# 303502); ICOS (C398.4A) at 0.8 µg/ml, Biolegend (# 313502); HLA-DR (L243) at 1.5 µg/ml, Biolegend (# 307665); PD-1 (EH12.2H7) at 1.5 µg/ml, Biolegend (# 329902); CD56 (NCAM16.2) at 0.8 µg/ml, BD Bioscience (# 559043); CD16 (3G8) at 0.4 µg/ml, Biolegend (# 302049).

Markers for MIBI: dsDNA (3519 DNA) at 0.2 µg/ml, Abcam (# ab27156); E-Cadherin (36) at 0.5 µg/ml, Abcam (# ab287971); Histone H3 (D1H2) at 1:100, Ionpath (# 711501-100); FoxP3-AF488 (236A/E7) at 2 µg/ml, BD Pharmigen (# 561181); anti-488 (polyclonal) at 3 µg/ml, Thermofisher (# A-11094); CD163 (EPR14643-36) at 0.6 µg/ml, Abcam (# ab215976); CD4 (EPR6855) at 1:100, Ionpath (# 714301-100); CD11c (EP1347Y) at 1:100, Ionpath (# 714401-100); CD14 (D7A27) at 0.6 µg/ml, CST (# 42878); CD16 (D1N9L) at 0.6

μg/ml, CST (# 72204); LAG3 (17B4) at 5 μg/ml Novus (# NBP1-97657); PD-1 (D4W2J) at 1:100, Ionpath (# 714801-100); PD-L1 (E1L3N) at 1:100, Ionpath (# 714902-100); Granzyme B (D6E9W) at 1:100, Ionpath (# 715002-100); CD56 (MRQ-42) at 1:100, Ionpath (#7155101-100); CD31 (EP3095) at 1:100, Ionpath (#715202-100); KI67 (D2H10) at 0.15 μg/ml, CST (# 44092); CD138 (EPR6454) at 5 μg/ml, Abcam (# ab226108); CD117 (YR145) at 1:100, Ionpath (#715501-100); CD68 (D4B9C) at 1:100, Ionpath (#715601-100); CD103 (EPR4166(2)) at 15 μg/ml, Abcam (# ab271889); CD8 (C8/144B) at 1:100, Ionpath (# 715801-100); CD3 (D7A6E) at 1:100, Ionpath (# 715903-100); Tbet (D6N8B) at 5 μg/ml, CST (# 27112); CD45RO (UCHL1) at 1:100, Ionpath (716101-100); TIM3 (EPR22241) at 1:100, Ionpath (# 716201-100); Vimentin (D21H3) at 1:100, Ionpath (# 716301-100); CD27 (EPR8569) at 5 μg/ml, Abcam (ab256583); Pan-Keratin (AE1/AE3) at 1:100, Ionpath (# 716501-100); aSMA (SP171) at 2.5 μg/ml, Abcam (# ab242395); CD20 (L26) at 1:100, Ionpath (# 716701-100); CD11b (D6X1N) at 0.5 μg/ml, Abcam (#ab187537); BDCA3-CD141 (E7Y9P) at 10 μg/ml, CST (#34149); CD21 (EP3093) at 1:100, Ionpath (# 717001-100); IDO1 (EPR20374) at 1:100, Ionpath (# 717101-100); HLA-DR (EPR3692) at 1:100, Ionpath (# 717201-100); EpCAM (D9S3P) at 0.5 μg/ml, CST (# 55725); CD45 (2B11/PD7/26) at 1:100, Ionpath (# 717501-100); HLA-Class 1 (EMR8-5) at 1:100, Ionpath (# 717602-100).

Markers for CODEX: HLA-A (EP1395Y) at 1:200, Abcam (# ab216653); CD34 (QBEND/10) at 1:150, Thermofisher (# MA1-10205); CD4 (EPR6885) at 1:200, Abcam (# ab133616); CD20 (L26) at 1:200, Thermofisher (# 14-0202-82); CD14 (EPR3653) at 1:500, Abcam (# ab133335); CD68 (KP1) at 1:200, Thermofisher (# MA5-13324); Vimentin (091D3) at 1:200, Biolegend (# 677802); CD8 (C8/144B) at 1:200, Biolegend (# 372908); CD11c (118/A5) at 1:200, Thermofisher (# 14-9761-82); CD31 (EP3095) at 1:100, Abcam (# ab134168); E-Cadherin (4A2C7) at 1:300, Thermofisher (# 33-4000); CD45 (D9M8I) at 1:300, CST (# 13917); SMA (1A4) at 1:200, Abcam (# ab7817); CD45RO (UCHL1) at 1:200, Biolegend (# 304202); CD3e (EP449E) at 1:200, Abcam (# ab52959); Pan-cytokeratin (AE1/AE3) at 1:200, Biolegend (# 914204); CD44 (156-3C11) at 1:400, CST (#3570); HLA-DR (EPR3692), at 1:200, Abcam (# ab92511); Granzyme B (D6E9W) at 1:50, CST (# 46890); Collagen IV (EPR209660) at 1:200, Abcam (# ab214417); Podoplanin (NC-08) at 1:200, Biolegend (# 337002); FOXP3 (259D/C7) AT 1:100, BD Pharmigen (# 560044); Ki67 (B56) at 1:300, BD Pharmigen (# 556003); CD163 (D6U1J) at 1:50, CST (#93498); CD19 (RM332) at 1:50, RevMab (# 31-1219-00); CD11b (EP1345Y) at 1:50, Abcam (# ab52478); CD21 (EP3093) at 1:400, Abcam (# ab75985); CD57 (HNK-1) at 1:100, Biolegend (# 359610).

Markers for CITE-seq:
NOTE dilutions are proprietary according BioLegen.
CD103 (Integrin aE) GACCTCATTGTGAAT A0145 Ber-ACT8 proprietary CD103 399907 BioLegend
CD35 ACTTCCGTCGATCTT A0167 E11 proprietary CD35 399907 BioLegend
CD25 TTTGTCCTGTACGCC A0085 BC96 proprietary CD25 399907 BioLegend
CD62L GTCCCTGCAACTTGA A0147 DREG-56 proprietary CD62L 399907 BioLegend
CD3 CTCATTGTAACTCCT A0034 UCHT1 proprietary CD3 399907 BioLegend
CD33 TAACTCAGGGCCTAT A0052 P67.6 proprietary CD33.1 399907 BioLegend
CD4 TGTTCCCGCTCAACT A0072 RPA-T4 proprietary CD4.2 399907 BioLegend
CD58 (LFA-3) GTTCCTATGGACGAC A0174 TS2/9 proprietary CD58.1 399907 BioLegend
CD8a GCTGCGCTTTCCATT A0080 RPA-T8 proprietary CD8a 399907 BioLegend
CD56 TCCTTTCCTGATAGG A0047 5.1H11 proprietary CD56--NCAM 399907 BioLegend
CD11b GACAAGTGATCTGCA A0161 ICRF44 proprietary CD11b 399907 BioLegend
CD183 (CXCR3) GCGATGGTAGATTAT A0140 G025H7 proprietary CD183--CXCR3 399907 BioLegend
CD30 TCAGGGTGTGCTGTA A0028 BY88 proprietary CD30 399907 BioLegend
CD336 (NKp44) GGGCAATTAGCGAGT A0802 P44-8 proprietary CD336--NKp44 399907 BioLegend
CD69 GTCTCTTGGCTTAAA A0146 FN50 proprietary CD69.1 399907 BioLegend
CD335 (NKp46) ACAATTTGAACAGCG A0101 9E2 proprietary CD335--NKp46 399907 BioLegend
CD49f TTCCGAGGATGATCT A0070 GoH3 proprietary CD49f 399907 BioLegend
CD161 GTACGCAGTCCTTCT A0149 HP-3G10 proprietary CD161 399907 BioLegend
CD45RO CTCCGAATCATGTTG A0087 UCHL1 proprietary CD45RO 399907 BioLegend
CD20 TTCTGGGTCCCTAGA A0100 2H7 proprietary CD20 399907 BioLegend
KLRG1 (MAFA) CTTATTTCCTGCCCT A0153 SA231A2 proprietary KLRG1--MAFA 399907 BioLegend
CD19 CTGGGCAATTACTCG A0050 HIB19 proprietary CD19.1 399907 BioLegend
CD32 GCTTCCGAATTACCG A0142 FUN-2 proprietary CD32 399907 BioLegend
CD276 (B7-H3) GACTGGGAGGGTATT A0010 DCN.70 proprietary CD276--B7-H3 399907 BioLegend
CD279 (PD-1) ACAGCGCCGTATTTA A0088 EH12.2H7 proprietary CD279--PD-1 399907 BioLegend
CD197 (CCR7) AGTTCAGTCAACCGA A0148 G043H7 proprietary CD197--CCR7 399907 BioLegend
CD252 (OX40L) TTTAGTGATCCGACT A0021 11C3.1 proprietary N/A 399907 BioLegend
CD223 (LAG-3) CATTTGTCTGCCGGT A0152 11C3C65 proprietary CD223--LAG-3 399907 BioLegend
HLA-DR AATAGCGAGCAAGTA A0159 L243 proprietary HLA-DR 399907 BioLegend
CD45RA TCAATCCTTCCGCTT A0063 HI100 proprietary CD45RA 399907 BioLegend
CD196 (CCR6) GATCCCTTTGTCACT A0143 G034E3 proprietary CD196--CCR6 399907 BioLegend
CD2 TACGATTTGTCAGGG A0367 TS1/8 proprietary CD2.1 399907 BioLegend
CD326 (Ep-CAM) TTCCGAGCAAGTATC A0123 9C4 proprietary CD326--EpCAM 399907 BioLegend
CD169 (Sialoadhesin, Siglec-1) TACTCAGCGTGTTTG A0206 7-239 proprietary CD169--Sialoadhesin--Siglec-1 399907 BioLegend
CD41 ACGTTGTGGCCTTGT A0353 HIP8 proprietary CD41 399907 BioLegend
CD49b GCTTTCTTCAGTATG A0371 P1E6-C5 proprietary CD49b 399907 BioLegend
CD303 (BDCA-2) GAGATGTCCGAATTT A0370 201A proprietary CD303--BDCA-2 399907 BioLegend
CD29 GTATTCCCTCAGTCA A0369 TS2/16 proprietary CD29 399907 BioLegend
CD184 (CXCR4) TCAGGTCCTTTCAAC A0366 12G5 proprietary CD184--CXCR4 399907 BioLegend
CD98 GCACCAACAGCCATT A0374 MEM-108 proprietary CD98 399907 BioLegend
CD124 (IL-4R) CCGTCCTGATAGATG A0363 G077F6 proprietary N/A 399907 BioLegend
CD135 (Flt-3/Flk-2) CAGTAGATGGAGCAT A0351 BV10A4H2 proprietary CD135 399907 BioLegend
CD370 (CLEC9A/DNGR1) CTGCATTTCAGTAAG A0207 8F9 proprietary CD370--CLEC9A--DNGR1 399907 BioLegend
CD21 AACCTAGTAGTTCGG A0181 Bu32 proprietary CD21 399907 BioLegend

CD39 TTACCTGGTATCCGT A0176 A1 proprietary CD39 399907 BioLegend
CD141 (Thrombomodulin) GGATAACCGCGCTTT A0163 M80 proprietary CD141--Thrombomodulin 399907 BioLegend
CD64 AAGTATGCCCTACGA A0162 10.1 proprietary CD64 399907 BioLegend
CD152 (CTLA-4) ATGGTTCACGTAATC A0151 BNI3 proprietary CD152 399907 BioLegend
CD61 AGGTTGGAGTAGACT A0372 VI-PL2 proprietary CD61 399907 BioLegend
CD163 GCTTCTCCTTCCTTA A0358 GHI/61 proprietary CD163.1 399907 BioLegend
CD357 (GITR) ACCTTTCGACACTCG A0360 108-17 proprietary CD357--GITR 399907 BioLegend
CD137 (4-1BB) CAGTAAGTTCGGGAC A0355 4B4-1 proprietary CD137 399907 BioLegend
CD81 (TAPA-1) GTATCCTTCCTTGGC A0373 5A6 proprietary CD81--TAPA-1 399907 BioLegend
CD366 (Tim-3) TGTCCTACCCAACTT A0169 F38-2E2 proprietary CD366--Tim-3 399907 BioLegend
CD57 Recombinant AACTCCCTATGGAGG A0168 QA17A04 proprietary CD57 399907 BioLegend
CD278 (ICOS) CGCGCACCCATTAAA A0171 C398.4A proprietary CD278--ICOS 399907 BioLegend
CD95 (Fas) CCAGCTCATTAGAGC A0156 DX2 proprietary CD95--Fas 399907 BioLegend
CD80 ACGAATCAATCTGTG A0005 2D10 proprietary CD80.1 399907 BioLegend
CD138 (Syndecan-1) ACTCTTTCGTTTACG A0055 MI15 proprietary CD138--Syndecan-1 399907 BioLegend
CD70 CGCGAACATAAGAAG A0027 113-16 proprietary CD70.1 399907 BioLegend
CD269 (BCMA) CAGATGATCCACCAT A0056 19F2 proprietary CD269--BCMA 399907 BioLegend
IgM TAGCGAGCCCGTATA A0136 MHM-88 proprietary IgM 399907 BioLegend
CD59 AATTAGCCGTCGAGA A0361 p282 (H19) proprietary CD59.1 399907 BioLegend
CD194 (CCR4) AGCTTACCTGCACGA A0071 L291H4 proprietary CD194--CCR4 399907 BioLegend
CD275 (B7-H2, ICOSL) GTGCATTCAACAGTA A0009 2D3 proprietary CD275--B7-H2--ICOSL 399907 BioLegend
CD314 (NKG2D) CGTGTTTGTTCCTCA A0165 1D11 proprietary CD314--NKG2D 399907 BioLegend
CD86 GTCTTTGTCAGTGCA A0006 IT2.2 proprietary CD86.1 399907 BioLegend
Galectin-9 ACTCACTGGAGTCTC A0016 9M1-3 proprietary N/A 399907 BioLegend
CD195 (CCR5) CCAAAGTAAGAGCCA A0141 J418F1 proprietary CD195--CCR5 399907 BioLegend
CD1c GAGCTACTTCACTCG A0160 L161 proprietary CD1c 399907 BioLegend
CD28 TGAGAACGACCCTAA A0386 CD28.2 proprietary CD28.1 399907 BioLegend
TSLPR (TSLP-R) CAGTCCTCTCTGTCA A0387 1D3 proprietary TSLPR 399907 BioLegend
CD38 TGTACCCGCTTGTGA A0389 HIT2 proprietary CD38.1 399907 BioLegend
CD155 (PVR) ATCACATCGTTGCCA A0023 SKII.4 proprietary CD155--PVR 399907 BioLegend
CD270 (HVEM, TR2) TGATAGAAACAGACC A0020 122 proprietary CD270--HVEM--TR2 399907 BioLegend
CD178 (Fas-L) CCGGTCCTCTGTATT A0177 NOK-1 proprietary CD178--FasL 399907 BioLegend
CD127 (IL-7R) GTGTGTTGTCCTATG A0390 A019D5 proprietary CD127--IL-7Ra 399907 BioLegend
CD7 TGGATTCCCGGACTT A0066 CD7-6B7 proprietary CD7.1 399907 BioLegend
CD117 (c-kit) AGACTAATAGCTGAC A0061 104D2 proprietary CD117--c-kit 399907 BioLegend
CD10 CAGCCATTCATTAGG A0062 HI10a proprietary CD10 399907 BioLegend
CD40 CTCAGATGGAGTATG A0031 5C3 proprietary CD40.1 399907 BioLegend
CD48 CTACGACGTAGAAGA A0029 BJ40 proprietary CD48.1 399907 BioLegend
CD154 GCTAGATAGATGCAA A0032 24-31 proprietary CD154 399907 BioLegend
CD47 GCATTCTGTCACCTA A0026 CC2C6 proprietary CD47.1 399907 BioLegend
CD112 (Nectin-2) AACCTTCCGTCTAAG A0024 TX31 proprietary CD112--Nectin-2 399907 BioLegend
CD52 CTTTGTACGAGCAAA A0033 HI186 proprietary CD52.1 399907 BioLegend
CD71 CCGTGTTCCTCATTA A0394 CY1G4 proprietary CD71 399907 BioLegend
CD22 GGGTTGTTGTCTTTG A0393 S-HCL-1 proprietary CD22.1 399907 BioLegend
CD15 (SSEA-1) TCACCAGTACCTAGT A0392 W6D3 proprietary CD15--BEA-1 399907 BioLegend
XCR1 AAGACGCATGTCAAC A0208 S15046E proprietary XCR1.1 399907 BioLegend
CD26 GGTGGCTAGATAATG A0396 BA5b proprietary CD26 399907 BioLegend
B7-H4 TGTATGTCTGCCTTG A0395 MIH43 proprietary B7-H4 399907 BioLegend
CD31 ACCTTTATGCCACGG A0124 WM59 proprietary CD31 399907 BioLegend
CD14 TCTCAGACCTCCGTA A0081 M5E2 proprietary CD14.2 399907 BioLegend
TIGIT (VSTM3) TTGCTTACCGCCAGA A0089 A15153G proprietary TIGIT--VSTM3 399907 BioLegend
CD294 (CRTH2) TGTTTACGAGAGCCC A0102 BM16 proprietary CD294--CRTH2 399907 BioLegend
CD45 TCCCTTGCGATTTAC A0048 2D1 proprietary CD45 399907 BioLegend
CD34 GCAGAAATCTCCCTT A0054 581 proprietary CD34.1 399907 BioLegend
CD244 (2B4) TCGCTTGGATGGTAG A0189 C1.7 proprietary CD244--2B4 399907 BioLegend
CD185 (CXCR5) AATTCAACCGTCGCC A0144 J252D4 proprietary CD185--CXCR5 399907 BioLegend
CD18 TATTGGGACACTTCT A0385 TS1/18 proprietary CD18 399907 BioLegend
CD119 (IFN-? R ? chain) TGTGTATTCCCTTGT A0219 GIR-208 proprietary CD119--IFN-G-R-A-chain 399907 BioLegend
CD134 (OX40) AACCCACCGTTGTTA A0158 Ber-ACT35 (ACT35) proprietary CD134--OX40 399907 BioLegend
CD24 AGATTCCTTCGTGTT A0180 ML5 proprietary CD24.1 399907 BioLegend
TCR / CTTCCGATTCATTCA A0139 B1 proprietary TCR-G--TCR-D 399907 BioLegend
CD115 (CSF-1R) AATCACGGTCCTTGT A0398 9-4D2-1E4 proprietary CD115--CSF-1R 399907 BioLegend
CD1d TCGAGTCGCTTATCA A0164 51.1 proprietary CD1d 399907 BioLegend
CD192 (CCR2) GAGTTCCCTTACCTG A0242 K036C2 proprietary CD192--CCR2 399907 BioLegend
CD105 ATCGTCGAGAGCTAG A0068 43A3 proprietary CD105 399907 BioLegend
CD144 (VE-Cadherin) TCCACTCATTCTGTA A0400 BV9 proprietary CD144--VE-cadherin 399907 BioLegend
CD193 (CCR3) ACCAATCCTTTCGTC A0397 5E8 proprietary CD193--CCR3 399907 BioLegend
CD55 GCTCATTACCCATTA A0383 JS11 proprietary CD55.1 399907 BioLegend

CD79b (Igβ) ATTCTTCAACCGAAG A0187 CB3-1 proprietary CD79b--Ig-B 399907 BioLegend
CD123 CTTCACTCTGTCAGG A0064 6H6 proprietary CD123 399907 BioLegend
CD11c TACGCCTATAACTTG A0053 S-HCL-3 proprietary CD11c 399907 BioLegend
CD54 CTGATAGACTTGAGT A0217 HA58 proprietary CD54 399907 BioLegend
CD268 (BAFF-R) CGAAGTCGATCCGTA A0215 11C1 proprietary CD268--BAFF-R 399907 BioLegend
TIM-4 CGTCATATAGTATGG A0428 9F4 proprietary Tim-4 399907 BioLegend
CD1a GATCGTGTTGTGTTA A0402 HI149 proprietary CD1a 399907 BioLegend
CD301 (CLEC10A) ACCTAGAAATCAGCA A0401 H037G3 proprietary CD301--CLEC10A 399907 BioLegend
CD63 GAGATGTCTGCAACT A0404 H5C6 proprietary CD63.1 399907 BioLegend
CD44 AATCCTTCCGAATGT A0125 BJ18 proprietary CD44.1 399907 BioLegend
IgD CAGTCTCCGTAGAGT A0384 IA6-2 proprietary IgD 399907 BioLegend
CD72 CAGTCGTGGTAGATA A0419 3F3 proprietary CD72.1 399907 BioLegend
CD93 GCGCTACTTCCTTGA A0446 VIMD2 proprietary CD93.1 399907 BioLegend
CD85g (ILT7) TGTCAGTTCCTATGA A0409 17G10.2 proprietary CD85g--ILT7 399907 BioLegend
CD172a (SIRP) CGTGTTTAACTTGAG A0408 15-414 proprietary CD172a--SIRPa 399907 BioLegend
CD284 (TLR4) GCTTAGCTGTATCCG A0405 HTA125 proprietary CD284--TLR4 399907 BioLegend
CD304 (Neuropilin-1) GGACTAAGTTTCGTT A0406 12C2 proprietary CD304--Neuropilin-1 399907 BioLegend
CD36 TTCTTTGCCTTGCCA A0407 5-271 proprietary CD36.1 399907 BioLegend
CD102 (ICAM-2) TGACCTTCCTCTCCT A0244 CBR-IC2/2 proprietary CD102 399907 BioLegend
TCR/β CGTAACGTAGAGCGA A0224 IP26 proprietary TCR-A--TCR-B 399907 BioLegend
CD122 (IL-2R?) TCATTTCCTCCGATT A0246 TU27 proprietary N/A 399907 BioLegend
CD83 CCACTCATTTCCGGT A0359 HB15e proprietary CD83.1 399907 BioLegend
CD267 (TACI) AGTGATGGAGCGAAC A0247 1A1 proprietary CD267--TACI 399907 BioLegend
anti-human IgA AAGATGTCCGAGCAA A0186 #N/A proprietary N/A 399907 BioLegend
CD107a (LAMP-1) CAGCCCACTGCAATA A0155 H4A3 proprietary CD107a--LAMP-1 399907 BioLegend
CD146 CCTTGGATAACATCA A0134 P1H12 proprietary CD146 399907 BioLegend
CD140b (PDGFR?) CAATGGTTCACTGCC A0129 18A2 proprietary CD140b 399907 BioLegend
CD49a ACTGATGGACTCAGA A0575 TS2/7 proprietary CD49a 399907 BioLegend
AACTTCTGTGGTAGC A0584 6B11 proprietary TCR-V-A-24-J-A-18--iNKT cell 399907 BioLegend
CD9 GAGTCACCAATCTGC A0579 HI9a proprietary CD9.1 399907 BioLegend
TCR V7.2 TACGAGCAGTATTCA A0581 3C10 proprietary TCR-V-A-7.2 399907 BioLegend
TCR V 9 AAGTGATGGTATCTG A0583 B3 proprietary TCR-V-G-9 399907 BioLegend
CD49d CCATTCAACTTCCGG A0576 9F10 proprietary CD49d 399907 BioLegend
C5L2 ACAATTTGTCTGCGA A0572 1D9-M12 proprietary C5L2 399907 BioLegend
CD338 (ABCG2) TAAGACTTGGCCGTC A0569 5D3 proprietary CD338--ABCG2 399907 BioLegend
CD73 (Ecto-5'-nucleotidase) CAGTTCCTCAGTTCG A0577 AD2 proprietary CD73--Ecto-5-nucleotidase 399907 BioLegend
anti-human CD79a (Ig?) CTTATCACCCGCTTT A0578 #N/A proprietary N/A 399907 BioLegend
CD158b (KIR2DL2/L3, NKAT2) GACCCGTAGTTTGAT A0592 DX27 proprietary CD158b--KIR2DL2--KIR2DL3--NKAT2 399907 BioLegend
CD226 (DNAM-1) AGACCAACTCATTCA A0805 TX25 proprietary CD226--DNAM-1.1 399907 BioLegend
CD186 (CXCR6) GACAGTCGATGCAAC A0804 K041E5 proprietary CD186--CXCR6 399907 BioLegend
CD158f (KIR2DL5) AAAGTGATGCCACTG A0600 UP-R1 proprietary CD158f--KIR2DL5 399907 BioLegend
CD158e1 (KIR3DL1, NKB1) GGACGCTTTCCTTGA A0599 DX9 proprietary CD158e1--KIR3DL1--NKB1 399907 BioLegend
CD140a (PDGFR?) ATGCGCCGAGAATTA A0128 16A1 proprietary CD140a 399907 BioLegend
CD354 (TREM-1) TAGCCGTTTCCTTTG A0586 TREM-26 proprietary N/A 399907 BioLegend
TCR V 2 TCAGTCAGATGGTAT A0582 B6 proprietary TCR-V-D-2 399907 BioLegend
CD202b (Tie2/Tek) CGATCCCTTACCTAT A0588 33.1 (Ab33) proprietary CD202b--Tie2--Tek 399907 BioLegend
CD96 (TACTILE) TGGCCTATAAATGGT A0175 NK92.39 proprietary CD96--TACTILE 399907 BioLegend
CD158 (KIR2DL1/S1/S3/S5) TATCAACCAACGCTT A0420 HP-MA4 proprietary CD158--KIR2DL1--KIR2DS1--KIR2DS3--KIR2DS5 399907 BioLegend
CD337 (NKp30) AAAGTCACTCTGCCG A0801 P30-15 proprietary CD337--NKp30 399907 BioLegend
CD253 (Trail) GCCATTCCTGCCTAA A0803 RIK-2 proprietary CD253--TRAIL 399907 BioLegend
CD319 (CRACC) AGTATGCCATGTCTT A0830 162.1 proprietary CD319--CRACC 399907 BioLegend
CD305 (LAIR1) ATTTCCATTCCCTGT A0590 NKTA255 proprietary CD305--LAIR1 399907 BioLegend
CD325 (N-Cadherin) CCTTCCCTTTCCTCT A0433 8C11 proprietary CD325--N-Cadherin 399907 BioLegend
mast cell tryptase ACTGATAGACCCGCT A0580 AA1 proprietary N/A 399907 BioLegend
CLEC12A CATTAGAGTCTGCCA A0853 50C1 proprietary CD371--CLEC12A 399907 BioLegend
CD90 (Thy1) GCATTGTACGATTCA A0060 5E10 proprietary CD90--Thy1 399907 BioLegend
CD273 (B7-DC, PD-L2) TCAACGCTTGGCTAG A0008 24F.10C12 proprietary CD273--B7-DC--PD-L2 399907 BioLegend
CD272 (BTLA) GTTATTGGACTAAGG A0170 MIH26 proprietary CD272--BTLA 399907 BioLegend
CD5 CATTAACGGGATGCC A0138 UCHT2 proprietary CD5.1 399907 BioLegend
CD23 TCTGTATAACCGTCT A0897 EBVCS-5 proprietary CD23 399907 BioLegend
CD85j (ILT2) CCTTGTGAGGCTATG A0896 GHI/75 proprietary CD85j--ILT2 399907 BioLegend
CD94 CTTTCCGGTCCTACA A0867 DX22 proprietary CD94 399907 BioLegend
CD27 GCACTCCTGCATGTA A0154 O323 proprietary CD27.1 399907 BioLegend
CD328 (Siglec-7) CTTAGCATTTCACTG A0902 6-434 proprietary CD328--Siglec-7 399907 BioLegend
CD82 TCCCACTTCCGCTTT A0920 ASL-24 proprietary N/A 399907 BioLegend
CD16 AAGTTCACTCTTTGC A0083 3G8 proprietary CD16 399907 BioLegend
MERTK TCCTGCATGTACCCA A0423 590H11G1E3 proprietary MERTK.1 399907 BioLegend
IgG Fc CTGGAGCGATTAGAA A0375 M1310G05 proprietary IgG-Fc 399907 BioLegend

FcεRI CTCGTTTCCGTATCG A0352 AER-37 (CRA-1) proprietary FceRIa 399907 BioLegend
CD274 (B7-H1, PD-L1) GTTGTCCGACAATAC A0007 29E.2A3 proprietary CD274--B7-H1--PD-L1 399907 BioLegend
CD254 (TRANCE, RANKL) TCCGTGTTAGTTTGT A0356 MIH24 proprietary CD254--TRANCE--RANKL 399907 BioLegend
CX3CR1 AGTATCGTCTCTGGG A0179 K0124E1 proprietary CX3CR1.1 399907 BioLegend
CD137L (4-1BB Ligand) ATTCGCCTTACGCAA A0022 5F4 proprietary CD137L--4-1BB-Ligand 399907 BioLegend
Siglec-8 CTTCTCCTCAGCAAT A0199 7C9 proprietary N/A 399907 BioLegend

Validation

CITE-seq antibodies from Biolegend have been validated by the manufacturer. CODEX antibodies were validated by Akoya Biosciences, and non-specific antibodies in the panel were excluded from phenotype analysis. MIBI and CyTOF antibodies were validated by their respective manufacturer, and then individually tested and titrated by serial dilution on relevant positive and negative controls by investigators to identify optimal concentrations.

Validation of antibodies for CyTOF:
https://www.biolegend.com/en-us/soluble-mhc/purified-anti-human-cd45-antibody-710
https://www.biolegend.com/ja-jp/products/purified-anti-human-cd326-epcam-antibody-3755
https://www.bdbiosciences.com/en-us/products/reagents/flow-cytometry-reagents/research-reagents/single-color-antibodies-ruo/purified-mouse-anti-human-cd7.555359
https://www.biolegend.com/de-de/sean-tuckers-tests/purified-anti-human-cd15-ssea-1-antibody-3699
https://www.biolegend.com/en-gb/cell-health/purified-anti-human-cd3-antibody-867
https://www.biolegend.com/de-at/sean-tuckers-tests/purified-anti-human-cd19-antibody-721
https://www.biolegend.com/de-at/products/purified-anti-human-cd163-antibody-4789?GroupID=BLG10122
https://www.thermofisher.com/antibody/product/CD4-Antibody-clone-RPA-T4-Monoclonal/14-0049-82
https://www.biolegend.com/nl-be/cell-health/purified-anti-human-cd8a-antibody-839
https://www.thermofisher.com/antibody/product/CD11c-Antibody-clone-BU15-Monoclonal/MA1-82142
https://www.biolegend.com/en-us/cell-separation/purified-anti-human-cd14-antibody-797?GroupID=BLG4805
https://www.biolegend.com/en-ie/productstab/purified-anti-human-cd127-il-7ralpha-antibody-7093?GroupID=BLG9274
https://www.thermofisher.com/antibody/product/CD123-Antibody-clone-6H6-Monoclonal/14-1239-82
https://www.biolegend.com/en-ie/lyophilized-control-cells/purified-anti-human-cd45ra-antibody-689
https://www.biolegend.com/de-de/products/purified-anti-human-cd366-tim-3-antibody-6119?GroupID=BLG9937
https://www.biolegend.com/en-gb/soluble-mhc/ultra-leaf-purified-anti-human-tigit-vstm3-antibody-14287
https://www.biolegend.com/en-us/cell-health/purified-anti-human-cd274-b7-h1-pd-l1-antibody-4373
https://www.biolegend.com/nl-nl/products/apc-anti-human-cd49d-antibody-582?GroupID=BLG1167
https://www.biolegend.com/en-gb/productstab/purified-anti-human-cd27-antibody-812?GroupID=BLG10174
https://www.thermofisher.com/antibody/product/CD137-Ligand-4-1BB-Ligand-Antibody-clone-5G11-Monoclonal/14-9056-82
https://www.thermofisher.com/antibody/product/T-bet-Antibody-clone-eBio4B10-4B10-Monoclonal/14-5825-82
https://www.thermofisher.com/antibody/product/CD152-CTLA-4-Antibody-clone-14D3-Monoclonal/14-1529-82
https://www.thermofisher.com/antibody/product/FOXP3-Antibody-clone-PCH101-Monoclonal/14-4776-82
https://www.biolegend.com/de-de/products/purified-anti-human-cd31-antibody-883?Clone=WM59
https://www.biolegend.com/nl-nl/cell-health/purified-anti-human-mouse-integrin-beta7-antibody-2951
https://www.biolegend.com/nl-nl/antibodies-and-more/purified-anti-human-cd141-thrombomodulin-antibody-6106
https://www.biolegend.com/en-gb/clone-search/purified-anti-human-cd197-ccr7-antibody-7471?GroupID=BLG9610
https://www.biolegend.com/en-gb/search-results/purified-anti-human-ki-67-antibody-6967
https://www.biolegend.com/en-gb/cell-health/purified-anti-human-cd25-antibody-8323?GroupID=BLG10308
https://www.biolegend.com/en-gb/cell-health/purified-anti-human-cd38-antibody-748
https://www.biolegend.com/nl-nl/products/purified-anti-human-cd1c-antibody-4836?Clone=L161
https://www.biolegend.com/en-gb/cell-health/purified-anti-human-mouse-rat-cd278-icos-antibody-2477?GroupID=BLG3831
https://www.biolegend.com/en-us/soluble-mhc/purified-anti-human-hla-dr-antibody-792?GroupID=BLG11943
https://www.biolegend.com/fr-lu/cell-health/purified-anti-human-cd279-pd-1-antibody-4410
https://www.bdbiosciences.com/en-us/products/reagents/flow-cytometry-reagents/research-reagents/single-color-antibodies-ruo/purified-mouse-anti-human-cd56.559043
https://www.biolegend.com/fr-lu/products/purified-anti-human-cd16-antibody-571?GroupID=BLG8436

Validation antibodies for MIBI:
https://www.abcam.com/products/primary-antibodies/ds-dna-antibody-35i9-dna-bsa-and-azide-free-ab27156.html
https://www.abcam.com/products/primary-antibodies/e-cadherin-antibody-36e-cadherin-bsa-and-azide-free-ab287971.html
https://www.ionpath.com/histone-h3-antibody/
https://www.bdbiosciences.com/en-us/products/reagents/flow-cytometry-reagents/research-reagents/single-color-antibodies-ruo/alexa-fluor-488-mouse-anti-human-foxp3.561181
https://www.thermofisher.com/antibody/product/Alexa-Fluor-488-Antibody-Polyclonal/A-11094
https://www.abcam.com/products/primary-antibodies/cd163-antibody-epr14643-36-low-endotoxin-azide-free-ab215976.html
https://www.ionpath.com/cd4-antibody/
https://www.ionpath.com/cd11c-antibody/
https://www.cellsignal.com/products/primary-antibodies/cd14-d7a2t-rabbit-mab-bsa-and-azide-free/43878
https://www.cellsignal.com/products/primary-antibodies/cd16-d1n9l-rabbit-mab-bsa-and-azide-free/72204
https://www.novusbio.com/products/lag-3-antibody-17b4_nbp1-97657
https://www.ionpath.com/pd-1-antibody/
https://www.ionpath.com/pd-l1-antibody/
https://www.ionpath.com/granzyme-b-d6e9w-antibody/
https://www.ionpath.com/cd56-antibody-151eu/

https://www.ionpath.com/cd31-antibody-152sm/
https://www.cellsignal.com/products/primary-antibodies/ki-67-d2h10-rabbit-mab-bsa-and-azide-free/44092
https://www.ionpath.com/cd117-antibody-155gd/
https://www.ionpath.com/cd68-antibody/
https://www.abcam.com/en-za/products/primary-antibodies/cd103-antibody-epr41662-bsa-and-azide-free-ab271889
https://www.ionpath.com/cd8-antibody-158gd/
www.ionpath.com/wp-content/uploads/2021/03/TDS-715901_B-CD3e_D7A6E_159Tb_Human_FFPE.pdf
https://www.ionpath.com/cd45ro-antibody-161dv/
https://www.ionpath.com/wp-content/uploads/2021/03/TDS-716201_B-TIM-3_EPR22241_162Dy_Human_FFPE.pdf
https://www.ionpath.com/vimentin-antibody/
https://www.abcam.com/products/primary-antibodies/cd27-antibody-epr8569-bsa-and-azide-free-ab256583.html
https://www.ionpath.com/keratin-antibody-165ho/
https://www.abcam.com/en-in/products/primary-antibodies/alpha-smooth-muscle-actin-antibody-sp171-bsa-and-azide-free-ab242395
https://www.ionpath.com/cd20-antibody-167er/
https://www.abcam.com/products/primary-antibodies/cd11b-antibody-ep1345y-bsa-and-azide-free-ab187537.html
https://www.cellsignal.com/products/primary-antibodies/cd141-thrombomodulin-e7y9p-xp-rabbit-mab-bsa-and-azide-free/34149
https://www.ionpath.com/cd21-antibody-170er/
https://www.ionpath.com/ido1-antibody-171yb/
https://www.ionpath.com/hla-dr-antibody/
https://www.cellsignal.com/products/primary-antibodies/epcam-d9s3p-rabbit-mab-bsa-and-azide-free/55725
https://www.ionpath.com/cd45-antibody-175lu/
https://www.ionpath.com/wp-content/uploads/2021/03/TDS-717602_B-HLA-class-1ABC_EMR8-5_176Yb_Human_FFPE.pdf
https://www.abcam.com/products/primary-antibodies/syndecan-1-antibody-epr6454-bsa-and-azide-free-ab226108.html
https://www.cellsignal.com/products/primary-antibodies/t-bet-tbx21-d6n8b-xp-rabbit-mab-bsa-and-azide-free/27112

Validation CODEX:
https://www.abcam.com/products/primary-antibodies/hla-a-antibody-ep1395y-bsa-and-azide-free-ab216653.html
https://www.thermofisher.com/antibody/product/CD34-Antibody-clone-QBEND-10-Monoclonal/MA1-10205
https://www.abcam.com/products/primary-antibodies/cd4-antibody-epr6855-ab133616.html
https://www.thermofisher.com/antibody/product/CD20-Antibody-clone-L26-Monoclonal/14-0202-82
https://www.abcam.com/products/primary-antibodies/cd14-antibody-epr3653-ab133335.html
https://www.thermofisher.com/antibody/product/CD68-Antibody-clone-KP1-Monoclonal/MA5-13324
https://www.biolegend.com/fr-fr/search-results/purified-anti-vimentin-antibody-12022?GroupID=GROUP26
https://www.biolegend.com/nl-nl/products/biotin-anti-human-cd8a-antibody-16285
https://www.thermofisher.com/antibody/product/CD11c-Antibody-clone-118-A5-Monoclonal/14-9761-82
https://www.abcam.com/products/primary-antibodies/cd31-antibody-ep3095-ab134168.html
https://www.thermofisher.com/antibody/product/E-cadherin-Antibody-clone-4A2C7-Monoclonal/33-4000
https://www.cellsignal.com/products/primary-antibodies/cd45-intracellular-domain-d9m8i-xp-rabbit-mab/13917
https://www.abcam.com/products/primary-antibodies/alpha-smooth-muscle-actin-antibody-1a4-ab7817.html
https://www.biolegend.com/de-at/products/purified-anti-human-cd45ro-antibody-860?Clone=UCHL1
https://www.abcam.com/products/primary-antibodies/cd3-epsilon-antibody-ep449e-ab52959.html
https://www.biolegend.com/fr-ch/products/purified-anti-pan-cytokeratin-antibody-13345?GroupID=GROUP26
https://www.cellsignal.com/products/primary-antibodies/cd44-156-3c11-mouse-mab/3570
https://www.abcam.com/products/primary-antibodies/hla-dr-antibody-epr3692-ab92511.html
https://www.cellsignal.com/products/primary-antibodies/granzyme-b-d6e9w-rabbit-mab/46890
https://www.abcam.com/products/primary-antibodies/collagen-iv-antibody-epr20966-ab214417.html
https://www.biolegend.com/en-gb/productstab/purified-anti-human-podoplanin-antibody-5797?GroupID=GROUP28
https://www.bdbiosciences.com/en-us/products/reagents/flow-cytometry-reagents/research-reagents/single-color-antibodies-ruo/purified-mouse-anti-human-foxp3.560044
https://www.bdbiosciences.com/en-us/products/reagents/microscopy-imaging-reagents/immunofluorescence-reagents/purified-mouse-anti-ki-67.556003
https://www.cellsignal.com/products/primary-antibodies/cd163-d6u1j-rabbit-mab/93498
https://www.revmab.com/index.php/product/anti-cd-19-rabbit-monoclonal-antibody-clone-rm332/
https://www.abcam.com/products/primary-antibodies/cd11b-antibody-ep1345y-c-terminal-ab52478.html
https://www.abcam.com/products/primary-antibodies/cd21-antibody-ep3093-ab75985.html
https://www.biolegend.com/en-gb/products/apc-anti-human-cd57-antibody-9023?GroupID=BLG8483#:~:text=CD57%2C%20also%20known%20as%20HNK,lipids%2C%20and%20chondroitin%20sulfate%20proteoglycans.

Validation for CITE-seq:
https://www.biolegend.com/en-ie/products/totalseq-a0145-anti-human-cd103-integrin-%CE%B1e-antibody-16194?GroupID=BLG15664
https://www.biolegend.com/fr-ch/products/totalseq-a0167-anti-human-cd35-antibody-16337
https://www.biolegend.com/en-gb/products/totalseq-a0085-anti-human-cd25-antibody-15770
https://www.biolegend.com/en-gb/products/totalseq-a0147-anti-human-cd62l-antibody-16334?GroupID=BLG10034
https://www.biolegend.com/de-at/products/totalseq-a0034-anti-human-cd3-antibody-15707
https://www.biolegend.com/nl-be/antibodies-and-more/totalseq-a0052-anti-human-cd33-antibody-15933?GroupID=GROUP28
https://www.biolegend.com/nl-be/products/totalseq-a0072-anti-human-cd4-antibody-15762

https://www.biolegend.com/en-ie/products/totalseq-a0174-anti-human-cd58-lfa-3-antibody-16164?GroupID=BLG4872
https://www.biolegend.com/fr-fr/cell-separation/totalseq-a0080-anti-human-cd8a-antibody-15763
https://www.biolegend.com/nl-be/explore-new-products/totalseq-a0047-anti-human-cd56-ncam-antibody-16180
https://www.biolegend.com/ja-jp/search-results/totalseq-a0161-anti-human-cd11b-antibody-15927?Clone=ICRF44
https://www.biolegend.com/en-gb/explore-new-products/totalseq-a0140-anti-human-cd183-cxcr3-antibody-16163
https://www.biolegend.com/en-gb/products/totalseq-a0028-anti-human-cd30-antibody-16188?GroupID=BLG5847
https://www.biolegend.com/de-at/products/totalseq-a0802-anti-human-cd336-nkp44-antibody-18590
https://www.biolegend.com/en-ie/products/totalseq-a0146-anti-human-cd69-antibody-16200
https://www.biolegend.com/en-us/punchout/punchout-products/product-detail/totalseq-a0101-anti-human-cd335-nkp46-antibody-17433
https://www.biolegend.com/ja-jp/products/totalseq-a0070-anti-human-mouse-cd49f-antibody-15953
https://www.biolegend.com/fr-lu/products/totalseq-a0149-anti-human-cd161-antibody-16156
https://www.biolegend.com/fr-lu/products/totalseq-a0087-anti-human-cd45ro-antibody-15771
https://www.biolegend.com/fr-ch/products/totalseq-b0100-anti-human-cd20-antibody-16833
https://www.biolegend.com/nl-nl/products/totalseq-a0153-anti-human-klrg1-afa-antibody-16530
https://www.biolegend.com/en-gb/products/totalseq-a0050-anti-human-cd19-antibody-15777
https://punchout.biolegend.com/en-gb/products/totalseq-d0142-anti-human-cd32-antibody-22755
https://www.biolegend.com/en-ie/products/totalseq-a0010-anti-human-cd276-b7-h3-antibody-15940
https://www.biolegend.com/en-ie/products/totalseq-a0088-anti-human-cd279-pd-1-antibody-15772?GroupID=BLG5466
https://www.biolegend.com/fr-ch/products/totalseq-a0148-anti-human-cd197-ccr7-antibody-16352?GroupID=BLG9613
https://www.biolegend.com/en-gb/products/totalseq-a0152-anti-human-cd223-lag-3-antibody-16157?GroupID=BLG14890
https://www.biolegend.com/fr-ch/products/totalseq-a0159-anti-human-hla-dr-antibody-16189
https://www.biolegend.com/en-gb/cell-separation/totalseq-a0063-anti-human-cd45ra-antibody-15775?GroupID=GROUP658
https://www.biolegend.com/nl-be/products/totalseq-a0143-anti-human-cd196-ccr6-antibody-16436
https://www.biolegend.com/en-us/clone-search/totalseq-a0367-anti-human-cd2-antibody-16714
https://www.biolegend.com/nl-be/explore-new-products/totalseq-a0123-anti-human-cd326-ep-cam-antibody-16453?GroupID=BLG5134
https://www.biolegend.com/ja-jp/products/totalseq-a0206-anti-human-cd169-sialoadhesin-siglec-1-antibody-16160
https://www.biolegend.com/fr-ch/cell-separation/totalseq-a0353-anti-human-cd41-antibody-16620?GroupID=BLG5923
https://www.biolegend.com/fr-ch/products/totalseq-a0371-anti-human-cd49b-antibody-16527?GroupID=BLG4932
https://www.biolegend.com/ja-jp/products/totalseq-a0370-anti-human-cd303-bdca-2-antibody-17542
https://www.biolegend.com/nl-be/products/totalseq-a0369-anti-human-cd29-antibody-16664?Clone=TS2/16
https://www.biolegend.com/en-ie/antibodies-and-more/totalseq-a0366-anti-human-cd184-cxcr4-antibody-17277
https://www.biolegend.com/nl-be/products/totalseq-a0374-anti-human-cd98-antibody-16455
https://www.biolegend.com/ja-jp/products/totalseq-a0363-anti-human-cd124-il-4ralpha-antibody-23985
https://www.biolegend.com/de-de/products/totalseq-a0207-anti-human-cd370-clec9a-dngr1-antibody-18106
https://www.biolegend.com/en-gb/products/totalseq-a0181-anti-human-cd21-antibody-16203
https://www.biolegend.com/de-at/products/totalseq-a0176-anti-human-cd39-antibody-16204?GroupID=BLG5462
https://www.biolegend.com/de-at/products/totalseq-a0163-anti-human-cd141-thrombomodulin-antibody-16639?GroupID=BLG10120
https://www.biolegend.com/nl-be/products/totalseq-a0162-anti-human-cd64-antibody-16355?GroupID=GROUP28
https://www.biolegend.com/nl-nl/clone-search/totalseq-a0151-anti-human-cd152-ctla-4-antibody-15707?GroupID=BLG14940
https://www.biolegend.com/nl-be/cell-separation/totalseq-a0372-anti-human-cd61-antibody-16622
https://www.biolegend.com/en-gb/products/totalseq-a0358-anti-human-cd163-antibody-16452?GroupID=BLG9966
https://www.biolegend.com/de-de/products/totalseq-a0360-anti-human-cd357-gitr-antibody-17349
https://www.biolegend.com/fr-fr/products/totalseq-trade-a0355-anti-human-cd137-4-1bb-antibody-16737?GroupID=BLG2203
https://www.biolegend.com/fr-ch/products/totalseq-a0373-anti-human-cd81-tapa-1-antibody-17207
https://www.biolegend.com/en-gb/products/totalseq-a0169-anti-human-cd366-tim-3-antibody-17350?Clone=F38-2E2
https://www.biolegend.com/de-de/products/totalseq-a0168-anti-human-cd57-recombinant-antibody-17680?GroupID=GROUP530
https://www.biolegend.com/ja-jp/products/totalseq-a0171-anti-human-mouse-rat-cd278-icos-antibody-17152?GroupID=BLG3827
https://www.biolegend.com/de-at/products/totalseq-a0156-anti-human-cd95-fas-antibody-16363
https://www.biolegend.com/fr-fr/products/totalseq-a0005-anti-human-cd80-antibody-16182?GroupID=BLG8047
https://www.biolegend.com/nl-be/products/totalseq-a0055-anti-human-cd138-syndecan-1-antibody-16181?GroupID=BLG15664
https://www.biolegend.com/en-gb/search-results/totalseq-a0027-anti-human-cd70-antibody-16184?Clone=113-16
https://www.biolegend.com/fr-ch/products/totalseq-a0056-anti-human-cd269-bcma-antibody-16186
https://www.biolegend.com/fr-ch/products/totalseq-a0136-anti-human-igm-antibody-16155?GroupID=BLG4120
https://www.biolegend.com/nl-nl/products/totalseq-a0361-anti-human-cd59-antibody-16396?GroupID=BLG5933
https://www.biolegend.com/fr-lu/products/totalseq-a0071-anti-human-cd194-ccr4-antibody-16170?Clone=L291H4
https://www.biolegend.com/en-gb/products/purified-anti-human-cd275-b7-h2-icosl-antibody-1516?GroupID=BLG2314
https://www.biolegend.com/nl-nl/products/totalseq-a0165-anti-human-cd314-nkg2d-antibody-15958?GroupID=BLG8540
https://www.biolegend.com/nl-be/products/totalseq-a0006-anti-human-cd86-antibody-15937
https://www.biolegend.com/en-gb/products/totalseq-a0141-anti-human-cd195-ccr5-antibody-16161
https://www.biolegend.com/en-gb/search-results/totalseq-a0160-anti-human-cd1c-antibody-15938?GroupID=BLG10089
https://www.biolegend.com/en-gb/search-results/totalseq-a0386-anti-human-cd28-antibody-16787
https://www.biolegend.com/nl-nl/products/totalseq-a0387-anti-human-tslpr-antibody-17066
https://www.biolegend.com/fr-ch/products/totalseq-a0389-anti-human-cd38-antibody-16899
https://www.biolegend.com/de-at/products/totalseq-a0023-anti-human-cd155-pvr-antibody-16350
https://www.biolegend.com/de-at/products/totalseq-a0020-anti-human-cd270-hvem-tr2-antibody-15956?Clone=122
https://www.biolegend.com/nl-nl/products/totalseq-a0177-anti-human-cd178-fas-l-antibody-16360?GroupID=BLG10280

https://www.biolegend.com/de-de/products/totalseq-a0390-anti-human-cd127-il-7ralpha-antibody-15943?GroupID=GROUP28
https://www.biolegend.com/fr-lu/cell-separation/totalseq-a0066-anti-human-cd7-antibody-15944?GroupID=BLG10166
https://www.biolegend.com/en-ie/products/totalseq-a0061-anti-human-cd117-c-kit-antibody-15951?GroupID=BLG8203
https://www.biolegend.com/de-at/explore-new-products/totalseq-a0062-anti-human-cd10-antibody-15949
https://www.biolegend.com/fr-fr/products/totalseq-a0031-anti-human-cd40-antibody-16179
https://www.biolegend.com/fr-ch/products/totalseq-a0029-anti-human-cd48-antibody-15942
https://www.biolegend.com/en-us/products/totalseq-a0032-anti-human-cd154-antibody-15946?Clone=24-31
https://www.biolegend.com/en-gb/products/totalseq-a0026-anti-human-cd47-antibody-15957?GroupID=BLG5112
https://www.biolegend.com/nl-be/products/totalseq-a0024-anti-human-cd112-nectin-2-antibody-16190
https://www.biolegend.com/de-at/products/totalseq-a0033-anti-human-cd52-antibody-15955?GroupID=BLG9977
https://www.biolegend.com/de-de/products/totalseq-a0394-anti-human-cd71-antibody-17086?Clone=CY1G4
https://www.biolegend.com/de-at/products/totalseq-a0393-anti-human-cd22-antibody-15936
https://www.biolegend.com/en-gb/clone-search/totalseq-a0392-anti-human-cd15-ssea-1-antibody-15959?GroupID=BLG4807
https://www.biolegend.com/fr-ch/products/totalseq-a0208-anti-human-xcr1-antibody-16747
https://www.fishersci.com/shop/products/totalseq-tm-a0396-anti-hu-cd2/502072899
https://www.biolegend.com/fr-lu/products/totalseq-a0395-anti-human-b7-h4-antibody-15939
https://www.biolegend.com/nl-nl/products/totalseq-a0124-anti-human-cd31-antibody-16332?Clone=WM59
https://www.biolegend.com/fr-fr/products/totalseq-a0081-anti-human-cd14-antibody-15764
https://www.biolegend.com/nl-be/products/totalseq-a0089-anti-human-tigit-antibody-15773
https://www.biolegend.com/fr-fr/search-results/totalseq-a0102-anti-human-cd294-crth2-antibody-15950
https://www.biolegend.com/fr-ch/products/totalseq-a0048-anti-human-cd45-antibody-15929
https://www.biolegend.com/fr-fr/antibodies-and-more/totalseq-a0054-anti-human-cd34-antibody-16197
https://www.biolegend.com/de-de/products/totalseq-a0189-anti-human-cd244-2b4-antibody-16196?GroupID=BLG8490
https://www.biolegend.com/en-us/punchout/punchout-products/product-detail/totalseq-a0144-anti-human-cd185-cxcr5-antibody-16330
https://www.biolegend.com/en-ie/products/totalseq-a0385-anti-human-cd18-antibody-16434?GroupID=BLG4653

https://www.biolegend.com/en-gb/search-results/totalseq-a0219-anti-human-cd119-ifn-y-r-a-chain-antibody-16349?Clone=GIR-208
https://www.biolegend.com/en-ie/search-results/totalseq-a0158-anti-human-cd134-ox40-antibody-16437
https://www.biolegend.com/fr-lu/products/totalseq-a0180-anti-human-cd24-antibody-16331?GroupID=BLG5916
https://www.biolegend.com/de-de/products/totalseq-a0139-anti-human-tcr-gammadelta-antibody-15935?6799
https://www.biolegend.com/nl-nl/products/totalseq-a0398-anti-human-cd115-csf-1r-antibody-16581
https://www.biolegend.com/fr-fr/search-results/totalseq-a0164-anti-human-cd1d-antibody-16353?GroupID=BLG9098
https://www.biolegend.com/fr-fr/antibodies-and-more/totalseq-a0242-anti-human-cd192-ccr2-antibody-16359
https://www.biolegend.com/en-us/punchout/search-results/totalseq-a0068-anti-human-cd105-antibody-16346?GroupID=BLG5113
https://www.biolegend.com/de-de/products/totalseq-a0400-anti-human-cd144-ve-cadherin-antibody-16789
https://www.biolegend.com/fr-lu/products/totalseq-a0397-anti-human-cd193-antibody-ccr3-16945?GroupID=BLG2335
https://www.biolegend.com/fr-lu/products/totalseq-a0383-anti-human-cd55-antibody-16493
https://www.biolegend.com/en-us/search-results/totalseq-a0187-anti-human-cd79b-igβ-antibody-16433?GroupID=BLG7860&Clone=CB3-1
https://www.biolegend.com/fr-fr/products/totalseq-a0064-anti-human-cd123-16232?GroupID=BLG2027
https://www.biolegend.com/de-at/products/totalseq-a0053-anti-human-cd11c-antibody-16231?GroupID=BLG15277
https://www.biolegend.com/en-gb/products/totalseq-a0217-anti-human-cd54-antibody-16354?GroupID=BLG9563
https://www.biolegend.com/de-de/products/totalseq-a0215-anti-human-cd268-baff-r-antibody-16358?GroupID=BLG8302
https://www.biolegend.com/en-gb/products/totalseq-a0428-anti-human-tim-4-antibody-16891?GroupID=GROUP28
https://www.fishersci.com/shop/products/totalseq-tm-a0402-anti-hu-cd1/502072649
https://www.biolegend.com/nl-nl/search-results/totalseq-a0401-anti-human-cd301-clec10a-antibody-17064
https://www.biolegend.com/fr-lu/products/totalseq-a0404-anti-human-cd63-antibody-16616?GroupID=BLG10250
https://www.biolegend.com/en-gb/products/totalseq-a0125-anti-human-cd44-antibody-16492
https://www.biolegend.com/fr-ch/search-results/totalseq-a0384-anti-human-igd-antibody-17084
https://www.biolegend.com/nl-be/products/totalseq-a0419-anti-human-cd72-antibody-16533?GroupID=BLG4185
https://www.biolegend.com/fr-ch/products/totalseq-a0446-anti-human-cd93-antibody-16498?GroupID=BLG10342
https://www.biolegend.com/nl-nl/products/totalseq-a0408-anti-human-cd172a-sirpalpha-antibody-16942?GroupID=BLG15632
https://www.biolegend.com/fr-lu/products/totalseq-a0405-anti-human-cd284-tlr4-antibody-16633
https://www.fishersci.com/shop/products/totseqa0406-anti-hu-cd304-10/502077373
https://www.biolegend.com/fr-lu/products/totalseq-a0407-anti-human-cd36-antibody-16651
https://www.biolegend.com/de-de/products/totalseq-a0244-anti-human-cd102-icam-2-antibody-16497?GroupID=BLG8577
https://www.biolegend.com/fr-ch/search-results/totalseq-a0224-anti-human-tcr-alphabeta-antibody-16627?GroupID=GROUP28&Clone=IP26
https://www.biolegend.com/fr-lu/products/totalseq-a0246-anti-human-cd122-il-2rbeta-antibody-22139?GroupID=BLG10194
https://www.biolegend.com/fr-lu/products/totalseq-a0359-anti-human-cd83-antibody-16649
https://www.biolegend.com/fr-lu/products/totalseq-a0247-anti-human-cd267-taci-antibody-16943
https://www.biolegend.com/ja-jp/products/totalseq-a0155-anti-human-cd107a-lamp-1-antibody-16531?Clone=H4A3
https://www.fishersci.com/shop/products/totalseq-a0134-anti-human-cd14-1/502077964
https://www.biolegend.com/de-at/products/totalseq-a0129-anti-human-cd140b-pdgfr-beta-antibody-16940?Clone=18A2
https://www.fishersci.com/shop/products/totalseq-a0575-anti-hu-cd49a-1/502075798
https://www.biolegend.com/fr-fr/search-results/totalseq-a0584-anti-human-tcr-v-alpha-24-j-alpha-18-inkt-cell-antibody-16944?GroupID=BLG7367
https://www.biolegend.com/fr-fr/clone-search/totalseq-a0579-anti-human-cd9-antibody-16631?GroupID=BLG9915

https://www.fishersci.com/shop/products/totalseq-a0581-anti-hu-tcr-va7/502077106
https://www.biolegend.com/nl-nl/search-results/totalseq-a0583-anti-human-tcr-vgamma9-antibody-16755
https://www.biolegend.com/fr-lu/products/totalseq-a0576-anti-human-cd49d-antibody-16769
https://www.biolegend.com/en-gb/products/totalseq-a0577-anti-human-cd73-ecto-5-nucleotidase-antibody-16773?GroupID=BLG7814
https://www.biolegend.com/nl-nl/products/totalseq-a0592-anti-human-cd158b-j-kir2dl2-l3-s2-antibody-17195
https://www.biolegend.com/en-gb/products/totalseq-a0805-anti-human-cd226-dnam-1-antibody-17192?GroupID=BLG10058
https://www.biolegend.com/nl-nl/products/totalseq-a0804-anti-human-cd186-cxcr6-antibody-17188
https://www.biolegend.com/fr-lu/products/totalseq-a0600-anti-human-cd158f-kir2dl5-antibody-17194?GroupID=BLG10199
https://www.biolegend.com/de-at/products/totalseq-a0599-anti-human-cd158e1-kir3dl1-nkb1-antibody-17149
https://www.biolegend.com/en-ie/products/totalseq-a0128-anti-human-cd140a-pdgfralpha-antibody-16488?GroupID=BLG5119
https://www.biolegend.com/ja-jp/products/totalseq-a0586-anti-human-cd354-trem-1-antibody-20597?GroupID=BLG10287
https://www.biolegend.com/de-de/products/totalseq-a0582-anti-human-tcr-vdelta2-antibody-17197?GroupID=BLG13659
https://www.biocompare.com/9776-Antibodies/14500419-TotalSeq-TM-A0588-CD202b-Tie2-Tek-Antibody/?pda=9776%7C14500419_0_1%7C%7C1%7CAb33&dfp=true
https://www.biolegend.com/fr-fr/search-results/totalseq-a0175-anti-human-cd96-tactile-antibody-16150?GroupID=BLG10190
https://www.fishersci.com/shop/products/totalseq-a0420-anti-hu-cd158-1/502076402
https://www.biolegend.com/nl-nl/products/totalseq-a0801-anti-human-cd337-nkp30-antibody-17541
https://www.biolegend.com/nl-be/products/totalseq-a0803-anti-human-cd253-trail-antibody-17540?GroupID=BLG10388
https://www.biolegend.com/fr-ch/products/totalseq-a0830-anti-human-cd319-cracc-antibody-17140?Clone=162.1
https://www.biolegend.com/en-ie/products/totalseq-a0590-anti-human-cd305-lair1-antibody-17279?GroupID=ImportedGROUP1
https://www.biolegend.com/nl-be/products/totalseq-a0433-anti-human-cd325-n-cadherin-antibody-17722?GroupID=GROUP698
https://www.biolegend.com/de-at/products/totalseq-a0853-anti-human-cd371-clec12a-antibody-17518?GroupID=BLG9728
https://www.biolegend.com/de-de/antibodies-and-more/totalseq-a0060-anti-human-cd90-thy1-antibody-15928
https://www.biolegend.com/en-gb/products/totalseq-a0008-anti-human-cd273-b7-dc-pd-l2-antibody-15932
https://punchout.biolegend.com/nl-be/products/totalseq-a0170-anti-human-cd272-btla-antibody-16154
https://punchout.biolegend.com/en-us/products/totalseq-a0138-anti-human-cd5-16333
https://www.biolegend.com/en-gb/products/totalseq-a0897-anti-human-cd23-antibody-18091?Clone=EBVCS-5
https://www.biolegend.com/nl-be/products/totalseq-a0896-anti-human-cd85j-ilt2-antibody-18155
https://www.biolegend.com/fr-ch/clone-search/totalseq-a0867-anti-human-cd94-antibody-18092?GroupID=BLG8469
https://www.biolegend.com/de-de/products/totalseq-a0154-anti-human-cd27-antibody-16174?GroupID=BLG10174
https://www.biolegend.com/fr-ch/products/totalseq-a0902-anti-human-cd328-antibody-19022?GroupID=BLG6362
https://www.biolegend.com/en-gb/products/totalseq-a0920-anti-human-cd82-antibody-23473
https://www.biolegend.com/fr-lu/products/totalseq-a0083-anti-human-cd16-antibody-15765
https://www.biolegend.com/en-ie/products/totalseq-a0423-anti-human-mertk-antibody-16496
https://www.fishersci.com/shop/products/totalseq-a0375-anti-human-igg/502079891
https://www.biolegend.com/de-de/products/totalseq-a0352-anti-human-fceria-antibody-16357?GroupID=BLG6051
https://www.biolegend.com/fr-lu/products/totalseq-a0007-anti-human-cd274-b7-h1-pd-l1-antibody-16195?GroupID=BLG5404
https://www.biolegend.com/en-gb/products/totalseq-a0356-anti-human-cd254-trance-rankl-antibody-16526?GroupID=GROUP28
https://www.biolegend.com/fr-ch/search-results/totalseq-a0179-anti-human-cx3cr1-antibody-18815
https://www.fishersci.com/shop/products/totseqa0022-anti-hu-cd137l-4/502073520
https://www.fishersci.com/shop/products/totalseq-a0199-anti-hu-siglec/502377699

Plants

Seed stocks

Report on the source of all seed stocks or other plant material used. If applicable, state the seed stock centre and catalogue number. If plant specimens were collected from the field, describe the collection location, date and sampling procedures.

Novel plant genotypes

Describe the methods by which all novel plant genotypes were produced. This includes those generated by transgenic approaches, gene editing, chemical/radiation-based mutagenesis and hybridization. For transgenic lines, describe the transformation method, the number of independent lines analyzed and the generation upon which experiments were performed. For gene-edited lines, describe the editor used, the endogenous sequence targeted for editing, the targeting guide RNA sequence (if applicable) and how the editor was applied.

Authentication

Describe any authentication procedures for each seed stock used or novel genotype generated. Describe any experiments used to assess the effect of a mutation and, where applicable, how potential secondary effects (e.g. second site T-DNA insertions, mosiacism, off-target gene editing) were examined.

Flow Cytometry

Plots

Confirm that:

- [x] The axis labels state the marker and fluorochrome used (e.g. CD4-FITC).
- [x] The axis scales are clearly visible. Include numbers along axes only for bottom left plot of group (a 'group' is an analysis of identical markers).
- [x] All plots are contour plots with outliers or pseudocolor plots.
- [x] A numerical value for number of cells or percentage (with statistics) is provided.

Methodology

Sample preparation	A 37-parameter CyTOF panel was designed (Supplementary Table 3). All mass cytometry antibodies were conjugated in-house to their corresponding metal isotope. Metals were conjugated according to the manufacturer's instructions (Fluidigm, South San Francisco, CA, USA). In brief, this process consisted of loading the metal to a polymer for 1h at RT. The unconjugated antibody is transferred into a 50 kDA Amicon Ultra 500 V-bottom filter (Fisher Scientific, Hampton, NH, USA) and reduced for 30 min at 37°C with 1:125 dilution of Tris (2-carboxyethyl) phosphine hydrochloride (TCEP) (ThermoFisher, Waltham, MA, USA). Subsequently, the column was washed twice with C-buffer (Fluidigm) and the metal-loaded polymer was suspended in 200 µL of C-buffer in the 3 kDA Amicon Ultra 500 mLV-bottom filter. The suspension was transferred to the 50 kDA filter containing the antibody and incubated for 1.5h at 37°C. After incubation, antibodies were washed three times with W-buffer (Fluidigm) and quantified for protein content using Nanodrop. Once the concentration was determined, the antibodies were resuspended at a concentration of 0.2 mg/mL with Antibody Stabilizer (Boca Scientific, Dedham, Ma, USA) and stored at 4°C. Optimal concentrations for all antibodies were determined by different rounds of titrations. The staining protocol was optimized to use each antibody in aliquots of 6 million cells as previously described. CyTOF was performed on paired peripheral blood and colon biopsies obtained from the primary case control study (n=12) and from the secondary remission case control study (n=17). Single-cell suspensions of colon biopsies from the right and left colon were pooled to ensure sufficient cell counts per donor. Dead cells were labeled with Cisplatin (Fluidigm) according to the manufacturer's instructions, washed in wash buffer (PBS, 0.5% BSA, 5mM EDTA), fixed in 1.6% PFA for 10 min, washed in wash buffer and then resuspended in freezing medium (PBS, 0.5% BSA, 10% DMSO) and stored at -80oC until staining. Samples were then thawed and washed with wash buffer. Prior to staining with the antibody panel (Supplementary Table 3), cells from each patient were barcoded using a unique set of metals, enabling sample identification as previously described. The barcode staining was performed following the manufacturer's instructions (Fluidigm, South San Francisco, CA, USA). Briefly, each sample was incubated for 15 min at RT on a shaker (200rpm) with a barcoding solution containing 10 µL of barcode in 1x Perm Buffer solution (Fluidigm, Cat#201057) diluted in cell staining media (CSM, Fluidgim, Cat#201068). Samples were then washed, centrifuged, resuspended in CSM, and pooled. Subsequently, extracellular staining was performed for 30 min at 4°C. After incubation, the samples were washed with CSM and centrifuged before resuspending in 1x Permeabilization Buffer (eBioscience™ Permeabilization Buffer Cat# 00-8333-56) for 10min at 4°C. The samples were then washed and incubated with lr-intercalator (Biolegend CNS, San Diego, CA, USA) diluted 1:500 in 4% fresh PFA for 20 min at RT. After incubation, samples were washed and kept at 4°C overnight in EQTM bead solution (Fluidigm Cat#201078) diluted in MaxPar Water (Fluidigm Cat# 201069) at 1.2x106 cells/mL. Samples were analyzed on the CyTOF®2 instrument (Fluidigm).
Instrument	CyTOF®2
Software	CyTOF data were concatenated, normalized and de-barcoded using CyTOF software (Fluidigm); supervised analysis using FlowJo software (10.8.1); unsupervised analysis using an R-based Cytometry Clustering Optimization aNd Evaluation (Cyclone) pipeline developed by the UCSF Data Science CoLab (https://github.com/UCSF-DSCOLAB/cyclone).
Cell population abundance	See Supplemental Table 6
Gating strategy	See Supplemental Table 4 and Extended Figure 10

- [x] Tick this box to confirm that a figure exemplifying the gating strategy is provided in the Supplementary Information.
